# Exploring traditional cosmetic flora from Comoros islands: An ethnobotanical survey in Mayotte

**DOI:** 10.1016/j.heliyon.2024.e35322

**Published:** 2024-07-31

**Authors:** Oumaynou Daroueche, Abassi Dimassi, Cédric Bertrand, François Chassagne

**Affiliations:** a UAR 3278 CRIOBE, EPHE-UPVD-CNRS, Université de Perpignan, 52 Avenue Paul Alduy, 66860 Cedex, Perpignan, France; b Pôle d’Excellence Rurale, Pôle PI2M, RN 2, 97670, Coconi, Mayotte; c Conservatoire botanique national de Mascarin (CBN-CPIE Mascarin), Saint Leu, La Réunion, France; d S.A.S. AKINAO, 52 Avenue Paul Alduy, 66860 Cedex, Perpignan, France; e UMR152 PharmaDev, Université Paul Sabatier, Institut de Recherche pour le Développement, Toulouse, France

**Keywords:** Cosmetopoeia, Mahoran flora, Plants, Africa, Field survey

## Abstract

Mayotte is located in the Indian Ocean and is home to more than five languages, cultures and lifestyles. However, due to rapid urbanization, this traditional knowledge is at risk of extinction. Moreover, ethnobotanical studies on the pharmacopoeia and cosmetopoeia in Mayotte are almost nonexistent. This study was carried out to document the traditional knowledge of Mayotte's cosmetopoeia. The main objective of this study was to document the diversity of cosmetic plants used by the Mahoran community. We conducted field surveys from 2021 to 2022 in 14 communes of “Grande Terre”, the largest of the two islands from Mayotte. A total of 35 experts (*fundi*) were interviewed in this study. Individual interviews with Mahoran informants using open questions were conducted, and voucher specimens were collected for each plant species cited. A total of 470 cosmetic formulations, representing a total of 1777 URs, were recorded. Each formulation contains 1 to 13 ingredients, with a predominance of single-ingredient recipes. In particular, hygiene, makeup, fragrance, hair and nails, and dermatology are the most cited cosmetic categories. A total of 83 plant species were identified and the five most cited plant species were, in decreasing order: *Cocos nucifera* (273 URs), *Jasminum nummulariifolium* (191 URs), *Ocimum* spp. (120 URs), *Curcuma longa* (105 URs), and *Lawsonia inermis* (101 URs). This study is one of the first to focus solely on the exploration of cosmetopoeia in Mayotte, contributing to the preservation of knowledge and the promotion of customs related to traditional cosmetics on this island. Further studies should be performed on some highly cited plant species endemic to the area (e.g., *Jasminum nummulariifolium*, *Pandanus maximus*) to confirm their interest for the cosmetic industry and thus contribute to the economic growth of Mahoran people.

## Highlights

• This is the first survey focusing only on traditional cosmetics from Mayotte island.
• A total of 35 traditional practitioners were interviewed across the island.
• 470 cosmetic recipes representing 1777 use reports were recorded.
• 83 plant species were identified.

## Graphical abstract

**Figure:**
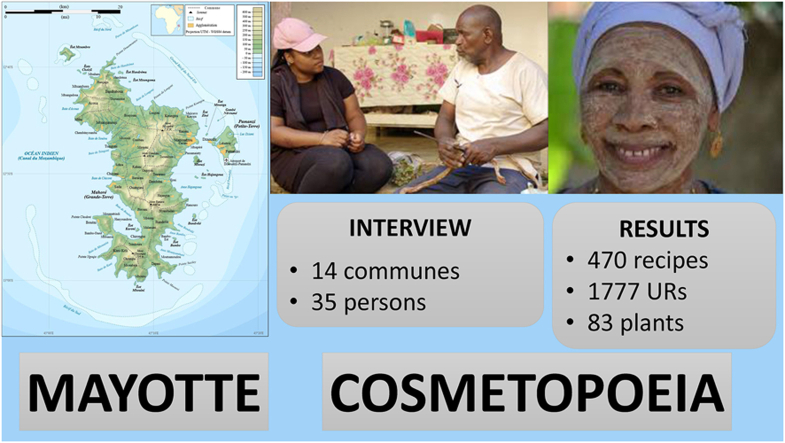

## 1 Introduction

Mayotte is an island located at the northern entrance of the Mozambique Channel, approximately 440 km from the shores of East Africa, 200 km from Anjouan, and 300 km from the west coast of Madagascar. Geographically, it is part of the Comoros archipelago, which consists of four main islands: Grande Comore, Anjouan, Mayotte, and Mohéli [1]. Mayotte is a small volcanic archipelago spanning 374 km^2^, comprised of two main islands and thirteen small islets scattered within a lagoon covering more than 1500 km^2^ [2,3]. The main island, Grande-Terre, is rugged and steep, formed by six eroded massifs, with the highest point being Mount Benara at 660 m above sea level [3].

Historically, Mayotte was initially inhabited by Bantu people originating from Africa. Later, the islands were colonized by Arabic people who introduced Swahili culture and the Muslim religion. In the 15th century, Europeans arrived, and Mayotte ultimately chose to become French after an independence vote in 1974. Since then, Mayotte is a French overseas territory. Situated at the crossroads of Bantu, Arabo-Persian, Austronesian, Indian, and Western influences, Mayotte possess a rich cultural diversity [4].

Within this confined insular space, two local languages prevail: Shimaoré, a Bantu language, and Kibushi, a Malagasy language. In addition to these languages, French is officially used in administration and education, while Arabic is taught in Koranic schools and madrasas. Three variants of Comorian languages contribute to the linguistic diversity of the region: Shindzuani (spoken in Anjouan), Shingazidja (spoken in Grande Comore), and Shimwali (spoken in Mohéli). Although quantitatively less represented, two other languages are also present: Hindi and Reunionese Créole.

Shimaoré belongs to the Bantu language family of the Comoros archipelago, within the North-East Coast group of Africa. It shares similarities with Swahili. Mayotte also has a second main language, Kibushi, which constitutes the only Malagasy variety spoken outside of Madagascar. This presence is explained by the settlement, in the 16th century, of a large number of Sakalava Malagasy in the south of Mayotte. Kibushi is spoken in about ten villages, mainly in the south and on the west coast of Mayotte [1]. The Malagasy language is represented by two distinct varieties. One is the Kibosy Kimaoré, simply meaning “Malagasy of Mayotte” (sometimes used interchangeably to refer to both varieties), and the other is the Kibosy Kiantalaotsy. It is generally asserted that Kibosy Kimaoré is closely related to Northern Madagascar varieties, while Kibosy Kiantalaotsy is very close to the Sakalava dialect spoken in Mahajanga [5]. Almost all of the inhabitants of Mayotte adhere to the Muslim faith. Sunni Islam of the Shafi'i rite is intertwined with pre-Islamic Arab animist beliefs and Bantu traditions [1].

Mayotte Island harbors a rich flora, encompassing 1341 species, including 663 naturally occurring, 44 cryptogenic, and 634 exotic species [2]. Among these plants, some are still used today in traditional medicine and as traditional cosmetics. The latter practice is known as “cosmetopoeia,” which derives its name from the Greek verb “kosmeo” meaning “I adorn, I decorate” [6]. Cosmetopoeia involves the study of traditional uses of raw materials, such as plants or minerals, for cosmetic purposes, akin to the pharmacopoeia of medicinal plants. Traditional practices related to cosmetic plants are deeply rooted within specific communities. For example, Polynesia is renowned for its monoi, while in the Comoros, women are known for their beauty masks called “msindzano”. Cosmetopoeia aims to preserve and protect the richness of this cultural heritage. Defining what falls under cosmetics is crucial. The French National Agency for the Safety of Medicine and Health Products (ANSM) defines cosmetics as “substances or preparations intended to come into contact with various parts of the human body, such as the skin, hair, nails, lips, or even teeth, exclusively or mainly for the purpose of cleaning, perfuming, modifying their appearance, protecting them, keeping them in good condition, or correcting their odors.” [7].

So far, a few research studies have been conducted on the flora of Mayotte for their cosmetic purposes. For example, various authors have focused on an emblematic plant from Mayotte: *Cananga odorata*, which is highly used in cosmetopoeia and exported as a perfume [[8], [9], [10], [11]]. Regarding general studies on cosmetopoeia in Mayotte, three studies have already been published. In the first study, a total of 69 plant species have been described, including 50 used for cosmetic purposes, with insights gathered from 29 participants, mainly from the north and center of Mayotte [11]. In the second study, 19 plants have been documented for cosmetic purposes [12]. In the third one, 15 plants have been documented for cosmetic purposes [13].

Given the richness of Mayotte's cosmetopoeia and the limited research conducted, particularly in the south of the island, we have decided to conduct a survey to inventory the knowledge of Mahoran people regarding the use of traditional cosmetics. Our main research questions include: What are the main plant species used in Mahoran cosmetopoeia? What are the main cosmetic uses reported? Are these plant species little known around the world for their cosmetic uses and could therefore be economically valued by the local population?

## 2 Materials and methods

### 2.1 Study area

Mayotte is one of the islands comprising the Comoros archipelago, located in the northern part of the Mozambique Channel in the Indian Ocean. The entire archipelago covers an area of 2034 km^2^ and consists of four islands: Anjouan, Mohéli, and Grande Comore (Ngazidja), which form the Union of the Comoros, and Mayotte, which is a French department. Mayotte itself comprises Grande-Terre, with an area of 355 km^2^, Petite Terre, spanning 12 km^2^, and 13 islets. The population is estimated to be 310,000 inhabitants.

Due to its geographical location and nature, Mayotte experiences a tropical humid climate with two distinct seasons: dry and wet. These conditions significantly influence the vegetation, which can be categorized into five main types: sub-montane forest, hygrophilous vegetation, mesophilic vegetation, semi-xerophilous vegetation, and coastal and marine vegetation [14].

**Fig. 1:**
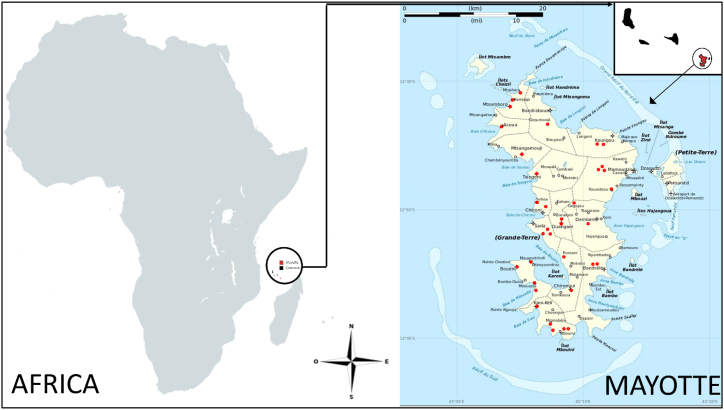
Map showing Mayotte in Africa and in the Indian Ocean (red dots: visited sites). (For interpretation of the references to color in this figure legend, the reader is referred to the Web version of this article.)

The survey was conducted on Grande-Terre, which comprises 16 municipalities. Of these, 14 were explored: Kani-Kéli, Bouéni, Bandrélé, Chirongui, Dembéni, Ouangani, Chiconi, Sada, M'tsangamouji, M'tsamboro, Acoua, Bandraboua, Mamoudzou, and Koungou (Fig. 1).

### 2.2 Data collection

The study was conducted between April and June 2021 and July to September 2022. Interviews were conducted in various languages, namely French, Shimaore, and Kibushi, depending on the preferences of each interviewer. The first author conducted these interviews with the assistance of a key translator.

The first part of the study involved interviewing individuals known through word of mouth. Subsequently, several villages were visited, with the goal of inquiring about the presence of knowledgeable individuals, referred to as *fundi* or people known for using plants in cosmetics. The term *fundi*, whose primary meaning is that of a Koranic schoolmaster, is extended to refer to a scholar or expert in a particular field [15]. They are recognized as experts in their respective fields and hold a high social status in the Mahoran community, commanding great respect. Some *fundi* specialize in traditional medicine, enabling them to treat ailments of religious, spiritual, or physical origin. Additionally, individuals who are known as defenders and transmitters of ancestral knowledge on the island are also classified as *fundi*.

The data were collected using a semi-structured questionnaire that included open-ended questions. The questionnaire was organized into the following sections:

• Sociodemographic data: Age, gender, place of residence, occupation, and religion.
• General information about their knowledge of plants: Source and origin of knowledge.
• Information on traditional cosmetic recipes used for different targets: phanera (e.g., nails, hair), body, and face. The questions centered around the plants and their usage for each target to determine if they were used for care, hygiene, makeup, protection, or beautification. For each cited recipe, data on ingredients used (e.g., vernacular name of plant species), part of ingredients, associated cosmetic claims, preparation method, administration method, and usage precautions were collected.
• Other questions focused on plants used for medicinal purposes, which could be extrapolated into cosmetics, such as treating issues like heat rashes, toothaches, skin blemishes, and so forth.

### 2.3 Botanical identification

Plants mentioned by participants were collected in triplicate and stored in the herbarium at the Rural Excellence Pole (PER) in Coconi village. The validation of their scientific names was conducted with the assistance of the second author of this article, a botanist and one of the specialists of Mayotte's flora.

All plant names have been verified and updated according to international and local databases, such as Plants of the World Online. (https://powo.science.kew.org/), and the World Flora Online (https://www.worldfloraonline.org/).

### 2.4 Ethics

Adopted in October 2010, the Nagoya Protocol to the Convention on Biological Diversity, an international biodiversity agreement, came into effect on October 12, 2014. It primarily addresses access to genetic resources and traditional knowledge associated with genetic resources and the fair and equitable sharing of benefits arising from their utilization (ABS) while establishing an international legal framework. In France, the Nagoya Protocol does not require declarations for access to traditional knowledge on French territory (except for French Guiana and Wallis and Futuna) [16]. The project was explained to each participant, detailing its objectives and the significance of collecting data on traditional knowledge. Prior informed consent was obtained from each participant before each interview. It is noteworthy that this study is directly related to the Rural Excellence Pole of Coconi, specifically the Integrated Innovation Pole of Mayotte (PI^2^M). Its objective is to actively support innovation and sustainable development in Mayotte, particularly through the “Green” aspect, which focuses on the economic and sustainable valorization of the terrestrial resources of Mayotte, with a particular focus on aromatic perfumes and medicinal plants (APMP).

### 2.5 Data analysis

A database containing all the information gathered during field surveys (recipes, plants, scientific names, common and vernacular names, cosmetic use, claims, and plant parts used) was compiled in an Excel spreadsheet. Before conducting the analysis, standardization was carried out in terms of claims and plant parts. For example, phrases like “apply to the body,” “apply to the skin,” or “apply on the skin” were standardized as “apply to the body."

The use report, or UR, can be described as an informant (i) mentioning the use of a species (s) for the preparation of a recipe (r). In this study, we followed the following method to convert the data into use reports. If species ‘A' was recommended for cosmetic claim ‘x,’ it was considered as one UR. If species ‘A' was recommended for cosmetic claims ‘x' and ‘y,’ then it was considered as two reports, that is, two URs. If a mixture of species ‘A' and ‘B' was used for cosmetic claim ‘x,’ it was considered as two URs (i.e., species ‘A' for cosmetic claim ‘x' and species ‘B' for cosmetic claim ‘x'). If a mixture of species ‘A' and ‘B' was used for cosmetic claims ‘x' and ‘y,’ it was considered as four (2 × 2) URs. In this way, all the data were converted into URs [17].

The most cited plants were searched in terms of their chemical composition and biological activity using various databases such as Pubmed, Bibtex CNRS, Web of Science, and Google Scholar.

## 3 Results

### 3.1 Socio-demographic data and classification of healers

A total of 35 participants were interviewed (Table 1), among which 13 were interviewed in the northern part of the island. Specifically, one person was interviewed in Acoua, one in Bandraboua, two in Koungou, four in Mamoudzou, three in Mtsamboro, one in Mtsangamouji, and one in Tsingoni. In the central area, nine individuals were questioned, distributed between two in Chiconi, two in Dembéni, two in Ouangani, and three in Sada. Finally, in the southern region, 13 individuals were interviewed, including two in Bandrélé, four in Bouéni, two in Chirongui, and five in Kani-Kéli. A total of 18 females and 17 males were interviewed. All them were muslims, and most of them did not have any occupation.

**Table 1:** Sociodemographic characteristics of the 35 persons interviewed in Mayotte.

Characteristics	Frequency	Percentage (%)
Gender		
Female	18	51
Male	17	49
Residence		
North	13	37
Center	9	26
South	13	37
Occupation		
Business	4	11
Craft person	2	6
Farmer	3	8
Masseuse	2	6
Nurse	1	3
Nurseryman	2	6
None	17	48
Other	2	6
Teacher	2	6
Religion		
Muslim	35	100
Language		
Kibushi	16	46
Shimaoré	19	54

### 3.2 Cosmetics recipes and uses

The 35 interviews yielded a total of 470 recipes, representing 704 citations. This study focuses on cosmetic uses and their associated claims. However, some mentioned plants are more commonly used for medicinal purposes and have been included in cases where their use is topical (e.g., for toothaches or mouthcare). Conversely, all other plants exclusively used for medicinal purposes and requiring ingestion have been excluded from the study.

The number of ingredients per recipe varies from 1 to 13. Among all the listed recipes, 293 are composed of only one ingredient, 108 have two ingredients, 41 have three ingredients, nine have four ingredients, eight have five ingredients, one has six ingredients, two have seven ingredients, four have eight ingredients, two have ten ingredients, one has twelve ingredients, and one has thirteen ingredients.

All the cosmetic claims have been classified into five groups based on Ansel et al.'s classification [6]. These five groups are as follows.

• First group claim: Dermatology. It includes all cosmetic uses with a medicinal aspect.
• Second group claim: Action on epidermis. It includes all cosmetic recipes that affect the outmost layer of the skin (care, maintenance and regeneration).
• Third group claim: Skin pigmentation. It includes all cosmetic recipes acting on skin color and skin marks.
• Fourth group claim: Hair and nails. It includes all cosmetic recipes dedicated to the care, coloration and maintenance of hair and nails.
• Fifth group claim: Hygiene, makeup, perfume. It includes all cosmetic recipes used for hygiene, look and appearance.

We have made some modifications to the original classification. Deodorant and antiperspirant, initially classified in the first group, have been moved to the fifth group. Based on the identified recipes, we conducted a preliminary classification of cosmetic claims, resulting in 25 claims for group 1 (90 citations), 13 claims for group 2 (112 citations), 10 claims for group 3 (33 citations), 25 claims for group 4 (103 citations), and 26 claims for group 5 (367 citations). (Table 2).

**Table 2:** Cosmetic uses, mentioned by the 35 participants, classified according to the five cosmetic target groups.

First group claim: Dermatologically related	Number of citations	Second group claim: Care and maintenance, hygiene, and regeneration of the dermis and skin	Number of citations	Third group claim: Skin pigmentation	Number of citations	Fourth group claim: Appendages (hair, nails)	Number of citations	Fifth group claim: Hygiene, makeup, perfume, and ingredients	Number of citations
Anti-pimple	46	Moisturizes the skin/body	31	Adds shine	13	Nourishes the hair	23	Beautifies	119
Treats dermatoses	6	Softens the skin/body	18	Colors the lips	4	Shampoo/Hair soap	15	Perfume	105
Treats pimples and patches caused by allergy	5	Anti-aging/Preserves youth/Rejuvenates	16	Brightens the complexion	4	Activates hair growth	14	Cleans	34
Heals wounds/injuries	4	Nourishes the skin/body	14	Anti-spots	3	Detangles the hair	8	Deodorant	21
Against pimples and redness in children (babies) or individuals with sensitive skin	3	Heals cracks	7	Lightens scars	2	Hair growth	6	Facial beauty	14
Accelerates the healing of wounds/injuries	3	Used to firm up the skin	6	Gives a healthy glow	2	Perfumes the hair	5	Intimate hygiene	10
Against pimples with patches	2	Heals chapped skin	6	Beautifies the complexion	2	Depilatory care	4	Makeup	9
Hemostatic	2	Body care	5	Tints the skin	1	Good hair health	3	Enhances the gaze	8
Antiseptic	2	Massage	4	Removes imperfections	1	Nail care	3	Well-being	5
Allergies with skin eruption	2	Beauty mask	2	Colors the cheeks (msindzano)	1	Hair oil	3	Face painting for ceremonies	5
Accelerates wound healing	1	Facial care	1			Softens the hair	2	Hygiene	5
Against pimples and a condition called “mwili wa moro."	1	Dries out pimples	1			Lengthens curls	2	Refreshes the skin	5
Anti-acne	1	Finger care	1			Anti-dandruff	2	Stimulates/tones the body	5
Anti-irritation	1					Scalp ringworm	2	Energizes the body	3
In case of diaper rash in children	1					Softens the hair	1	Beautifies the gaze	3
In case of scabies	1					Hair beauty	1	Protects against the evil eye	3
In case of allergies causing pink patches	1					Capillary	1	Bad breath	2
Treats bumps on the tongue caused by small sores	1					Decorates hands and feet	1	Invigorates the body/mind	2
Furuncles	1					Protects the hair	1	Body soap	2
Cleansing like Betadine	1					Refreshes the scalp	1	Captivates the attention of men	1
Anti-itch	1					Hair care	1	Oral cleanser	1
Promotes wound healing	1					Post-depilatory care	1	Revives the skin	1
Antimicrobial	1					Relieves post-depilation	1	Oral care	1
Against gingivitis	1					Prevents hair loss	1	Body painting	1
Itching with heat rash	1					Used for hair removal preparation	1	Exfoliation	1
								Mouthwash	1

**Fig. 2:**
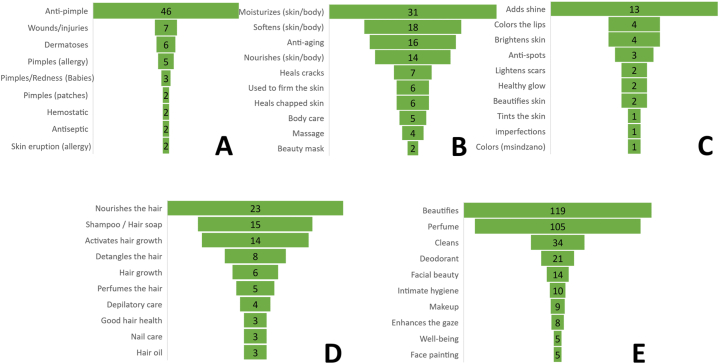
Most cited uses for the five different cosmetic groups. A. Group 1: Dermatology. B. Group 2: Action on epidermis. C. Group 3: Skin pigmentation. D. Group 4: Hair and nails. E. Group 5: Hygiene, makeup, perfume.

In the first category (dermatology), the anti-pimple properties is the most cited cosmetic claim with 46 citations, followed by dermatoses with six citations, pimples, and patches with five citations, and healing wounds and injuries with four citations (Fig. 2A). In the second category (action on epidermis), four claims predominate, listed in descending order: moisturizes skin/body with 31 citations, smoothens skin/body with 18 citations, anti-aging with 16 citations, and nourishes skin/body with 14 citations (Fig. 2B). Within the third category, the top four claims, in descending order, are: shining effect with 13 citations, brightens complexions and colors the lips with 4 citations, and anti-spots with 3 citations (Fig. 2C). In the fourth category, the four most frequently cited claims, in descending order, are: nourishes the hair with 23 citations, shampoo/hair soap with 15 citations, activates hair growth with 14 citations, and detangles the hair with 8 citations (Fig. 2D). Lastly, within the fifth category, the five most cited claims, in descending order, are: embellishes with 119 citations, perfumes with 105 citations, cleans with 34 citations, deodorizes with 21 citations, and facial beauty with 14 citations (Fig. 2E).

In Mahoran cosmetopoeia, a multitude of ingredients are used to make the cosmetic products. Among the liquids, Pompeia®, a scented lotion, is the most represented with 8 citations, followed by rose water (6 citations), petroleum (3 citations), *ranou laka* (water from the boat) (1 citation), Rêve d'or®, another scented lotion (1 citation), and sea water (1 citation). Mixed vegetal substances include *gwéna* (23 citations), *zoukouba* (9 citations), *manu kantru* (5 citations), Marseille's soap (4 citations), and *oubani* (1 citation) (see below for more details). Ingredients of animal origin are exemplified by the presence of beeswax (18 citations), and honey (2 citations). Finally, mineral compounds include white clay (5 citations), salt (3 citations), charcoal (2 citations), red clay (2 citations), *vatou mogné* (natural pumice) (2 citations), and violet clay (1 citation).

### 3.3 Description of typical mahoran cosmetic recipes

#### 3.3.1 Zoukouba

**Fig. 3:**
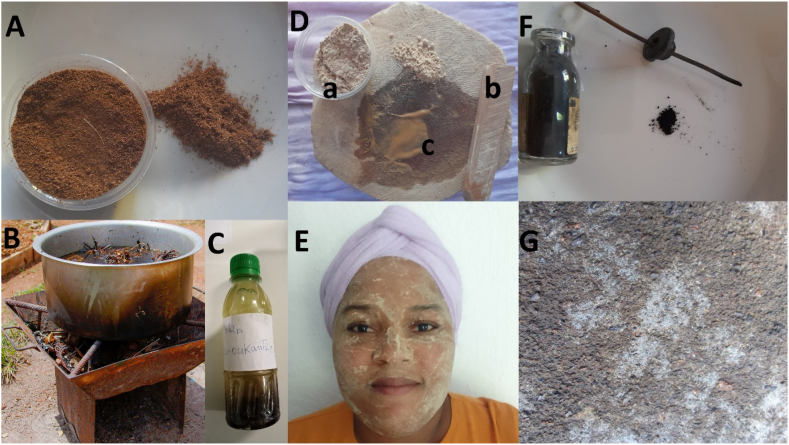
Pictures of typical mahoran cosmetics. A: Powder of *zoukouba* (blend of fragrant plants). B: Preparation of *matcha manu kantru* (fragrant preparation). C: *Matcha manu kantru*. D.a: Powder of piece of wood of *msindzano* (beauty mask). D.b: *Ka* (piece of wood). D.c: *Msindzano*. E: Mask of *msindzano*. F: *Gwéna* (eye pencil). G: *Vatou mogné* (natural pumice).

*Zoukouba* refers to a blend of fragrant plants. The plants used are dried either in the shade or in the sun. Subsequently, they are pounded in a large mortar, known as *shino* in Shimaoré, until the desired consistency is achieved. Finally, the mixture is placed in pots (Fig. 3A). It is utilized in the production of *matcha manu kantru* (Fig. 3B and C) or can be incorporated when creating beauty masks, such as the *msindzano* (Fig. 3D and E), for scented products, for instance.

#### 3.3.2 Matcha manu kantru

*Matcha manu kantru* derives from the Anjouanese language, where *matcha* signifies “oil”, and *manu kantru* from Anjouanese origin means “it smells good” or “fragrant”. The preparation consists of using coconut milk extracted from *Cocos nucifera*. The coconut milk is cooked until it transforms into oil. Subsequently, a scented lotion named Pompeia ® or Rêve d'or® is added, along with a mixture of dried aromatic plants which is left to macerate. Among these plants are rhizomes of *Chrysopogon zizanioides,* *Plumeria alba* flowers*, Jasminum nummulariifolium* flowers*, Pandanus maximus* flowers*, Rosa alba* flowers*, Vachellia farnesiana* flowers, leaves and inflorescences of *Ocimum* sp.*,* leaves of *Pogostemon cablin,* and flowers of *Cananga odorata.* The resulting preparation is then bottled and used as a fragrance at weddings. Alternatively, it can be directly applied as a macerated scent.

#### 3.3.3 Msindzano

*Msindzano* refers to a beauty mask, typically crafted from various wooden rods scraped on dead coral stone with a small amount of water until a smooth paste is achieved. This paste is then applied to the face as anti-pimple care, for sun protection, or ornamental use. Among the woods recognized for making these masks are *Santalina madagascariensis* Baill.*, Radamaea montana* Benth., the “*Ka*” (a piece of wood of unknown origin) (Fig. 3Db.) and *Carissa spinarum*. Additionally, plants like *Curcuma longa* are used to add color and promote a tanned complexion. Masks can also be made from *Persea americana* seeds for hydrating and nourishing the face. Some plant, such as *Pityrogramma calomelanos*, are used for patternmaking by directly applying the leaf's side with the bloom to the face, serving as a base for patterns. Kaolin is another substance used for pattern creation. These last two substances can be used independently or in combination with *msindzano*. It's worth noting that the wooden rods used in crafting these masks present a challenge, particularly in determining their geographical origins, which are uncertain and may be traced back to either Africa or Madagascar.

#### 3.3.4 Gwéna

*Gwéna* is a black pencil used for the eyes, prepared by igniting a locally known incense resin charcoal, called *oubani*, then covering it with a bowl to capture the resulting smoke. The particles are collected on the bowl, forming a black layer. This layer is scraped off and placed in a mini bottle with cotton or in a handmade package filled with cotton. A piece of iron is included in the bottle to pick up the powder, which is then applied to the eyes (Fig. 3F). In addition to its beautifying effect on the eyes, *gwéna* is applied when eyes are sore and is also used to ward off the evil eye, which is why it is often applied to the faces of newborns.

### 3.4 Plants used as cosmetics in Mayotte

**Fig. 4:**
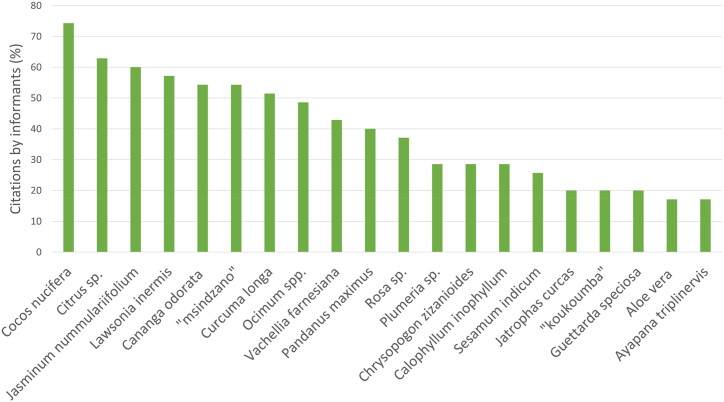
Most cited plant species and preparations used as cosmetics in our survey.

**Fig. 5:**
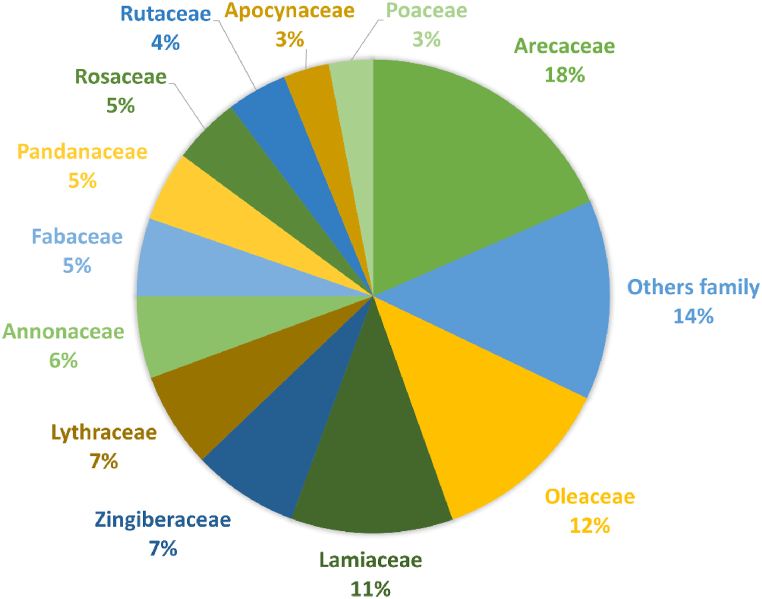
Frequency of citations of the most cited botanical families.

A total of 83 plant species were identified, belonging to 46 botanical families, representing a total of 1777 URs (Table 3 & Suppl. Mat. Table 1). The ten most cited plant species were, in decreasing order: *Cocos nucifera* (273 URs), *Jasminum nummulariifolium* (191 URs), *Ocimum* spp. (120 URs), *Curcuma longa* (114 URs), *Lawsonia inermis* (108 URs), *Cananga odorata* (75 URs), *Pandanus maximus* (69 URs), *Rosa alba* (69 URs), *Vachelia farnesiana* (72 URs), and *Citrus* sp. (65 URs) (Fig. 4). Among the most frequently cited botanical families, we found Arecaceae (273 URs), followed by Oleaceae (191 URs), Lamiaceae (120 URs), Zingiberaceae (109 URs), Lythraceae (102 UR), Annonaceae (75 URs), and Fabaceae (81 URs). In terms of number of species in each family, Fabaceae family ranks first (7 species), followed by Convolvulaceae and Lamiaceae (6 species each), Euphorbiaceae, Malvaceae, and Rutaceae (4 species each), and Apocynaceae, Asteraceae, Lauraceae, Solanaceae and Zingiberaceae (3 species each). In some of the most represented botanical families in terms of URs, only one species is represented. This is the case for the Arecaceae family, for which the sole genus and species is *Cocos nucifera*; the Oleaceae family represented by *Jasminum nummulariifolium*; the Lythraceae family with *Lawsonia inermis*, and the Annonaceae family with *Cananga odorata*. It is noteworthy that the top 11 families collectively contribute to nearly 86 % of all citations, with the three primary families accounting for 41 % (Fig. 5). Notably, half of these families have citations exceeding 100, while the other half falls below that threshold.

**Table 3:** List of plant species used in cosmetic recipes cited in our survey.

Familly	Scientific name	Vernacular name (french)	English name	Vernacular name (mahoran)	Vernacular name (kibushi)	Part of plant used	Cosmetic uses	URs	Voucher specimen
Acanthaceae	*Barleria lupulina* Lindl.	Herbe tac-tac	Hop-headed barleria	Mshari	Mamy lahy				ODAM019
						Leaf	Anti-blemish	1	
Aloeaceae	*Aloe vera* (L.) Burm.f.	Aloès	Aloe	Sakouhakinkigni	Shiizi ya mlili				ODAM018
						Leaf (inner gel)	Promotes healing/Cleanser/Beautifies/Face care/Shampoo/Smoothens/Skin care/Refreshes/Well-being/Preserves youth/Firm up the skin	12	
						Leaf	Promotes hair growth/Moisturizes (face)/Moisturizes the Body/skin	6	
Amaranthaceae	*Achyranthes aspera* L.	Herbe d'Eugène	ND	Tsoho	Tsoho				ODAM030
						Leaf	Soothes/Heal	2	
Amaryllidaceae	*Allium schoenoprasum* L.	Oignon vert	Green onion	Shourougou ya mani	Dugulu basoili				NC
						Leaf	Cleanser/Anti-itch	3	
Amaryllidaceae	*Allium* sp.	Ail	Garlic	Shourougou voushé	Dugulu layii				NC
						Leaf	Cleanser/Anti-itch	2	
Anacardiaceae	*Anacardium occidentale* L.	Anacardier	Cashew	Mabibo	ND				ODAM031
						Bark	Toothache (mouthcare)	1	
						Bark and leaf	Toothache (mouthcare)/Gingivitis	2	
Annonaceae	*Cananga odorata* (Lam.) Hook.f. & Thomson	Canang odorant	Ylang-ylang	Langilang	Langilang				ODAM025
						Flower	Fragrance/Beautifies/Perfumes oil/Massage/Softens/Post-depilatory care/Refreshes/Preserves youth/Well-being/Nourishes/Brightens/Hydrates/Essential oil	75	
Apocynaceae	*Carissa spinarum* L.	Bois amer	ND	M'djanfari	Taola na omby				NC
						Piece of wood (stem)	Beauty mask/Sun protection	2	
Apocynaceae	*Plumeria* sp L.	Frangipanier	Nosegay Tree	ND	Ngaya bé				ODAM032; ODAM033
						Flower	Beautifies/Fragrance/Nourishes/Brightens/Softens/Moisturizes/Well-being	38	
Arecaceae	*Cocos nucifera* L.	Cocotier	Coconut	M'nadzi	Voaniho				ODAM034
						Fruit (coprah)	Cleanser/Soap/Shampoo	5	
						Fruit (milk*)	Anti pimples/Cleanser/Brightens/Add shine/Beautifies/Nourishes/Tanned complexion/Softens/Detangles/Shampoo	45	
						Fruit (milk*, pulp)	Cleanser/Hydrates/Softens/Well-being	6	
						Fruit (oil**)	Moisturizes/Smoothens/Nourishes/Strengthens/Stimulates hair growth/Softens/Well-being/Wound, Wound protect/Oil of hair/Add shine/Hygiene (soap)/Skin care/Hair care/Sunburn/Warms the skin, face/Cleanser/Massage/Anti pimples/Food allergies causing skin rashes or not, Allergies forming sort of plaques/Skin disorders/Strengthens/Refreshes/Anti aging/Firm up the skin/Depilatory care, Post-depilatory care/Protect/Fragrance/Lightens/Relaxes, loosens the muscles/Warm the scalp/Relieves/Oil for body/Shampoo	180	
						Fruit (oil**, milk*, grated)	Cleanser/Moisturizes/Nourishes/Softens/Add shine/Beautifies/Well-being/Tanned complexion/Brightens/Smoothens/Skin care	34	
						Fruit (water)	Cleanser/Detangles/Shampoo	3	
Asteraceae	*Ayapana triplinervis* (Vahl) R.M.King & H.Rob.	Ayapana, yapana	Ayapana	Mlaliyapana	Mlaliyapana				ODAM009
						Leaf	Beautifies/Fragrance/Softens/Depilatory care/Nourishes/Add shine/Lightens/Moisturizes	12	
Asteraceae	*Sigesbeckia orientalis* L.	Colle colle	Sigesbeckia herba		Tei lamba				ODAM035
						Whole plant	Anti pimples	1	
Asteraceae	*Youngia japonica* (L.) DC.	Lastron bâtard	ND	ND	Sari féliki guissi				ODAM036
						Leaf	Spots with lesions	1	
Bixaceae	*Bixa orellana* L.	Roucouyer	Annatto	ND	M'jenguéfuré				NC
						Seed	Color lips	4	
Bombacaceae	*Adansonia digitata* L.	Baobab	Baobab tree	Buyu	Buyu				ODAM016
						Fruit (pulp)	Well-being/Cleanser/Softens/Beautifies/Tanned complexion/Brightens/Moisturizes/Anti aging	16	
						Piece of wood (stem)	Anti pimples/Scabies	3	
Burseraceae	*Commiphora arafy* H.Perrier	ND	ND	M'ri ombany	Matyambelo				ODAM037
						Seed	Toothache (mouthcare)	1	
Calophyllaceae	*Calophyllum inophyllum* L.	Takamaka	Alexandrian laurel	M'tondro	M'tondro				ODAM022
						Sap	Depilatory	11	
Convolvulaceae	*Decalobanthus peltatus* (L.) A.R.Simões & Staples	Décalobanthe pelté	Merremia	Vahi bé	Vahi bé				ODAM038
						Leaf	Stimulates hair growth/Add shine/Strengthens/Smoothens/Eliminate vaginal odor/Anti dandruff	7	
Convolvulaceae	*Ipomoea batatas* (L.) Lam.	Patate douce	Sweet potato	Batata	Batata				ODAM039
						Leaf	Cleanser/Nourishes/detangles/Strengthens/Softens/Shampoo	7	
Convolvulaceae	*Ipomoea nil* (L.) Roth	Ipomée du nil	Blue morning-glory	ND	Antaka mawaridi				ODAM041
						Leaf	Accelerates healing	1	
Convolvulaceae	*Ipomoea obscura* (L.) Ker Gawl.	Ipomée obscure	Obscure morning glory	Hovéani	Hovéani				ODAM004
						Leaf	Cleanser/Softens/Strengthens/Smoothens/Nourishes/Stimulates hair growh/Add shine/Anti dandruff/Detangles/Beautifies/Refreshes the scalp/Treat ringworm of the scalp/Shampoo	20	
Convolvulaceae	*Ipomoea pes-caprae* (L.) R.Br.	Patate à Durand	Beach morning glory	ND	Lalanda				ODAM040
						Leaf	Prevent hair loss	1	
Crassulaceae	*Kalanchoe pinnata* (Lam.) Pers.	Kalanchoé penné	ND	Miawani	Sudifafgna				ODAM042
						Leaf	Skin care/Firm up the skin/Beautifies	4	
Cucurbitaceae	*Kedrostis elongata* Keraudren	ND	ND	Bahi bahi	Tumbou antani				NC
						Leaf	Heals the pimples	1	
Euphorbiaceae	*Acalypha indica* L.	Acalyphe d'Inde, herbe chat	Indian acalypha						NC
						Leaf/Stem	Treat ringworm of the scalp	2	
Euphorbiaceae	*Croton mayottae* P. E. Berry & Kainul	Croton glanduleux de Mayotte	ND	Muhuve	Sari laza laza				ODAM043
						Root	Anti pimples (*mwili wa moro*)	2	
Euphorbiaceae	*Jatropha curcas* L.	Pignon d'inde	Purging nut	Muri maji	Valavelo				ODAM029
						Bark	Antimicrobial/Reduces bad breath	2	
						Latex	Hemostatic/Healing	6	
						Seed	Make up	1	
Fabaceae	*Crotalaria laburnoides* Klotzsch	Crotalaire arbuste	ND	ND	Ampamono maso na koho bory				NC
						Fruit (Pod)	Shampoo	1	
Fabaceae	*Hymenaea verrucosa* Gaertn.	Copalier	gum copal tree	Mvoumba	Yembuki				NC
						Bark	Anti pimples/Brightens/Beautifies	3	
Fabaceae	*Indigofera tinctoria* L.	Indigotier	Indigo	M'komba unyo	Heinguitshi				ODAM014
						Leaf	Antiperspirant/Deodorant/Anti pimples	4	
Fabaceae	*Mimosa pudica* L.	Sensitive	Sensitive plant	ND	Fatsiki ambili, Shibalabala masu				ODAM045
						Leaf	Boils	1	
Fabaceae	*Pterocarpus indicus* Willd.	Sang-dragon	Angsana tree	ND	M'sandrago				NC
						Leaf	Shampoo	3	
Fabaceae	*Senna singueana (Delile) Lock*		Winter cassia	M'ri m'buzi	Andra béyi, Sambaravatsi				ODAM044
						Leaf	Allergies causing pinkish patches/Dermatoses	2	
Fabaceae	*Vachellia farnesiana* (L.) Wight & Arn.	Acacia de farnèse	Sweet acacia	Mugu dzidzano	Fu mgu tamotamo				ODAM001
						Flower	Fragrance/Skin care/Cleanser/Softens/Nourishes/Beautifies/Moisturizes/Lightens/Add shine	67	
Lamiaceae	*Ocimum americanum* L.	Basilic citron	American basil	ND	Karandzani mroni				ODAM046
						Leaf	Antiperspirant/Deodorant	2	
Lamiaceae	*Ocimum gratissimum* L.	Basilic grande feuille	African basil	M'rule	Rulé				ODAM005
						Leaf	Cleanser/Reduces vaginal odors/Reduces white discharge/Tightens the vagina/Maintaining the vagina/Tones the vagina/Cleanser	18	
Lamiaceae	*Ocimum* spp.	ND	ND	Mkadi	Mkadi				ODAM007
						Leaf and flower	Fragrance/Beautifies/Skin care/Cleanser/Softens/Nourishes/Moisturizes/Post-depilatory care/Protect/Add shine/Lightens	120	
Lamiaceae	*Plectranthus amboinicus* (Lour.) Spreng.	Gros thym	Cuban oregano	Porouvi	Parauvi				NC
						Leaf	Promotes healing/Wound healing	3	
Lamiaceae	*Pogostemon cablin* Benth.	Patchouli	Patchouli	Patchor	Patchor				ODAM008
						Leaf	Fragrance/Beautifies	9	
Lauraceae	*Cassytha filiformis* L.	Liane Foutafout	Bush-dodder	ND	Tsihitafotoutshou				NC
						Whole plant	Dermatoses	1	
Lauraceae	*Litsea glutinosa* (Lour.) C.B.Robins	Bois d'oiseaux à petites feuille	Bolly-beech	M'zavoca maro	Zavoca maro				ODAM002
						Sap	Promotes healing/Heal burns	3	
Lauraceae	*Persea americana* Mill.	Avocatier	Avocado	Avoka	Avoka				ODAM047
						Seed	Beauty mask/Moisturizes/Beautifies/Anti pimples/Anti-aging/Cleanser	12	
Loganiaceae	*Strychnos spinosa* Lam.	Oranger du Natal	Natal orange	ND	Mkutsha				ODAM048
						Root	Skin conditions	1	
Lythraceae	*Lawsonia inermis* L.	Hénné	Henna	Hina dzishe	Mwina vavy				ODAM013
						Leaf	Heal the cracks/Cleanser/Tanned complexion/Beautifies/Color skin/Colors nails/Softens/Moisturizes/Nourishes/Brigthens/Add shine/Tightens/Light complexion/Anti aging	101	
Lythraceae	*Sonneratia alba* Sm	Manglier fleur	ND	M'honko ndziwi	Honko bé				ODAM049
						Leaf	Dermatoses	1	
Malvaceae	*Ceiba pentandra* (L.) Gaertn.	Kapokier	Silk-cotton tree, Kapok tree	M'pembafouma	Pembafuma				ODAM003
						Leaf	Cleanser/Nourishes/Softens/Elongates curl, Minimizes frizz/Stimulates hair growth/Cleanser/Detangles	18	
Malvaceae	*Cola nitida* (Vent.) Schott & Endl.	Colatier	Kola	Kola	Kola				ODAM053
						Fruit	Stimulates, Energyzing/Tones	3	
Malvaceae	*Hibiscus schizopetalus* (Dyer) Hook.f.	Lanterne japonaise	ND	ND	Sary kafe maféki				ODAM051
						Flower	Make up	1	
Malvaceae	*Sida rhombifolia* L.	Faux thé	Queensland hemp	Shifunga koli	ND				ODAM050
						Leaf	Cleanser/Shampoo/Antiseptic	3	
Moraceae	*Ficus sycomorus* L.	Le figuier sycomore	ND	Muhu mambe	Adabu				ODAM052
						Latex	Anti pimples	1	
Musaceae	*Musa* sp.	Bananier	Banana tree	Tchindri	Voudi ni outsi				ODAM055
						Leaf	Tightens (baby skin)	1	
Myristicaceae	*Myristica fragrans* Houtt.	Muscadier	Nutmeg	Kougoumanga	Kougoumanga				NC
						Seed	Swelling	1	
Myrtaceae	*Syzygium aromaticum* (L.) Merr. & L.M.Perry	Giroflier	Clove tree	Karafu	Karafo				NC
						Clove bud	Cleanser/Beautifies/Fragrance/Dental care/Stimulates hair growth/Softens	8	
Nyctaginaceae	*Bougainvillea spectabilis* Willd.	Bougainvillier	Great bougainvillea	ND	Telo myova				ODAM020
						Flower	Beautifies/Fragrance	9	
Oleaceae	*Jasminum nummulariifolium* Baker	Jasmin	ND	Anfu	Anfu				ODAM056
						Flower	Fragrance/Beautifies/Cleanser/skin care/Softens/Nourishes/Moisturizes/Post-depilatory care/Add shine/Lightens	191	
Orchidaceae	*Vanilla* sp.	Vanille	Vanilla	Lavany	Lavany				
						Fruit (Pod)	Fragrance/Moisturizes/Protect/Well-being	10	
Oxalidaceae	*Averrhoa bilimbi* L.	Bilimbi	Bilimbi	Uhaju	Madiro antanana				ODAM057
						Fruit	Anti-pimples	1	
Pandanaceae	*Pandanus maximus* Martelli	Grand vacoa	ND	M'lua n'dzishe	Droa				ODAM058
						Flower	Fragrance/Beautifies/Skin care/Cleanser/Softens/Nourishes/Moisturizes/Post-depilatory care/Add shine/Lightens	69	
Pedaliaceae	*Sesamum indicum* L.	Sésame	Sesame	Pwendzi	Antsiguini				NC
						Seed	Dermatoses and allergy/Pimples that appear on the skin/Moisturizes/Softens/Beautifies/Cleanser/Brightens/Add shine/Lightens/Nourishes/Anti aging/Firm up the skin	45	
Piperaceae	*Piper nigrum* L.	Poivrier noir	Black pepper	Vilivili	Vilivili				ODAM059
						Seed	Clean the vagina/Anti-itch	10	
Poaceae	*Chrysopogon zizanioides* (L.) Roberty	Vétiver, chiendent odorant	vetiver	Manu kantru	Kotuvera				ODAM060
						Root	Fragrance/Beautifies/Cleanser/Softens/Nourishes/Moisturizes/Skin care/Post-depilatory care/Add shine/Lightens/Soothes	44	
Polypodiaceae	*Phymatosorus scolopendria* (Burm.f.) Pic.Serm.	Scolopendre, Patte lézard	ND	M'hono béni	Kangadja sampa ravihi				NC
						Leaf	Moisturizes/Cleanser/Beautifies	3	
Pteridaceae	*Pityrogramma calomelanos* (L.) Link	Fougère d'argent	ND	ND	Kangadja orimbo vihavy				NC
						Leaf	Face makeup, during ceremonies and weddings	3	
Rosaceae	*Rosa* sp.	Rosier	White rose	Maouwa	Mauwa				ODAM061
						Flower	Fragrance/Beautifies/Skin care/Cleanser/Softens/Nourishes/Moisturizes/Refreshes/Revives/Softens/Lightens	69	
Rubiaceae	*Guettarda speciosa* L.	Bois cassant bord-de-mer	ND	Fu m'tsanga	Fu m'tsanga				ODAM011
						Flower	Fragrance	1	
						Piece of wood (stem)	Anti-pimples	9	
Rubiaceae	*Ixora cremixora* Drake	ND	ND	Mianga nianga	Sari mapwera marachi				NC
						Flower	Beautifies	2	
Rutaceae	*Citrus aurantium* L.	Oranger	Sweet orange	Mavoubara	Tsoha mami				NC
						Fruit	Deodorant/Fragrance	2	
Rutaceae	*Citrus medica* L.	Cédratier	Citron	Murundra kapu	Tsoha kapo				NC
						Leaf	Reduce bad odors	1	
Rutaceae	*Citrus* sp.	Citronnier	ND	Djimogné	Tsoha foyi				ODAM062
						Fruit (juice)	Deodorant/Antiperspirant/Heal the cracks/Beautifies/Color skin/Color nails/Cleanser/Stimulates/tones/Energyzing/Pimple on tongues causing small roses	59	
						Fruit (peel)	Deodorant	1	
Rutaceae	*Murraya paniculata* (L.) Jack	Oranger jasmin	orange jessamine	Fulagin	Anfololo				ODAM015
						Flower	Beautifies/Fragrance	10	
Sapindaceae	*Litchi chinensis* Sonn.	Litchi de Chine	Lychee	Litshi	Litshi				ODAM064
						Piece of wood (stem)	Sun protection	1	
Sapindaceae	*Sapindus saponaria* L.	Arbre à savon	Soap tree	Arita	Sabon kakazo				ODAM063
						Leaf	Relieves heat rash on the body that itches/	1	
						Seed	Relieves heat rash on the body that itches/Reduces irritation/Anti-pimples/Treats irritations and body rashes, such as chickenpox	3	
Sapotaceae	*Gambeya boiviniana* Pierre	Caïmitier de bovin	ND	ND	Famelho				NC
						Leaf	Toothache (mouthcare)	1	
Solanaceae	*Cestrum nocturnum* L.	Jasmin de nuit	Lady of the Night	Anfu ya huku	Sirya huku				ODAM011
						Flower	Beautifies/Fragrance	7	
Solanaceae	*Solanum melongena* L.	Aubergine	Eggplant	Bégani	Bégani				NC
						Fruit	Heal the cracks	1	
Solanaceae	*Solanum richardii* Dunal	ND	Eggpplant	ND	Sari anguivi				ODAM021
						Leaf	Toothache (mouthcare)	1	
Verbenaceae	*Lantana trifolia* L.	Lantanié trifolié	ND	M'bwasera	Sari fatsiki madani				NC
						Leaf	Tightens/Tones	2	
Verbenaceae	*Stachytarpheta urticifolia* (Salibs.) Sims.	Verveine bleu	ND	M'ri wagwegwe	Mshari				ODAM023
						Leaf	Intimate hygiene/Anti spot/Anti fungal	4	
Vitaceae	*Leea guineensis* G.Don	ND	ND	ND	Sadrakidraki				NC
						Root	Anti pimples/Accelerates wound healing/Anti fungal/Anti scabies	4	
Zingiberaceae	*Curcuma amada* Roxb	*ND*	*Mango ginger*	Siguizo manga	Sakéy manga				NC
						Rhizome	Skin care	1	
Zingiberaceae	*Curcuma longa* L.	Curcuma	Turmeric	Dzidzano	Tamotamo				NC
						Rhizome	Skin face/Skin face/Sun protection/Anti pimples/Cleanser/Beautifies/Moisturizes/Softens/Nourishes/Add shine/Sun protection/Beauty mask/Massage/Lightens/Tanned complexion/Well-being/Anti aging/Tightens	105	
Zingiberaceae	*Zingiber officinale* Roscoe	Gingembre	Ginger	Siguizo	Sakéy				NC
						Rhizome	Tightens	1	
Zingiberaceae	*Zingiber zerumbet* (L.) Sm.	Amome sauvage	Shampoo ginger	Singuizo masera	Sakéyi loulou				NC
						Flower	Shampoo/Cleanser	2	
ND	ND	"Bois de santal"	Sandalwood	Msindzano	Msindzano				NC
						Piece of wood (stem)	Cleanser/Beautifies/Fragrance/Sun protection/Anti aging/Anti pimples/Anti spot/Reduces irritation/Refreshes/Lightens/Softens/Beauty mask/Redness (baby)/Foundation/Tanned complexion	116	

Legend: ND for “not determined”, NC for “not collected”, plants not collected were identified based on their vernacular names by referring to “La flore illustrée de Mayotte” (Barthelat, 2019). Symbol “/” is used for “and”, and symbol “,” is used for “or”. * Coconut milk is obtained by expression of solid endosperm of coconut. **Coconut oil is obtained by boiling the coconut milk.

Regarding the most cited plant species for each of the six most reported cosmetic use, *Guettarda speciosa* rank first (5 informants) followed by *Persea americana* (4 informants) and *Sapindus saponaria* (4 informants) for reducing pimples. For moisturizing the skin, *Cocos nucifera* rank first (17 informants) followed by *Curcuma longa* (6 informants), *Chrysopogon zizanioides* (4 informants), *Jasminum nummulariaefolium* (4 informants) and *Ocimum* sp. (4 informants). For adding shine to the skin, *Cocos nucifera* rank first (5 informants) followed by *Curcuma longa* (3 informants). For nourishing the hair, *Cocos nucifera* rank first (14 informants) followed by *Ceiba pentandra* (2 informants) and *Ipomoea obscura* (2 participants). For beautifying, *Jasminum nummulariaefolium* rank first (19 informants) followed by *Lawsonia inermis* (16 informants) and *Cananga odorata* (14 informants). For perfuming, *Cananga odorata* rank first (16 informants) followed by *Jasminum nummulariaefolium* (14 informants) and *Ocimum* sp. (14 informants).

**Fig. 6:**
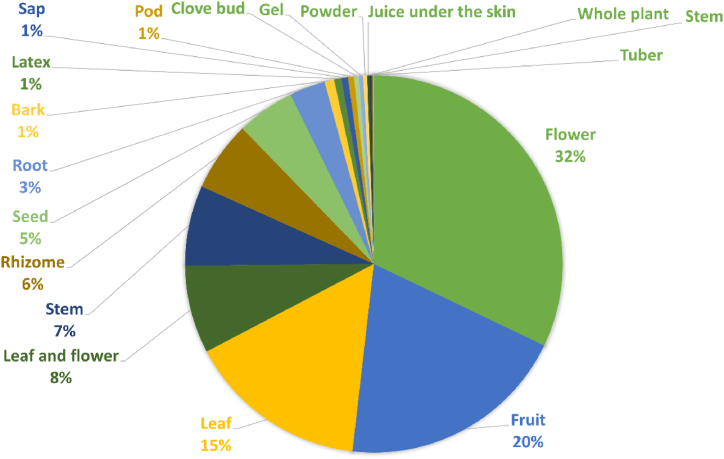
Frequency of citations of all part of plants used in cosmetic recipes.

In our study, four plant parts are widely used: flowers with 32 % of citations, fruit with 20 % of citations, leaves with 15 % of citations, and leaves and inflorescences with 8 % of citations (Fig. 6). The primary mode of administration is generally cutaneous, except for oral hygiene applications such as mouthwash, as well as the use of hairpins and necklaces.

Out of the 83 plant species recorded, five (i.e., *Commiphora arafy*, *Gambeya boiviniana*, *Ixora cremixora*, *Jasminum nummulariifolium*, *Kedrostis elongata*) are endemic to Comoros islands and Madagascar; and two plant species are endemic to Comoros islands only (i.e., *Croton mayottae*, *Pandanus maximus*).

### 3.5 Toxicity reported by informants

In our study, some plants have been reported to be toxic by the informants. The first one, *Youngia japonica*, was cited to cause burns and itching. Therefore, the informants recommend to test it on a small patch of skin before properly using it. Additionally, it may be botanically confused with another plant species namely *Launaea intybacea* (Jacq.) Beauverd, which is also consumed and used in traditional medicine. Also, *Mimosa pudica* and *Sapindus saponaria* have been cited as toxic because they both induce itching on the skin, with a more pronounced effect for *S. saponaria*.

## 4 Discussion

This study describes the cosmetic flora of Mayotte along with its uses. To the best of our knowledge, this field survey, focusing on traditional cosmetic products, is the second conducted in Mayotte, but it is the only one to solely focus on Mahoran cosmetopoeia. The other study was conducted by Saive in the Comoros, specifically in Mayotte [18]. In comparison to Saive's study, ours covered the entire mainland with slightly more respondents in the southern part of the island. Saive documented 34 plants used in cosmetics, while our study identified 83 plants. Our literature analysis reveals an almost nonexistent dedicated study on cosmetopoeia at the regional level.

Overall, this article presents the findings of a study based on 35 interviews, resulting in a list of 470 recipes that highlight the cosmetic use of plants, along with their associated claims. The number of ingredients per recipe ranges from 1 to 13, with a prevalence of mono-ingredient recipes. Out of the five cosmetics categories, hygiene, make-up, perfume, along with hair and nails, and dermatology were the most represented ones. The analysis of cosmetic claims reveals significant trends, emphasizing properties such as hydration and skin radiance as the most cited ones. As in sub-Saharan Africa, Mahoran people use skin depigmentation products to make skin lighter. Our study revealed that very few plants are used to achieve a fair and bronzed complexion. However, numerous claims revolve around the first, fourth, and fifth groups, namely dermatology, hair, and appendages, as well as toiletries.

Here, we will briefly delve into each of the most cited plant species and families, outlining their geographical distribution, botanical characteristics, ethnobotanical uses (especially as cosmetics), biological properties associated with their cosmetic uses, chemical composition, and toxicology (if available).

### 4.1 Cocos nucifera

#### 4.1.1 Geographical distribution, botanical characteristics

The coconut is believed to originate from the Indo-Malayan region, from where it spread across tropical regions. While its original habitat was limited to sandy coastlines, it has since adapted to various types of soil, ranging from pure sand to clays. Despite its non-invasiveness, the coconut's spread has mainly been facilitated by human migration, particularly beyond its native coastal habitats. There are various varieties of *C. nucifera*, distinguished by factors such as fruit size or pericarp color [[19], [20], [21], [22]].

*C. nucifera*, commonly known as coconut, typically consists of three distinct layers: the epicarp or exocarp, which is the outermost layer; the mesocarp, also known as the husk, which is the intermediate layer; and the endocarp, which is the hard outer layer. The coconut seed is located inside the fruit and is composed of both the tegument and the solid and liquid endosperm. The tegument is the thin brown layer that surrounds the solid endosperm, which is the white, fleshy, and oily pulp of the seed. Coconut oil is extracted from the tegument [23].

#### 4.1.2 Ethnobotanical uses

In our study, *Cocos nucifera* was predominantly used for skincare, moisturizing, nourishing, radiance, and sun protection purposes. Skincare claims were among the most frequent cosmetic claims reported for *C. nucifera*.

In Mayotte, the use of coconut (*C. nucifera*) fruit in cosmetics has already been reported, and its uses include smoothing and brightening properties [18]. In the neighboring Comoros islands, it has been reported that coconut, combined with sandalwood (*S. album*) and sesame seeds (*S. indicum*), is employed to lighten the skin and eliminate acne in the form of a beauty mask [24]. In Mauritius, coconut is part of a cosmetic product called *amlaroma* which contains the following blend: *Embelica officinalis* Gaertn, *Trigonella foenum* L., *Centella asiatica* (L.) Urb., *Eclipta alba* L., along with sesame oil, coconut oil, sunflower oil, and citronella oil. This mixture is used for hair growth stimulation, stress reduction, dandruff elimination, and prevention of hair loss [25]. Coconut oil is also used in Mauritius in combination with fenugreek seed as an anti-hair loss shampoo, hair conditioner and hair serum [26]. In Africa, the combination of coconut oil with *Trichilia emetica* Vahl oil has an emollient effect on the skin [27]. For example, in Nigeria coconut oil is also often used on new-born babies, basically for oiling the hair and to maintain body pigmentation [28]. In Senegal, *Trichilia emetica* Vahl oil is combined with coconut oil as a cosmetic to provide emollient and moisturizing effect [29]. In India, coconut milk combined with carrot juice is employed topically for anti-aging purposes. Coconut milk is also applied to the hair to combat hair loss. As for the oil, it is applied to the nails and lips for care [30]. In French Polynesia, coconut oil also plays a crucial role in the preparation of a traditional blend known as “pani,” akin to the French monoi. It possesses virtues such as soothing, softening, hydrating, and nourishing [6,31,32]. In Asia, the oil from mature coconut fruit is massaged into the head for soft and shiny hair [33].

The cosmetic claims reported in Mayotte are not specific to that region; they are also found in other regions regarding aspects such as emollient, nourishing, and caring for skin or hair. However, the beauty masks used in the Comoros Islands and prepared with coconut oil have not been reported elsewhere.

#### 4.1.3 Biological properties and chemical composition

In recent years, the oil derived from *C. nucifera* has been extensively utilized due to its numerous virtues, including benefits in skincare, haircare, stress relief, weight management, cholesterol level maintenance, immunomodulatory effects, cardiovascular applications, and more recently, in the context of Alzheimer's disease [34]. Coconut oil is utilized as a moisturizer, cleanser, and foaming agent. It is employed in the production of soap and shampoo [35,36]. In the cosmetic products from the industry, coconut comes in various forms, including coconut oil, coconut acid, hydrogenated coconut oil, and hydrogenated coconut acid, with over twenty cosmetic ingredients derived from coconut oil, such as fatty alcohols, fatty acids, esters, and salts. These include ammonium cocomonoglyceride sulfate, butylene glycol cocoate, caprylic/capric/coco glycerides, cocoglycerides, coconut alcohol, decyl cocoate, decyl esters of coconut oil, ethylhexyl cocoate, hydrogenated coco-glycerides, isodecyl cocoate, lauryl cocoate, magnesium cocoate, methyl cocoate, octyldodecyl cocoate, pentaerythrityl cocoate, potassium cocoate, hydrogenated potassium cocoate, sodium cocoate, sodium cocomonoglyceride sulfate, hydrogenated sodium cocoate, and tridecyl cocoate [35].

The composition of coconut oil is as follows: lauric acid (45 %–52 %), myristic acid (16 %–21 %), palmitic acid (7 %–10 %), caprylic acid (5 %–10 %), capric acid (4 %–8 %), stearic acid (2 %–4 %), caproic acid (0.5 %–1 %), and palmitoleic acid (in traces). It also contains unsaturated fatty acids: oleic acid (5 %–8 %), linoleic acid (1 %–3 %), and linolenic acid (up to 0.2 %) [34]. Lauric acid has been recognized for its antimicrobial activity [37].

In conclusion, *Cocos nucifera* is a well known plant species employed for cosmetic purposes all over the world. In Mayotte, beauty masks, specific from the region, are made from coconut oil and other ingredients. Biological properties and phytochemical compounds from coconut are already known, and justify its use as a cosmetic agent.

### 4.2 Jasminum sp.

#### 4.2.1 Geographical distribution, botanical characteristics

The genus *Jasminum* belongs to the Oleaceae family and is widely distributed worldwide. With over 2000 species spread globally, *Jasminum* find its origins in Eurasia, India, and Mediterranean regions and are found in native to tropical or warm temperate regions. In our survey, *Jasminum* has been the most frequently mentioned genus from the Oleaceae family. Different varieties of *Jasminum* exist in Mayotte, among which *J. nummulariifolium* is the only one reported as cosmetic agents. *J. nummulariifolium* is endemic to Madagascar and Comoros islands. The analysis of cosmetic claims reveals a lack of diversity, as all claims are concentrated in category five (hygiene, perfume and make-up), accounting for 93 %. The primary claims focus on embellishment and fragrance.

#### 4.2.2 Ethnobotanical uses

In Mayotte, Saive noted the uses of the same species as a perfume, smoothing agent, lightening agent, and for acne [18]. In the Comoros, Saive described the use of the same species (*J. nummulariifolium*) against acne [38]. Soidrou observed that this species is included in the preparation of *msindzano* in combination with *Sesamum indicum*, *tamtam* (local name), *Tamarindus indicus*, *loksi* (local name), *Myristica fragrans*, *Persea americana*, *Santalum album, Lawsonia inermis*, claiming to lighten the skin and eliminate acne. In the same publication, another preparation combining *J. nummulariifolium*, *Myristica fragrans* and *Santalum album* was reported for sun protection against UV rays and lightening the skin [24].

To the best of our knowledge, there is no available data on the use of J. *nummulariifolium* in Madagascar and Reunion island. However, other plant species from the *Jasminum* family have already been reported to be used elsewhere as cosmetics. For example, in Mauritius, *Jasminum officinale* L. is used for perfume and as a whitening agent [26]. In French Polynesia, *Jasminum grandiflorum* L. is used in monoi for the softness and the care of body and hair [31]. In India, tribal women from Kashmir Himalayas used the leaves of *J*. *officinale* for freshness [39]. In Sri-Lanka, *J*. *grandiflorum* plant extracted oil was reported to be used for scalp cooling and *Jasminum multiflorum* (Burm.f.) Andrews was reported to be used for skin care [40].

#### 4.2.3 Biological properties and chemical composition

Species like *J. grandiflorum, Jasminum sambac* (L.) Aiton*, Jasminum flexile* Vahl*,* and *Jasminum angustifolium* (L.) Willd. have traditionally been used for their antimicrobial, antidepressant, flavoring, and fragrance properties. Jasmine oil is extensively used in aromatherapy. The medicinal properties of *Jasminum* plants can be attributed to the presence of a wide range of bioactive compounds, including phenolic compounds, terpenoids, coumarins, glycosides, sterols, esters, and fatty acids. The antimicrobial, anti-acne, spasmolytic, and aromatherapeutic activities arise from the combined effect of essential oils [41]. Throughout history, the *Jasminum* genus has played a significant role in traditional medicine. For instance, *J. officinale* exhibits a diverse range of therapeutic properties, including depurative, antiseptic, and expectorant virtues. Similarly, *J. grandiflorum* is recommended for cough. Also, *J. sambac* has expectorant, analgesic, antiseptic, aphrodisiac, and antidepressant properties [[42], [43], [44]]. *Jasminum* like *J*. *grandiflorum* flower is used like absolute, concrete, for creams, emulsions, shampoos, skin care (dermatitis, whitening, anti-itch), and perfumes [45]. *J*. *sambac* can be used in cosmetics as an essential oil, absolute, or concrete in cosmetic products and perfumes. The flowers of *J*. *sambac* are known for their antioxidant, anti-aging, brightening, and antibacterial activities [46].

Overall, *J. nummulariifolium* flowers represent an interesting cosmetic agent due to its specific uses for sun protection, skin lightening and acne in Comoros islands. This plant species is also endemic to Comoros islands and Madagascar and thus represent a biological heritage for the region. To the best of our knowledge, no pharmacological and phytochemical data were found in the literature on this plant species highlighting the need for further research.

### 4.3 Ocimum spp.

#### 4.3.1 Geographical distribution, botanical characteristics

Species of *Ocimum* genus belong to the Lamiaceae family and encompass a variety of over 150 species distributed in temperate and tropical regions around the world. It is noteworthy that the highest number of species is found in Africa and India*. Ocimum* constitutes a group of aromatic and medicinal plants within a genus of both annual and perennial herbs, exhibiting a distinctive bushy growth pattern.

#### 4.3.2 Ethnobotanical uses

In our study, different species of *Ocimum* were reported to be used as cosmetic agents, and they were mainly used for hygiene, make-up, and perfume (group 5). Leaves from two varieties of *Ocimum* spp. are used for fragrance and as beautifying agents, while leaves from the species *O*. *gratissimum* is only used for vaginal disorders. In the literature, *Ocimum* species were already reported as cosmetic agents. For example, in Mayotte, *Ocimum canum* Sims is used in perfume [18]. In Comoros, *Ocimum americanum* L. *and O. gratissimum* are used for vaginal infection [38]. In Mauritius, *Ocimum tenuiflorum* L. leaves are used as an anti-pigmentation agent for face [26,47]. It is noteworthy that the use of *O. gratissimum* for intimate care is specific to the Comoros region, including Mayotte.

#### 4.3.3 Chemical composition

The commercially utilized parts of plant for essential oil extraction are primarily the leaves [48]. The chemical constituents from the leaves showed the presence of monoterpene hydrocarbons, oxygenated monoterpene, sesquiterpene hydrocarbons, oxygenated sesquiterpene, triterpene, flavonoids, and aromatic compounds. The compounds have been reported to exhibit antibacterial and antifungal, anti-inflammatory, antioxidant, antiviral, insecticidal and wound-healing properties [49].

The basil oil from the Indian Ocean is phytochemically different from other varieties of basil. This basil is cultivated, and its oil is produced in locations such as Reunion island, Madagascar, various parts of Africa, and occasionally in the Seychelles [50]. The presence of linalool/methyl cinnamate and linalool/methyl chavicol combination is observed in this basil. The European chemotype, originating from Italy, France, Bulgaria, Egypt, and South Africa, is distinguished by the high concentration in linalool and methyl chavicol. In contrast, the tropical chemotype, hailing from India, Pakistan, and Guatemala, is characterized by a notable presence of methyl cinnamate. The “La Reunion” chemotype, with roots in Thailand, Madagascar, and Vietnam, stands out for its elevated concentrations of methyl chavicol [51]. Basil or *Ocimum basilicum* L. is economically important due to the use of its essential oil in hygiene and cleaning products, perfumes, and cosmetics and as a local anesthetic and antiseptic [52].

In conclusion, *Ocimum* spp. are highly used as cosmetic agents in the Indian Ocean and elsewhere in the world. The bioactive compounds and associated biological activities are well known. Due to the presence of different cultivars and a lack of clear taxonomical identification, phylogenetic research is needed to differentiate between the different species used.

### 4.4 Curcuma longa

#### 4.4.1 Geographical distribution, botanical characteristics

*Curcuma longa* is native to India, and is now cultivated all over the world. *C. longa* consists of a rhizome, which is the main stem, accompanied by numerous offshoots or branches displaying a characteristic orange color [53].

#### 4.4.2 Ethnobotanical uses

In our survey, *Curcuma longa* was reported to be used for a variety of cosmetic purposes, and was mainly cited for moisturizing, nourishing, softening the skin and as an anti-aging (group 2); for providing shine, imparting a tan, and illuminating (group 3); and for cleansing and beautifying the skin (group 5). In the literature, Saive already reported the use of *C. longa* in Mayotte for preparing *msindzano* for redness, smoothing, and lightening [18]. In Comoros, Soidrou observed the uses of *C. longa* combined with *Tamarindus indica* L.*, chikélé* (local name)*, Chrysopogon zizanioides, loksi, Curcuma longa, Santalum album* L. against heat, as a sun block and for skin lightening. In the same publication, other recipes include *Curcuma longa* in combination with other plants for lightening the skin, against heat, and against UV radiation [24]. In Madagascar, it is used as an antimicrobial, anti-inflammatory, antibiotic and antioxidant agent [54]. In Mauritius, *C. longa* is used for face cleaning, and as a face mask, a moisturizer and a whitening agent [26].

In French Polynesia, *C. longa* is used in the preparation of “paku” a Marquesan cosmetic. Two types of *paku* are identified: the first is dedicated to the care and beauty of hair and skin; the second is mostly dedicated to children and newborns, as a preventive treatment, in order to care cradle cap, to avoid bad smells and to limit vaginal discharges [31]. In India, it is used during Hindu ceremonies where brides rub their bodies with a turmeric paste in order to have a radiant complexion. Washing with turmeric will brighten the skin and reduce hair growth. The extract of the plant is also used in hair preparations as an anti-dandruff [55]. The oil has extensive application in flavor, perfumery, cosmetic and food products [56].

#### 4.4.3 Biological properties and chemical composition

The orange color of the rhizome is attributed to the curcuminoid pigments, namely curcumin, demethoxycurcumin, bisdemethoxycurcumin, and cyclocurcumin [57]. These compounds possess various biological effects especially anti-inflammatory effects.

Overall, *Curcuma longa* is used all over the world for its cosmetic and medicinal applications. Its chemical composition and biological properties are widely known, and justify its use as a cosmetic agent in Mayotte. Our study is the first to report new cosmetic claims (i.e., sun protection, anti-aging, anti-pimples) for *Curcuma longa* in Mayotte.

### 4.5 Lawsonia inermis

#### 4.5.1 Geographical distribution, botanical characteristics

*Lawsonia inermis*, commonly known as henna tree, is a small shrub-like tree, 2–6 m high, with branchlets tipped with spines. Its leaves are smooth, arranged oppositely, nearly sessile, and elliptical to broadly lanceolate in shape, showing depressed veins prominently on the upper surface. Henna flowers consist of four sepals surrounding a 2 mm calyx tube, with white or red stamens arranged in pairs along the edge of the calyx tube, and petals that are ovate and somewhat crumpled. The ovary is divided into four cells, with an upright style. The tree bears small, brown fruits containing 32 to 49 angular seeds [58]. *L. inermis* is native to East Africa, Arabian Peninsula, Middle East and South Asia.

#### 4.5.2 Ethnobotanical uses

In our survey, all the cosmetic claims listed are distributed among four groups, with 46 % in group 5, 29 % in group 2, 13 % in group 3, and 12 % in group 4. The most commonly mentioned claims are: nourishing, gives a radiant and glowing complexion, brightens the skin and provides a sun-kissed tone, addresses cracks and chapping, beautifies the skin, and cleanses it. In the literature, Saive reported that the plant is used for redness, lightening, smoothing, headache, and in perfume [18]. In Comoros, Soidrou observed that the plant is used combined with *mté* (*Santalum album*) against heat and acne. In the same study, another recipe combines *Sesamum indicum*, *tamtam*, *Jasminium nummulariifolium*, *Tamarindus indicus*, *loksi*, *Myristica fragrans*, *Persea americana*, *Santalum album*, and *Lawsonia inermis* for lightening skin and as a sun block agent. Other recipes including *Lawsonia inermis* (in combination with other plants) are also employed for similar cosmetics uses such as skin lightening, sun blocking and against heat [24]. To the best of our knowledge, no data are available regarding the cosmetic use of *L. inermis* in Madagascar, Reunion, Seychelles. In Mauritius, it is used for hair coloring. In the Middle East and north Africa, henna is used for body art [59]. In Morocco, it is used during wedding and burial ceremonies [60]. Henna has been used to adorn the bodies of young women during social celebrations and festive occasions, with henna designs applied to their nails, palms, and soles in India. Especially during weddings, the tradition of “Henna Night” is celebrated by most ethnic groups in regions where henna naturally grows, including Jews, Muslims, Hindus, and Christians, among others. In all these cultures, henna is an essential element of wedding ceremonies, used to adorn the bride and the groom [61,62].

#### 4.5.3 Chemical composition

Nearly 70 phenolic compounds have been identified in different parts of the plant. Naphthaquinones, including the dyeing agent lawsone, have been associated with numerous pharmacological activities. Lawsone is a red-orange dye (2-hydroxy-1,4-naphthoquinone), also known as hennotannic acid. The terpene β-ionone is primarily responsible for the strong aroma of the essential oil extracted from the flowers. Alongside other volatile terpenes, certain non-volatile terpenoids, a singular sterol, two alkaloids, and two dioxin derivatives have also been isolated from the plant [58,63]. *Lawsonia inermis* is utilized in the cosmetic industry due to the abundant presence of active ingredients such as lawsone. This compound is commonly employed in the field of hair dyeing, nail care, hand treatments, and textile coloring [64].

In conclusion, *Lawsonia inermis* is a plant known worldwide for its cosmetic applications, and already studied for its biological effects. In Mayotte, *Lawsonia inermis* leaves are not only used for beautifying but also for healing (feet and heels cracks especially). Henne can be considered as an important cultural plant for Mayotte as it is used by Muslims people (representing 95 % of the total population of Mayotte) for religious events.

### 4.6 Cananga odorata

#### 4.6.1 Geographical distribution, botanical characteristics

*Cananga odorata*, commonly known as ylang-ylang, is a medium-sized evergreen tree, reaching heights of 10–20 m. The tree typically features a singular main trunk and an irregularly spreading crown composed of drooping branches and twigs arranged in two rows bearing leaves. It is easily identifiable by its uniquely shaped, highly fragrant flowers, which range in color from yellow to greenish-yellow, and its characteristic aggregate fruit consisting of clustered green or black berries, numbering between 8 and 15 [65]. This tree is native to Southeast Asia, including the Philippines, Malaysia, and Indonesia. It is renowned for its fragrant flowers that are extensively exploited for their essential oil, a crucial raw material in the fragrance industry. In the Comoros islands (including Mayotte), the tree was introduced during the 20th century by French people in order to produce essential oils. To date, the Comoros islands are considered to be the largest producer of essential oils in the world [9].

#### 4.6.2 Ethnobotanical uses

In our study, the recorded cosmetic claims for *C. odorata* are predominantly found in group 5 (86 %), with the main ones being embellishment and fragrance. In Mayotte, Saive already reported that the plant is used in perfume. It is also used in perfume in Comoros and Madagascar [9]. To the best of our knowledge, no data are available for Reunion Island, Mauritius, Seychelles, and Africa. In French Polynesia, *C. odorata* is used in Marquesan cosmetics in perfume and for hydration [31].

#### 4.6.3 Biological properties and chemical composition

The essential oils, obtained through steam distillation from flowers, find applications primarily in the cosmetic industry, with some uses in the food industry as well. Indeed, ylang-ylang oil can be found in various cosmetic and households products such as massage oils, moisturizing creams, perfumes, and even scented candles [65,66]. Traditionally*, C. odorata* has been employed to address conditions such as malaria, stomach ailments, asthma, gout, and rheumatism. A total of 25 components were identified in the essential oil, with major compounds including *trans*-caryophyllene, ocimene, (E,E)-α-farnesene, phenylmethyl ester, farnesyl acetone, t-muurolol, farnesol, β-elemene, α-cadinol, copaene, benzyl benzoate, and *trans*-farnesol. The essential oil is notably rich in sesquiterpenoids, encompassing sesquiterpene hydrocarbons and monoterpene hydrocarbons, along with various oxygenated compounds [[67], [68], [69]].

Overall, *Cananga odorata* is culturally and economically important in the Comoros islands as it is widely cultivated for the cosmetic and fragrance industry. In Mayotte, the production is lower than in other Comoros islands due to higher labor cost, but the tree still represents an emblematic plant for this island. The use of fragrance obtained from its flower is one of the most cited cosmetic uses by Mahoran people, and further work are needed to revitalize the ylang-ylang cosmetic industry in Mayotte.

### 4.7 Pandanus maximus

#### 4.7.1 Geographical distribution, botanical characteristics

The Pandanaceae family includes several genera, among which are *Pandanus, Freycinetia, Sararanga*, and *Martellidendron* [70,71]. The Pandanaceae family is found across the paleotropics, spanning from West Africa to the islands of the eastern Pacific. Except for *Martellidendron*, which is native to Madagascar and the Seychelles, the genera share common distribution ranges, primarily concentrated in Indonesia, Borneo, the Philippines, and New Guinea [72]. *Pandanus maximus* is an endemic tree of Mayotte which grows in sandy areas. It is a dioecious tree, with arched trunks, little branched, stems marked with helical scars from the abscission of the leaves and bearing protuberances, large aerial weeds generally grouped towards the base of the trunk, and leaves in apical helices.

#### 4.7.2 Ethnobotanical uses

In our study, *Pandanus maximus*, known for its highly valued fragrant flowers, is typically used either alone or in combination with two varieties of *Ocimum* locally known as *mkadi*. These flowers are often placed on safety pins or incorporated into necklaces along with other fragrant plants. The essential oil derived from the male flowers of *Pandanus odorifer* (Forssk.) is extensively employed in aromatherapy and cosmetic applications. The predominant component in the essential oil is phenylethyl methyl ether, which imparts the distinctive fragrance that defines the oil [73]. To the best of our knowledge, there is no pharmacological and phytochemical information available on *Pandanus maximus*. Nevertheless, it should be noted that this species is endemic to the Comoros, like *Pandanus mayottensis*.

*Pandanus maximus* flowers have been highly cited in our study, especially for fragrance. Because *Pandanus maximus* is endemic to Comoros islands, and few biological and chemical studies have been performed so far on this species, further research should be carried out to identify interesting compounds for the cosmetic industry and so provide an economic value to this species for Mahoran people.

Our study offers a detailed overview of cosmetic practices in Mahoran culture, providing valuable insights for future research in this field. However, there are some limitations to our study that should be noted. First, this study focused on tradipractitioners only (called *fundi*), and so we believe expanding the sample to the entire population would yield quantitative data that are more representative of current knowledge. This approach could also unveil emerging trends in cosmetic practices among the broader population. Second, *msindzano*, a beauty mask used all over the islands, could not always be clearly described from a botanical perspective. Indeed, *msindzano* is composed of different plant species imported as piece of woods in Mayotte and so botanically unidentifiable, thus representing a challenge for ethnobotanists.

## 5 Conclusion

In conclusion, this ethnobotanical study focusing on the cosmetic flora of Mayotte aimed to preserve knowledge and provide a foundation for local farmers interested in entering the cosmetics industry. To the best of our knowledge, this field survey is the first to solely focus on Mayotte's cosmetopoeia.

Based on our results, the Mahorese cosmetopoeia include 83 plants, some of which are well-known as cosmetic agents, such as *Cananga odorata*, *Cocos nucifera,* and *Plumeria* sp. Other species (e.g., *Jasminum nummulariifolium*, *Pandanus maximus*) highly mentioned in our survey are less known and should be further studied. It is noteworthy that perfumed plants emerge as a primary feature of Mahorese cosmetopoeia.

Undertaking such ethnobotanical investigations is of great importance, particularly as the transmission of traditional practices diminishes amidst the prevalence of readily available Western products. Given the resurging interest in natural products, it becomes imperative to expand ethnobotanical inquiries concerning cosmetopoeia, and document the diverse cultural heritage prevalent in overseas territories. This endeavor serves both preservation and promotional purposes.

## CRediT authorship contribution statement

**Oumaynou Daroueche:** Data curation, Formal analysis, Investigation, Writing – original draft, Writing – review & editing. **Abassi Dimassi:** Investigation, Writing – review & editing. **Cédric Bertrand:** Conceptualization, Funding acquisition, Writing – review & editing. **François Chassagne:** Conceptualization, Formal analysis, Writing – review & editing.

## Declaration of competing interest

The authors declare that they have no known competing financial interests or personal relationships that could have appeared to influence the work reported in this paper.
