# Supplementary material for: Exploring traditional cosmetic flora from Comoros islands: An ethnobotanical survey in Mayotte
Source: Heliyon. 2024 Jul 31;10(15):e35322. doi: 10.1016/j.heliyon.2024.e35322 (PMC11334832; doi:10.1016/j.heliyon.2024.e35322)
Supplement: Multimedia component 1 [file mmc1.docx]

**Exploring traditional cosmetic flora from Comoros islands: an ethnobotanical survey in Mayotte**

DAROUECHE Oumaynou^1,2^, BERTRAND Cédric^1,2,3^, CHASSAGNE François^4^*

^1^ UAR 3278 CRIOBE, EPHE-UPVD-CNRS, LabEx-CORAIL, Université de Perpignan, 52 Avenue Paul Alduy, 66860 Perpignan Cedex, France

^2^ Pôle d’Excellence Rurale, Pôle PI2M, RN 2 97670 Coconi, Mayotte

^3^ S.A.S. AKINAO, 52 Avenue Paul Alduy, 66860 Perpignan Cedex, France

^4^ UMR 152 PharmaDev, Université Paul Sabatier, Institut de Recherche pour le Développement (IRD), Toulouse, France.

*Corresponding author :

François Chassagne

Université Paul Sabatier

Faculté de Pharmacie

35 Chemin des Maraîchers

31062 Cedex 09

Toulouse

FRANCE

[francois.chassagne@ird.fr](mailto:francois.chassagne@ird.fr)

**Suppl. Mat. Figure1**: Distribution of URs of plants from different families across the five categories of cosmetic claims. a: *Arecaceae*, b: *Oleaceae*, c: *Lamiaceae*, d: *Zingiberaceae*, e: *Lythraceae,* f: *Annonaceae*, g: *Fabaceae*, h: *Pandanaceae*, i: *Rosaceae*, j: *Rutaceae*, k: *Apocynaceae*, l: *Poaceae*, m: “Msindzano”, n: “Tamotamo hazo”.

**
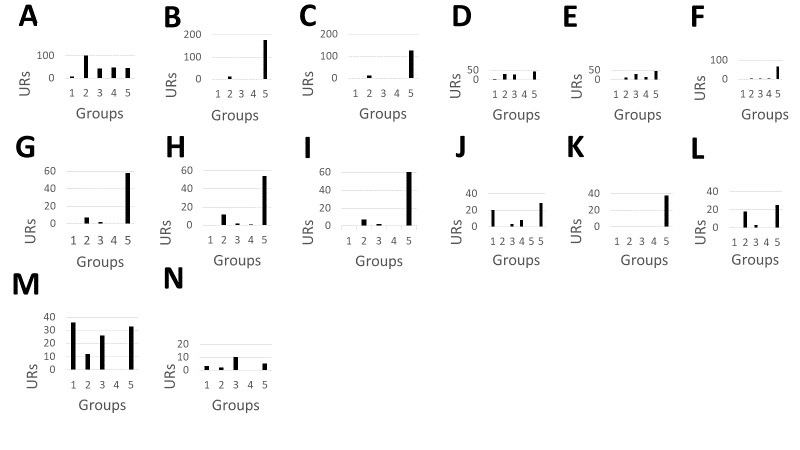
**

**Suppl. Mat. Table1**: List of plant species, preparations methods and methods of administration used in recipes cited in our survey

| **Familly** | **Scientific name** | **Vernacular name (french)** | **English name** | **Vernacular name (mahoran)** | **Vernacular name (kibushi)** | **Part of plant used** | **Preparation method** | **Administration method** | **Allegation** | **Group of claim** | **Number of informants** | **URs** | **Herbarium number** |
| --- | --- | --- | --- | --- | --- | --- | --- | --- | --- | --- | --- | --- | --- |
| *Acanthaceae* | *Barleria lupulina* Lindl. | Herbe tac-tac | ND | Mshari | Mamy lahy |  |  |  |  |  |  |  | ODAM019 |
|  |  |  |  |  |  | Leaf | Crush the leaves until the juice is extracted. | Tasks | Anti-blemish | 3 | 2 | 1 |  |
| *Aloeaceae* | *Aloe vera* (L.) Burm.f. | Aloès | Aloe | Shiizi ya mlili | Sakouhakinkigni |  |  |  |  |  |  |  | ODAM018 |
|  |  |  |  |  |  | Gel | Mix the gel with crushed leaves of *Indigofera tinctoria* | Wound or cut | Promotes healing | 1 | 1 | 1 |  |
|  |  |  |  |  |  |  | Crush the rhizome of *Curcuma longa*, extract the gel from *Aloe vera*, and cook the milk of *Cocos nucifera* to obtain the oil. Mix all the ingredients with "zoukouba" (Mix of plant perfume) | Massage | Massage | 1 | 12 | 1 |  |
|  |  |  |  |  |  |  |  | Massage | Cleanser | 5 | 12 | 1 |  |
|  |  |  |  |  |  |  | Mix the gel with water | Drink | Beautifies | 5 | 12 | 1 |  |
|  |  |  |  |  |  |  |  | Apply on the face, massage gently, rub a bit, and rinse | Face care | 2 | 16 | 1 |  |
|  |  |  |  |  |  |  |  | Apply on the hair, massage gently, rub a bit, and rinse | Shampoo | 4 | 16 | 1 |  |
|  |  |  |  |  |  |  |  | Apply on the hair, massage gently, rub a bit, and rinse | Smoothens | 4 | 16 | 1 |  |
|  |  |  |  |  |  |  | Mix gel of *Aloe vera* and *Cocos nucifera* oil | Body / skin | Skin care | 2 | 18 | 1 |  |
|  |  |  |  |  |  |  |  | Body / skin | Refreshes | 5 | 32 | 1 |  |
|  |  |  |  |  |  |  |  | Body / skin | Well-being | 5 | 32 | 1 |  |
|  |  |  |  |  |  |  |  | Body / skin | Preserves youth | 2 | 32 | 1 |  |
|  |  |  |  |  |  |  |  | Body / skin | Firms | 2 | 32 | 1 |  |
|  |  |  |  |  |  | Leaf | Decoction | Drink | Promotes hair growth | 4 | 20 | 1 |  |
|  |  |  |  |  |  |  |  | Hair | Promotes hair growth | 4 | 20 | 1 |  |
|  |  |  |  |  |  |  |  | Drink | Hydrates (face) | 2 | 20 | 1 |  |
|  |  |  |  |  |  |  |  | Face | Hydrates (face) | 2 | 20 | 1 |  |
|  |  |  |  |  |  |  |  | Drink | Moisturizes the Body / skin / skin | 2 | 20 | 1 |  |
|  |  |  |  |  |  |  |  | Body / skin | Moisturizes the Body / skin / skin | 2 | 20 | 1 |  |
| *Amaranthaceae* | *Achyranthes aspera* L. | Herbe d'Eugène | ND | Tsoho | Tsoho |  |  |  |  |  |  |  | ODAM030 |
|  |  |  |  |  |  | Leaf | Crush the leaves | Bites | Soothes | 1 | 31 | 1 |  |
|  |  |  |  |  |  |  |  | Wound | Heal | 1 | 31 | 1 |  |
| *Amaryllidaceae* | *Allium schoenoprasum* L. | Oignon vert | Green onion | Shourougou ya mani | Dugulu basoili |  |  |  |  |  |  |  | NC |
|  |  |  |  |  |  | Leaf | Crush the leaves of chives or green onion (*Allium schoenoprasum* or garlic *Allium spp*.) and the seeds of *Piper nigrum* until obtaining a puree | Apply to the vagina | Cleanser | 5 | 18 | 2 |  |
|  |  |  |  |  |  |  |  | Apply to the vagina | Anti-itch | 5 | 18 | 1 |  |
| *Amaryllidaceae* | *Allium* sp. | Ail | Garlic | Shourougou voushé | Dugulu layii |  |  |  |  |  |  |  | NC |
|  |  |  |  |  |  | Leaf |  | Apply to the vagina | Cleanser | 5 | 18 | 1 |  |
|  |  |  |  |  |  |  |  | Apply to the vagina | Anti-itch | 5 | 18 | 1 |  |
| *Anacardiaceae* | *Anacardium occidentale* L. | Anacardier | Cashew | Mabibo | ND |  |  |  |  |  |  |  | ODAM031 |
|  |  |  |  |  |  | Bark | Decoction | Mouthwash | Toothaches | 5 | 2 | 1 |  |
|  |  |  |  |  |  | Bark and leaf | A fairly concentrated decoction of the bark and leaves | Mouthwash | Toothaches | 5 | 31 | 1 |  |
|  |  |  |  |  |  |  |  | Mouthwash | Gingivitis | 1 | 31 | 1 |  |
| *Annonaceae* | *Cananga odorata* (Lam.) Hook.f. & Thomson | Canang odorant | Ylang-ylang | Langilang | Langilang |  |  |  |  |  |  |  | ODAM025 |
|  |  |  |  |  |  | Flower | No preparation required | Arrange on the bed | Fragrance | 5 | 1,7,11,13,26,32,33 | 7 |  |
|  |  |  |  |  |  |  | No preparation required | Arrange on the bed a mixture of leaves and inflorescences of *Ocimum* sp., the flowers of *Pandanus maximus*, *Jasminum nummulariaefolium*, and *Cananga odorata*. | Fragrance | 5 | 23 | 1 |  |
|  |  |  |  |  |  |  |  |  | Beautifies | 5 | 23 | 1 |  |
|  |  |  |  |  |  |  | Pierce the flowers using a needle and fill a thread with rice grains. Tie it all together. Used for making a necklace | Neck | Beautifies | 5 | 2,3 | 2 |  |
|  |  |  |  |  |  |  | Pierce the flowers using a safety pin | Neck | Beautifies | 5 | 2,17 | 2 |  |
|  |  |  |  |  |  |  |  | Hair | Beautifies | 5 | 2,3,17,33,21 | 5 |  |
|  |  |  |  |  |  |  |  | Hair | Fragrance | 5 | 33 | 1 |  |
|  |  |  |  |  |  |  |  | Clothing | Beautifies | 5 | 33 | 2 |  |
|  |  |  |  |  |  |  |  | Clothing | Fragrance | 5 | 33 | 1 |  |
|  |  |  |  |  |  |  | No preparation required | Arrange on the bed a mixture of flowers of *Cananga odorata* and *Jasminum nummulariaefolium* | Fragrance | 5 | 11 | 1 |  |
|  |  |  |  |  |  |  | Create a necklace with a mixture of flowers from *Jasminum nummulariaefolium, Plumeria alba, Cananga odorata, Bougainvillea spectabilis*, and leaves of *Pogostemon cablin*, as well as leaves and inflorescences of *Ocimum spp* | Place on the neck | Beautifies | 5 | 11 | 1 |  |
|  |  |  |  |  |  |  | Create a necklace with a mixture of flowers from *Jasminum nummulariaefolium, Plumeria alba, Cananga odorata, Bougainvillea spectabilis*, and leaves of *Pogostemon cablin*, as well as leaves and inflorescences of *Ocimum spp* | Place on the neck | Fragrance | 5 | 11 | 1 |  |
|  |  |  |  |  |  |  | Create a blend with the flowers of *Jasminum nummulariaefolium* and *Cananga odorata*, and place them on safety pins | Place on hair/clothing | Beautifies / fragrance | 5 | 14,16 | 3 |  |
|  |  |  |  |  |  |  | Mix the flowers of *Jasminum nummulariaefolium* and *Cananga odorata*, and place them on a necklace | Pace on neck | Beautifies / fragrance | 5 | 14,16 | 3 |  |
|  |  |  |  |  |  |  | ND | Body / skin | Perfume oil | 5 | 26 | 1 |  |
|  |  |  |  |  |  |  | The flowers are cooked in a pot to extract the oil | Body / skin | Fragrance | 5 | 12 | 1 |  |
|  |  |  |  |  |  |  |  | Body / skin | Massage | 5 | 12 | 1 |  |
|  |  |  |  |  |  |  | Combine all the following plants: *Chrysopogon zizanoides* root, leaves and inflorescences of *Ocimum sp.*, leaves of *Ayapana triplinervis*, flowers of *Jasminum nummulariaefolium*, *Pandanus maximus, Cananga odorata*; all sourced from safety pins. Infuse them in *Cocos nucifera* oil and let it macerate in the oil. (This is the matsha mahitri, matsha manu kantru). | Body / skin | Fragrance | 5 | 5 | 1 |  |
|  |  |  |  |  |  |  |  | Body / skin | Softens | 2 | 5 | 1 |  |
|  |  |  |  |  |  |  |  | Body / skin | Post-depilatory care | 4 | 5 | 1 |  |
|  |  |  |  |  |  |  | Mix the leaves and inflorescences of *Ocimum sp.* with the flowers of *Pandanus maximus*, *Rosa alba, Jasminum nummulariaefolium, Canaga odorata*, and roots of *Chrysopogon zizanoïdes*. Incorporate everything into *Cocos nucifera* oil and let it macerate. | Body / skin | Beautifies | 5 | 4 | 2 |  |
|  |  |  |  |  |  |  | Mix the leaves and inflorescences of *Ocimum sp.* with the flowers of *Pandanus maximus*, *Rosa alba, Jasminum nummulariaefolium, Canaga odorata*, and roots of *Chrysopogon zizanoïdes*. Incorporate everything into *Cocos nucifera* oil and let it macerate. | Body / skin | Fragrance | 5 | 4 | 2 |  |
|  |  |  |  |  |  |  | Mix the dried flowers of *Pandanus maximus, Jasminum nummulariaefolium, Cananga odorata*, the dried leaves of *Pogostemon cablin, Ayapana triplinervis*, the dried leaves and inflorescences of *Ocimum sp.*, and the root of *Chrysopogon zizanoides*. | Preparation for making scented oil | Fragrance | 5 | 11 | 1 |  |
|  |  |  |  |  |  |  | Mix the flowers of Vachellia farnesiana and Cananga odorata, and place them on a safety pin | Hair | Beautifies | 5 | 17 | 1 |  |
|  |  |  |  |  |  |  | Mix the flowers of Vachellia farnesiana and Cananga odorata and put them on a necklace | Neck | Beautifies | 5 | 17 | 1 |  |
|  |  |  |  |  |  |  | Water maceration and refrigerate | Face evening and sunrise | Refreshes | 5 | 14 | 1 |  |
|  |  |  |  |  |  |  |  | Face evening and sunrise | Well-being | 5 | 14 | 1 |  |
|  |  |  |  |  |  |  |  | Face evening and sunrise | Preserves youth | 2 | 14 | 1 |  |
|  |  |  |  |  |  |  | Infuse the dried plants, namely: Flowers of *Jasminum nummulariaefolium*, *Pandanus maximus, Plumeria alba, Cananga odorata, Rosa alba*, roots of *Chrysopogon zizanoides*, leaves of *Pogostemon cablin, Ayapana triplinervis*, and leaves and inflorescence of *Ocimum spp*., in a decoction of *Cocos nucifera* oil | Body/skin | Fragrance | 5 | 10 | 1 |  |
|  |  |  |  |  |  |  | Place a mixture of *Cananga odorata* flower, leaves and inflorescences of *Ocimum spp*. and *Jasminum nummulariaefolium* on a necklace | Neck | Fragrance | 5 | 32 | 1 |  |
|  |  |  |  |  |  |  |  | Neck | Beautifies | 5 | 32 | 1 |  |
|  |  |  |  |  |  |  |  | Clothing | Fragrance | 5 | 32 | 1 |  |
|  |  |  |  |  |  |  |  | Clothing | Beautifies | 5 | 32 | 1 |  |
|  |  |  |  |  |  |  | Adorn a necklace with a blend of *Cananga odorata* and J*asminum nummulariaefolium* flowers | Neck | Beautifies | 5 | 26 | 1 |  |
|  |  |  |  |  |  |  |  | Hair | Beautifies | 5 | 9,9,11,26 | 4 |  |
|  |  |  |  |  |  |  |  | Hair | Fragrance | 5 | 9,11 | 2 |  |
|  |  |  |  |  |  |  |  | Clothing | Beautifies | 5 | 11,26 | 2 |  |
|  |  |  |  |  |  |  |  | Clothing | Fragrance | 5 | 11 | 1 |  |
|  |  |  |  |  |  |  | Place a mixture of leaves and inflorescences of Ocimum spp., flowers of Pandanus maximus, Jasminum nummulariaefolium, and Cananga odorata on the kilabou | Hair | Beautifies | 5 | 23 | 1 |  |
|  |  |  |  |  |  |  | Place a mixture of leaves and inflorescences of Ocimum spp., flowers of Pandanus maximus, Jasminum nummulariaefolium, and Cananga odorata on the kilabou | Hair | Fragrance | 5 | 23 | 1 |  |
|  |  |  |  |  |  |  | Place on the necklaces | Neck | Beautifies | 5 | 33 | 1 |  |
|  |  |  |  |  |  |  |  | Neck | Fragrance | 5 | 21,33 | 2 |  |
|  |  |  |  |  |  |  | Take a mixture of plants obtained from weddings or create your own blend based on plant availability (*Chrysopogon zizanoides* root, *Ayapana triplinervis* leaves, leaves and inflorescences of *Ocimum spp.*, *Jasminum nummulariaefolium* flowers, *Vachellia farnesiana, Rosa alba*, Koukoumba, P*lumeria alba, Cananga odorata, Pandanus maximus*). Once dried, crush and sort them by size. Prepare coconut oil by grating the coconuts, extracting the milk, letting it sit overnight, removing the cream on top, and then cooking it in a large pot over a wood fire. Once the oil is ready, add the flower mixture (zoukouba) when the temperature has slightly decreased and add Pompeia(R) when the oil is no longer very hot. Let the mixture macerate. The longer the maceration, the more intense the fragrance will be. | Body / skin | Nourishes | 2 | 6 | 1 |  |
|  |  |  |  |  |  |  |  |  | Brightens | 3 | 6 | 1 |  |
|  |  |  |  |  |  |  |  |  | Softens | 2 | 6 | 1 |  |
|  |  |  |  |  |  |  |  |  | Fragrance | 5 | 6 | 1 |  |
|  |  |  |  |  |  |  |  |  | Brightens | 3 | 6 | 1 |  |
|  |  |  |  |  |  |  |  |  | Hydrates | 2 | 6 | 1 |  |
|  |  |  |  |  |  |  | ND | ND | Essential oil | 5 | 9 | 1 |  |
| *Apocynaceae* | *Carissa spinarum* L. | Bois amer | ND | M'djanfari | Taola na omby |  |  |  |  |  |  |  | NC |
|  |  |  |  |  |  | Piece of wood | Scrape the piece of wood on the dead coral stone with a little water until obtaining a smooth paste | Face | Beauty mask | 2 | 4 | 1 |  |
|  |  |  |  |  |  |  |  |  | Sun protection | 3 | 27 | 1 |  |
| *Apocynaceae* | *Plumeria* sp L. | Frangipanier | Nosegay Tree | ND | Ngaya bé |  |  |  |  |  |  |  | ODAM032; ODAM033 |
|  |  |  |  |  |  | Flower | Pierce the flowers using a needle and thread them through a rice sack thread | Neck | Beautifies | 5 | 2,3 | 2 |  |
|  |  |  |  |  |  |  | Pierce the flowers using a safety pin and stack them on it | Hair | Beautifies | 5 | 2,3,4 | 3 |  |
|  |  |  |  |  |  |  |  | Clothing | Beautifies | 5 | 2 | 1 |  |
|  |  |  |  |  |  |  | No preparation required | Arrange on the bed | Fragrance | 5 | 4 | 1 |  |
|  |  |  |  |  |  |  | String the flowers of *Jasminum nummulariaefolium* and *Plumeria alba* onto a necklace | Neck | Beautifies | 5 | 5 | 1 |  |
|  |  |  |  |  |  |  |  |  | Fragrance | 5 | 5 | 1 |  |
|  |  |  |  |  |  |  | String the flowers of *Jasminum nummulariaefolium* and *Plumeria alba* onto a "toka" | Neck | Beautifies | 5 | 5 | 1 |  |
|  |  |  |  |  |  |  |  |  | Fragrance | 5 | 5 | 1 |  |
|  |  |  |  |  |  |  | String the flowers of *Jasminum nummulariaefolium* and *Plumeria alba* onto a "kilabou" | Hair | Beautifies | 5 | 5 | 1 |  |
|  |  |  |  |  |  |  |  |  | Fragrance | 5 | 5 | 1 |  |
|  |  |  |  |  |  |  | String the flowers of *Jasminum nummulariaefolium*, *Plumeria alba,* and *Bougainvillea spectabilis* onto a necklace | Neck | Beautifies | 5 | 5 | 1 |  |
|  |  |  |  |  |  |  |  |  | Fragrance | 5 | 5 | 1 |  |
|  |  |  |  |  |  |  | Take a mixture of plants obtained from weddings or create your own blend based on plant availability (*Chrysopogon zizanoides* root, *Ayapana triplinervis* leaves, leaves and inflorescences of *Ocimum spp.*, *Jasminum nummulariaefolium* flowers, *Vachellia farnesiana, Rosa alba*, Koukoumba, P*lumeria alba, Cananga odorata, Pandanus maximus*). Once dried, crush and sort them by size. Prepare coconut oil by grating the coconuts, extracting the milk, letting it sit overnight, removing the cream on top, and then cooking it in a large pot over a wood fire. Once the oil is ready, add the flower mixture (zoukouba) when the temperature has slightly decreased and add Pompeia(R) when the oil is no longer very hot. Let the mixture macerate. The longer the maceration, the more intense the fragrance will be. | Body / skin | Nourishes | 2 | 6 | 1 |  |
|  |  |  |  |  |  |  |  |  | Brightens | 3 | 6 | 1 |  |
|  |  |  |  |  |  |  |  |  | Softens | 2 | 6 | 1 |  |
|  |  |  |  |  |  |  |  |  | Fragrance | 5 | 6 | 1 |  |
|  |  |  |  |  |  |  |  |  | Brightens | 3 | 6 | 1 |  |
|  |  |  |  |  |  |  |  |  | Hydrates | 2 | 6 | 1 |  |
|  |  |  |  |  |  |  | Infuse the dried plants, namely: Flowers of *Jasminum nummulariaefolium, Pandanus maximus, Plumeria alba, Cananga odorata, Rosa alba*, roots of *Chrysopogon zizanoides*, leaves of *Pogostemon cablin, Ayapana triplinervis*, and leaves and inflorescence of *Ocimum spp*., in a decoction of *Cocos nucifera* oil. | Body / skin | Fragrance | 5 | 10 | 1 |  |
|  |  |  |  |  |  |  | Create a necklace with a mix of flowers from *Jasminum nummulariaefolium, Plumeria alba, Cananga odorata, Bougainvillea spectabilis*, and leaves of *Pogostemon cablin*, as well as leaves and inflorescences of *Ocimum spp*. | Neck | Fragrance | 5 | 11 | 1 |  |
|  |  |  |  |  |  |  |  |  | Beautifies | 5 | 11 | 1 |  |
|  |  |  |  |  |  |  | No preparation required | Arrange on the bed | Fragrance | 5 | 12 | 1 |  |
|  |  |  |  |  |  |  |  |  | Well-being | 5 | 12 | 1 |  |
|  |  |  |  |  |  |  |  |  | Fragrance | 5 | 13 | 1 |  |
|  |  |  |  |  |  |  |  |  | Fragrance | 5 | 33 | 1 |  |
|  |  |  |  |  |  |  | Pierce the flowers using a safety pin | Hair | Beautifies | 5 | 13,33 | 2 |  |
|  |  |  |  |  |  |  |  |  | Fragrance | 5 | 13,33 | 2 |  |
|  |  |  |  |  |  |  |  | Clothing | Beautifies | 5 | 13,33 | 2 |  |
|  |  |  |  |  |  |  |  |  | Fragrance | 5 | 13,33 | 2 |  |
|  |  |  |  |  |  |  | Place on the necklaces | Neck | Beautifies | 5 | 33 | 1 |  |
|  |  |  |  |  |  |  |  |  | Fragrance | 5 | 33 | 1 |  |
| *Arecaceae* | *Cocos nucifera* L. | Cocotier | Coconut | M'nadzi | Voaniho |  |  |  |  |  |  |  | ODAM034 |
|  |  |  |  |  |  | Fruit (coprah) |  | Body / skin | Cleanser (soap) | 5 | 8 | 1 |  |
|  |  |  |  |  |  |  | ND | Body / skin (soap) | Cleanser | 5 | 15 | 1 |  |
|  |  |  |  |  |  |  |  | Hair | Cleanser (shampoo) | 4 | 15 | 1 |  |
|  |  |  |  |  |  |  | The inside of the fruit is removed and left to dry in the sun. Once translucent, the peel is separated from the pulp. The pulp is then processed until it forms a paste, which will be used as soap | Body / skin (soap) | Cleanser | 3 | 6 | 1 |  |
|  |  |  |  |  |  |  |  | Hair | Cleanser (shampoo) | 2 | 6 | 1 |  |
|  |  |  |  |  |  | Fruit (oil) | ND | Body/skin | Hydrates | 2 | 2,2 | 2 |  |
|  |  |  |  |  |  |  |  | Hair | Hydrates | 2 | 2 | 1 |  |
|  |  |  |  |  |  |  | After extracting the milk from Cocos nucifera, let it sit until the next day if time allows. Retrieve the upper layer, cook it over low heat until obtaining the oil, and then transfer it into a bottle | Hair | Smoothens | 4 | 9 | 1 |  |
|  |  |  |  |  |  |  |  |  | Nourishes | 4 | 9 | 1 |  |
|  |  |  |  |  |  |  |  |  | Strengthens | 4 | 9 | 1 |  |
|  |  |  |  |  |  |  |  |  | Stimulates hair growth | 4 | 9 | 1 |  |
|  |  |  |  |  |  |  |  | Body / skin | Nourishes | 2 | 9 | 1 |  |
|  |  |  |  |  |  |  |  |  | Hydrates | 2 | 9 | 1 |  |
|  |  |  |  |  |  |  |  |  | Softens | 2 | 9 | 1 |  |
|  |  |  |  |  |  |  | Crack the nut, grate the fruit, and extract the milk with water. Cook the milk until obtaining the oil | Massage | Well-being | 2 | 7 | 1 |  |
|  |  |  |  |  |  |  |  | Body / skin | Well-being | 5 | 7 | 1 |  |
|  |  |  |  |  |  |  |  |  | Hydrates | 2 | 7 | 1 |  |
|  |  |  |  |  |  |  |  |  | Wound | 1 | 7 | 1 |  |
|  |  |  |  |  |  |  |  | Hair | Oil for hair | 4 | 7 | 1 |  |
|  |  |  |  |  |  |  |  |  | Hydrates | 4 | 7 | 1 |  |
|  |  |  |  |  |  |  |  |  | Add shine | 4 | 7 | 1 |  |
|  |  |  |  |  |  |  | Crack the nut, grate the fruit, and extract the milk with water. Cook the milk until obtaining the oil | Hair and style | Hydrates | 4 | 17 | 1 |  |
|  |  |  |  |  |  |  |  |  | Nourishes | 4 | 17 | 1 |  |
|  |  |  |  |  |  |  |  |  | Smoothens | 4 | 17 | 1 |  |
|  |  |  |  |  |  |  | ND | Hair | Hygiene (soap) | 5 | 4 | 1 |  |
|  |  |  |  |  |  |  |  | Face | Hygiene (soap) | 5 | 4 | 1 |  |
|  |  |  |  |  |  |  | Cook the milk until obtaining the oil | Body / skin | Skin care | 2 | 5 | 1 |  |
|  |  |  |  |  |  |  |  | Hair and style | Hair care | 4 | 5 | 1 |  |
|  |  |  |  |  |  |  |  | Wound | Wound protect | 1 | 5 | 1 |  |
|  |  |  |  |  |  |  |  | Body / skin | Sunburn | 3 | 5 | 1 |  |
|  |  |  |  |  |  |  |  |  | Hydrates | 2 | 5 | 1 |  |
|  |  |  |  |  |  |  |  |  | Nourishes | 2 | 14 | 1 |  |
|  |  |  |  |  |  |  |  |  | Nourishes | 2 | 14 | 1 |  |
|  |  |  |  |  |  |  |  |  | Add shine | 3 | 14 | 1 |  |
|  |  |  |  |  |  |  |  | Hair | Hydrates | 2 | 14 | 1 |  |
|  |  |  |  |  |  |  |  |  | Nourishes | 2 | 14 | 1 |  |
|  |  |  |  |  |  |  |  |  | Add shine | 3 | 14 | 1 |  |
|  |  |  |  |  |  |  |  | Scalp | Well-being | 5 | 14 | 1 |  |
|  |  |  |  |  |  |  |  | Body / skin | Fragrance | 5 | 14 | 1 |  |
|  |  |  |  |  |  |  |  |  | Hydrates | 2 | 16 | 1 |  |
|  |  |  |  |  |  |  |  |  | Nourishes | 2 | 16 | 1 |  |
|  |  |  |  |  |  |  |  |  | Warms the skin | 2 | 16 | 1 |  |
|  |  |  |  |  |  |  |  | Face | Hydrates | 2 | 16 | 1 |  |
|  |  |  |  |  |  |  |  |  | Nourishes | 2 | 16 | 1 |  |
|  |  |  |  |  |  |  |  |  | Warms the face | 2 | 16 | 1 |  |
|  |  |  |  |  |  |  |  | Hair and style | Hydrates | 2 | 16,19 | 2 |  |
|  |  |  |  |  |  |  |  |  | Nourishes | 2 | 16,19,19 | 3 |  |
|  |  |  |  |  |  |  |  |  | Smoothens | 4 | 19 | 1 |  |
|  |  |  |  |  |  |  |  |  | Hair growth | 4 | 19 | 1 |  |
|  |  |  |  |  |  |  | Cook the petals of *Rosa alba* in *Cocos nucifera* oil | Body / skin | Beautifies | 5 | 18 | 1 |  |
|  |  |  |  |  |  |  | Crush the rhizome of *Curcuma longa*, the leaves of *Lawsonia inermis*, and the roots of *Chrysopogon zizanoïdes*. Mix everything with coconut milk and oil. Let it marinate for a few minutes or hours | Exfoliation | Cleanser | 5 | 4 | 1 |  |
|  |  |  |  |  |  |  |  |  | Beautifies | 5 | 4 | 1 |  |
|  |  |  |  |  |  |  |  |  | Hydrates | 2 | 4 | 1 |  |
|  |  |  |  |  |  |  |  |  | Softens | 2 | 4 | 1 |  |
|  |  |  |  |  |  |  | Crush the rhizome of *Curcuma longa* and mix it with the scented oil composed of *Cocos nucifera* oil, *Chrysopogon zizanoides* root, flowers of *Pandanus maximus, Jasminum nummulariaefolium*, and fragrant plants from salika | Massage | Cleanser | 5 | 9,12 | 2 |  |
|  |  |  |  |  |  |  |  |  | Massage | 2 | 12 | 1 |  |
|  |  |  |  |  |  |  | Crush the leaves of *Lawsonia inermis* and add the juice of *Citrus sp.* Then apply *Cocos nucifera* oil after rinsing | Apply on the palms, hands, nails, and feet | Beautifies | 5 | 8 | 1 |  |
|  |  |  |  |  |  |  | Crush the leaves of "Sandraouri" et "Andra béyi layi" and mis with *Cocos nucifera* oil. | Face | Anti pimples | 1 | 18 | 1 |  |
|  |  |  |  |  |  |  | Crush the almost dried yellow leaves of *Guettarda speciosa* and put them in *Cocos nucifera* oil | Body / skin | Food allergies causing skin rashes or not | 1 | 26 | 1 |  |
|  |  |  |  |  |  |  | Crush the leaves of *Senna singueana* and mix with *Cocos nucifera* oil. | Body / skin | Skin desorders | 1 | 35 | 1 |  |
|  |  |  |  |  |  |  | ND | Post-depilatory care | Hydrates | 2 | 7 | 1 |  |
|  |  |  |  |  |  |  |  |  | Nourishes | 2 | 7 | 1 |  |
|  |  |  |  |  |  |  |  |  | Softens | 2 | 7 | 1 |  |
|  |  |  |  |  |  |  | Melt beeswax over low heat and mix it with Cocos nucifera oil | Hair and style | Nourishes | 4 | 9,13,3,4,34 | 5 |  |
|  |  |  |  |  |  |  |  |  | Strengthens | 4 | 9,13 | 2 |  |
|  |  |  |  |  |  |  |  |  | Hydrates | 4 | 9,3 | 2 |  |
|  |  |  |  |  |  |  |  |  | Stimulates hair growth | 4 | 9,13,4,34 | 4 |  |
|  |  |  |  |  |  |  |  |  | Add shine | 4 | 13 | 1 |  |
|  |  |  |  |  |  |  |  |  | Beautifies | 4 | 4 | 1 |  |
|  |  |  |  |  |  |  | Macerate *Ceiba pentrada* leaves in *Cocos nucifera* oil | Hair and style | Stimulates hair growth | 4 | 4 | 1 |  |
|  |  |  |  |  |  |  |  |  | Nourishes | 4 | 4 | 1 |  |
|  |  |  |  |  |  |  |  |  | Beautifies | 4 | 4 | 1 |  |
|  |  |  |  |  |  |  | Mix beeswax and Cocos nucifera oil, heat the mixture gently in a pot, and then transfer it to a jar | Hair and style | Nourishes | 4 | 6 | 1 |  |
|  |  |  |  |  |  |  |  |  | Hydrates | 4 | 6 | 1 |  |
|  |  |  |  |  |  |  |  |  | Protect | 4 | 6 | 1 |  |
|  |  |  |  |  |  |  |  |  | Stimulates hair growth | 4 | 6 | 1 |  |
|  |  |  |  |  |  |  | Grate the coconut and extract the milk, cook it, and collect the oil | Hair | Nourishes | 2 | 26 | 1 |  |
|  |  |  |  |  |  |  |  |  | Hydrates | 2 | 26 | 1 |  |
|  |  |  |  |  |  |  |  |  | Sun protection | 3 | 26 | 1 |  |
|  |  |  |  |  |  |  |  | Body / skin | Nourishes | 2 | 26 | 1 |  |
|  |  |  |  |  |  |  |  |  | Hydrates | 2 | 26 | 1 |  |
|  |  |  |  |  |  |  |  |  | Sun protection | 3 | 26 | 1 |  |
|  |  |  |  |  |  |  | ND | Massage (such as koussinga during and after childbirth) | Skin care | 2 | 2 | 1 |  |
|  |  |  |  |  |  |  |  |  | Beautifies | 5 | 2 | 1 |  |
|  |  |  |  |  |  |  |  |  | Hydrates | 2 | 2 | 1 |  |
|  |  |  |  |  |  |  |  | Body / skin | Oil for body | 5 | 3 | 1 |  |
|  |  |  |  |  |  |  |  |  | Hydrates | 2 | 3 | 1 |  |
|  |  |  |  |  |  |  |  |  | Nourishes | 2 | 3 | 1 |  |
|  |  |  |  |  |  |  |  |  | Softens | 2 | 3 | 1 |  |
|  |  |  |  |  |  |  |  |  | Sun protection | 3 | 3 | 1 |  |
|  |  |  |  |  |  |  |  | Body | Massage | 2 | 8 | 1 |  |
|  |  |  |  |  |  |  |  | Feet | Massage | 2 | 8 | 1 |  |
|  |  |  |  |  |  |  |  | Hair and style | Hydrates | 2 | 8 | 1 |  |
|  |  |  |  |  |  |  |  |  | Nourishes | 2 | 8 | 1 |  |
|  |  |  |  |  |  |  |  | Hair and style | Hydrates | 2 | 10 | 1 |  |
|  |  |  |  |  |  |  |  |  | Nourishes | 2 | 10 | 1 |  |
|  |  |  |  |  |  |  |  |  | Strengthens | 4 | 10 | 1 |  |
|  |  |  |  |  |  |  |  | Body / skin | Cleanser | 5 | 10 | 1 |  |
|  |  |  |  |  |  |  |  |  | Nourishes | 2 | 10 | 1 |  |
|  |  |  |  |  |  |  |  | Hair and style | Nourishes | 2 | 13 | 1 |  |
|  |  |  |  |  |  |  |  |  | Warm the scalp | 5 | 13 | 1 |  |
|  |  |  |  |  |  |  |  |  | Sun protection | 3 | 13 | 1 |  |
|  |  |  |  |  |  |  |  | Body / skin | Hydrates | 2 | 13 | 1 |  |
|  |  |  |  |  |  |  |  | Post-depilatory care | Hydrates | 2 | 13 | 1 |  |
|  |  |  |  |  |  |  |  |  | Add shine | 3 | 13 | 1 |  |
|  |  |  |  |  |  |  |  |  | Relieves | 5 | 13 | 1 |  |
|  |  |  |  |  |  |  |  | Body / skin | Hydrates | 2 | 20 | 1 |  |
|  |  |  |  |  |  |  |  |  | Nourishes | 2 | 20 | 1 |  |
|  |  |  |  |  |  |  |  | Hair and style | Hydrates | 2 | 20,27 | 2 |  |
|  |  |  |  |  |  |  |  |  | Nourishes | 2 | 27 | 1 |  |
|  |  |  |  |  |  |  |  |  | Add shine | 3 | 27 | 1 |  |
|  |  |  |  |  |  |  |  | Body / skin | Oil for body | 2 | 27 | 1 |  |
|  |  |  |  |  |  |  |  |  | Hydrates | 2 | 27 | 1 |  |
|  |  |  |  |  |  |  |  |  | Add shine | 3 | 27 | 1 |  |
|  |  |  |  |  |  |  |  | Hair | Shampoo | 4 | 32 | 1 |  |
|  |  |  |  |  |  |  | Macerate cloves of *Syzygium aromaticum* in *Cocos nucifera* oil for a few days or weeks | Hair | Stimulates hair growth | 4 | 16 | 1 |  |
|  |  |  |  |  |  |  |  |  | Smoothens | 4 | 16 | 1 |  |
|  |  |  |  |  |  |  | Mix *Aloe vera* gel and *Cocos nucifera* oil | Body / skin | Skin care | 2 | 18 | 1 |  |
|  |  |  |  |  |  |  |  |  | Refreshing | 5 | 32 | 1 |  |
|  |  |  |  |  |  |  |  |  | Well-being | 5 | 32 | 1 |  |
|  |  |  |  |  |  |  |  |  | Anti -aging | 2 | 32 | 1 |  |
|  |  |  |  |  |  |  |  |  | Firms | 2 | 32 | 1 |  |
|  |  |  |  |  |  |  | Mix Sabaravatsi leaves with Cocos nucifera oil | Cleanse and apply to the affected area with | Allergies forming sort of plaques | 1 | 28 | 1 |  |
|  |  |  |  |  |  |  | Mix the following plants: *Chrysopogon zizanoides* root, leaves and inflorescences of *Ocimum* spp., *Ayapana triplinervis* leaves, flowers of *Jasminum nummulariaefolium, Pandanus maximus, Cananga odorata*; all sourced from safety pins. Infuse them into *Cocos nucifera* oil and let it macerate in the oil | Body / skin | Beautifies | 5 | 5 | 1 |  |
|  |  |  |  |  |  |  |  |  | Fragrance | 5 | 5 | 1 |  |
|  |  |  |  |  |  |  |  |  | Softens | 2 | 5 | 1 |  |
|  |  |  |  |  |  |  |  |  | Depilatory care | 4 | 5 | 1 |  |
|  |  |  |  |  |  |  | Mix the leaves and inflorescences of *Ocimum* spp. with the flowers of *Pandanus maximus, Rosa alba, Jasminum nummulariaefolium, Cananga odorata*, and the roots of C*hrysopogon zizanoides*. Incorporate everything into *Cocos nucifera* oil and let it macerate | Body / skin | Beautifies (face) | 5 | 4 | 1 |  |
|  |  |  |  |  |  |  |  |  | Beautifies | 5 | 4 | 1 |  |
|  |  |  |  |  |  |  |  |  | Fragrance | 5 | 4 | 1 |  |
|  |  |  |  |  |  |  | Mix *Cocos nucifera* oil with the leaves and inflorescences of *Ocimum* spp. (Mkadi mena or malandi) and let it macerate | Body / skin | Cleanser | 5 | 3 | 1 |  |
|  |  |  |  |  |  |  |  |  | Softens | 2 | 3 | 1 |  |
|  |  |  |  |  |  |  |  |  | Post-depilatory care | 4 | 3 | 1 |  |
|  |  |  |  |  |  |  |  |  | Fragrance | 5 | 7 | 1 |  |
|  |  |  |  |  |  |  |  |  | Hydrates | 2 | 7 | 1 |  |
|  |  |  |  |  |  |  |  |  | Protect | 2 | 7 | 1 |  |
|  |  |  |  |  |  |  | Place Vanilla sp pods in Cocos nucifera oil and let it macerate for several weeks |  | Fragrance | 5 | 7,18 | 2 |  |
|  |  |  |  |  |  |  |  |  | Hydrates | 2 | 7 | 1 |  |
|  |  |  |  |  |  |  |  |  | Protect | 5 | 7 | 1 |  |
|  |  |  |  |  |  |  |  |  | Hydrates (face) | 2 | 7 | 1 |  |
|  |  |  |  |  |  |  |  |  | Protect (face) | 2 | 7 | 1 |  |
|  |  |  |  |  |  |  |  |  | Well-being | 5 | 18 | 1 |  |
|  |  |  |  |  |  |  | Infuse the dried plants, including flowers of *Jasminum nummulariaefolium, Pandanus maximus, Plumeria alba, Cananga odorata, Rosa alba*, roots of *Chrysopogon zizanoides*, leaves of *Pogostemon cablin, Ayapana triplinervis*, and the leaves and inflorescence of *Ocimum* spp., in *Cocos nucifera* oil | Body / skin | Fragrance | 5 | 10 | 1 |  |
|  |  |  |  |  |  |  | Dry the leaves of *Phymatosorus scolopendria* and the flowers of *Jasminum nummulariaefolium*, then sift. Put them in *Cocos nucifera* oil and let it macerate | Body / skin | Hydrates | 2 | 23 | 1 |  |
|  |  |  |  |  |  |  |  |  | Cleanser | 5 | 23 | 1 |  |
|  |  |  |  |  |  |  |  |  | Beautifies | 5 | 23 | 1 |  |
|  |  |  |  |  |  |  |  |  | Skin care | 2 | 23 | 1 |  |
|  |  |  |  |  |  |  |  |  | Softens | 2 | 23 | 1 |  |
|  |  |  |  |  |  |  |  |  | Nourishes | 2 | 23 | 1 |  |
|  |  |  |  |  |  |  |  |  | Fragrans | 5 | 23 | 1 |  |
|  |  |  |  |  |  |  | Take the mixture of plants from weddings or create your blend based on the availability of plants (*Chrysopogon zizanoides* root, *Ayapana triplinervis* leaves, leaves and inflorescences of *Ocimum* spp., flowers of *Jasminum nummulariaefolium, Vachellia farnesiana, Rosa alba, Koukoumba, Plumeria alba, Cananga odorata, Pandanus maximus*). Dry them, crush, and sort by size. Prepare coconut oil. Grate the coconuts ==> extract the milk ==> let it rest overnight ==> remove the cream on top ==> cook in a large pot over a wood fire ==> Once the oil is ready, add the flower mixture (zoukouba) when the temperature has cooled a bit, and add the pompeia when the oil is no longer very hot. Let it all macerate. The longer the maceration, the more intense the fragrance. | Body / skin | Nourishes | 2 | 6 | 1 |  |
|  |  |  |  |  |  |  |  |  | Add shine | 3 | 6 | 1 |  |
|  |  |  |  |  |  |  |  |  | Softens | 2 | 6 | 1 |  |
|  |  |  |  |  |  |  |  |  | Fragrans | 5 | 6 | 1 |  |
|  |  |  |  |  |  |  |  |  | Lightens | 3 | 6 | 1 |  |
|  |  |  |  |  |  |  |  |  | Hydrates | 2 | 6 | 1 |  |
|  |  |  |  |  |  |  | When the *Cocos nucifera* oil is heated, take the dried roots of *Chrysopogon zizanoides*, crush them, and put them in the oil. Mix and let it macerate for about two weeks | Body / skin | Hydrates | 2 | 6 | 1 |  |
|  |  |  |  |  |  |  |  |  | Relaxes the muscles | 5 | 12 | 1 |  |
|  |  |  |  |  |  |  |  |  | Loosens the muscles | 5 | 18 | 1 |  |
|  |  |  |  |  |  |  |  |  | Fragrans | 5 | 12 | 1 |  |
|  |  |  |  |  |  |  | Grate the inside of the coconut, extract the milk, cook over a wood fire, and collect the coconut oil | Body / skin | Hydrates | 2 | 1 | 1 |  |
|  |  |  |  |  |  |  |  |  | Protect wound | 1 | 1 | 1 |  |
|  |  |  |  |  |  |  |  | Hair | Hydrates | 2 | 1 | 1 |  |
|  |  |  |  |  |  |  | Grate the coconuts, extract the milk, let it rest overnight, remove the cream on top, cook in a large pot over a wood fire until obtaining oil | Body / skin | Hydrates | 2 | 6 | 1 |  |
|  |  |  |  |  |  |  |  |  | Nourishes | 2 | 6 | 1 |  |
|  |  |  |  |  |  |  |  | Hair and style | Nourishes | 2 | 6 | 1 |  |
|  |  |  |  |  |  |  |  |  | Add shine | 3 | 6 | 1 |  |
|  |  |  |  |  |  |  |  |  | Beautifies | 5 | 6 | 1 |  |
|  |  |  |  |  |  |  |  |  | Strengthens | 4 | 6 | 1 |  |
|  |  |  |  |  |  | Fruit (milk) | Crush the tuber of "toubou antani" and mix it with *Cocos nucifera* milk | Body / skin | Anti pimples | 1 | 15 | 1 |  |
|  |  |  |  |  |  |  | Crush the rhizome of *Curcuma longa* or mix the powder of *Curcuma longa* with C*ocos nucifera* milk | Massage | Cleanser | 5 | 16 | 1 |  |
|  |  |  |  |  |  |  |  |  | Brightens | 3 | 16 | 1 |  |
|  |  |  |  |  |  |  |  |  | Add shine | 3 | 16 | 1 |  |
|  |  |  |  |  |  |  |  |  | Cleanser (face) | 5 | 16 | 1 |  |
|  |  |  |  |  |  |  |  |  | Brightens (face) | 3 | 16 | 1 |  |
|  |  |  |  |  |  |  |  |  | Adds shine (face) | 3 | 16 | 1 |  |
|  |  |  |  |  |  |  |  |  | Cleanser (face) | 5 | 20 | 1 |  |
|  |  |  |  |  |  |  |  |  | Beautifies (face) | 5 | 20 | 1 |  |
|  |  |  |  |  |  |  |  |  | Nourishes (face) | 2 | 20 | 1 |  |
|  |  |  |  |  |  |  |  |  | Brightens (face) | 3 | 20 | 1 |  |
|  |  |  |  |  |  |  | Crush the rhizome of *Curcuma longa*. Add "zoukouba", coconut milk. Mix everything together | Exfoliation | Cleanser | 5 | 21 | 1 |  |
|  |  |  |  |  |  |  |  |  | Nourishes | 2 | 21 | 1 |  |
|  |  |  |  |  |  |  | Crush the leaves of *Lawsonia inermis* and the rhizome of *Curcuma longa,* then mix them with *Cocos nucifera* milk | Massage | Cleanser | 5 | 16 | 1 |  |
|  |  |  |  |  |  |  |  |  | Brightens | 3 | 16 | 1 |  |
|  |  |  |  |  |  |  |  |  | Add shine | 3 | 16 | 1 |  |
|  |  |  |  |  |  |  |  |  | Cleanser (face) | 5 | 16 | 1 |  |
|  |  |  |  |  |  |  |  |  | Brightens (face) | 3 | 16 | 1 |  |
|  |  |  |  |  |  |  |  |  | Adds shine (face) | 3 | 16 | 1 |  |
|  |  |  |  |  |  |  | Crush the leaves of *Lawsonia inermis* and mix them with *Cocos nucifera* milk and S*esamum indicum* oil | Massage | Cleanser | 5 | 16 | 1 |  |
|  |  |  |  |  |  |  |  |  | Brightens | 3 | 16 | 1 |  |
|  |  |  |  |  |  |  |  |  | Add shine | 3 | 16 | 1 |  |
|  |  |  |  |  |  |  |  |  | Cleanser (face) | 5 | 16 | 1 |  |
|  |  |  |  |  |  |  |  |  | Brightens (face) | 3 | 16 | 1 |  |
|  |  |  |  |  |  |  |  |  | Adds shine (face) | 3 | 16 | 1 |  |
|  |  |  |  |  |  |  | Crush the leaves of *Lawsonia inermis* and the rhizome of *Curcuma longa*, then mix them with *Cocos nucifera* milk and *Sesamum indicum* oil | ND | Brightens | 3 | 16 | 1 |  |
|  |  |  |  |  |  |  |  |  | Add shine | 3 | 16 | 1 |  |
|  |  |  |  |  |  |  |  |  | Cleanser (face) | 5 | 16 | 1 |  |
|  |  |  |  |  |  |  |  |  | Brightens (face) | 3 | 16 | 1 |  |
|  |  |  |  |  |  |  |  |  | Adds shine (face) | 3 | 16 | 1 |  |
|  |  |  |  |  |  |  | Crush the leaves of *Lawsonia inermis*, extract the milk from *Cocos nucifera*, crush the *Curcuma longa*. Mix it all together | Massage | Beautifies | 5 | 17 | 1 |  |
|  |  |  |  |  |  |  |  |  | Tanned complexion | 3 | 17 | 1 |  |
|  |  |  |  |  |  |  |  |  | Brightens | 3 | 17 | 1 |  |
|  |  |  |  |  |  |  |  |  | Beautifies (face) | 5 | 17 | 1 |  |
|  |  |  |  |  |  |  |  |  | Tanned complexion (face) | 3 | 17 | 1 |  |
|  |  |  |  |  |  |  |  |  | Brightens (face) | 3 | 17 | 1 |  |
|  |  |  |  |  |  |  | Crush the leaves of *Ipomoea batatas* in coconut milk | Apply to the hair and leave it on for an hour, then rinse | Softens | 4 | 15 | 1 |  |
|  |  |  |  |  |  |  |  |  | Detangles | 4 | 23 | 1 |  |
|  |  |  |  |  |  |  |  |  | Shampoo | 4 | 23 | 1 |  |
|  |  |  |  |  |  |  | Mix coconut milk and blend it with Turmeric | Apply to the body and leave it on overnight, then wash off with warm water | Cleanser | 5 | 13 | 1 |  |
|  |  |  |  |  |  |  |  |  | Brightens | 3 | 13 | 1 |  |
|  |  |  |  |  |  |  | Mix coconut milk, the rhizome of Turmeric, and "Zoukouba" | Massage | Add shine | 3 | 14 | 1 |  |
|  |  |  |  |  |  |  |  |  | Beautifies | 5 | 14 | 1 |  |
|  |  |  |  |  |  |  |  |  | Adds shine (face) | 3 | 14 | 1 |  |
|  |  |  |  |  |  |  |  |  | Beautifies (face) | 5 | 14 | 1 |  |
|  |  |  |  |  |  | Fruit (milk, grated fruit) | Crush the leaves and inflorescences of *Ocimum* spp., the rhizome of *Curcuma longa*, the roots of *Chrysopogon zizanoides*, the flowers of *Rosa alba, Pandanus maximus, Jasminum nummulariaefolium*, and *Vachellia farnesiana*. Mix everything with coconut milk and grated fruit of *Cocos nucifera.* Let it macerate for a few minutes or even hours | Exfoliation | Skin care | 2 | 5 | 1 |  |
|  |  |  |  |  |  |  |  |  | Cleanser | 5 | 5 | 1 |  |
|  |  |  |  |  |  |  |  |  | Softens | 2 | 5 | 1 |  |
|  |  |  |  |  |  |  |  |  | Nourishes | 2 | 5 | 1 |  |
|  |  |  |  |  |  |  |  |  | Beautifies | 5 | 5 | 1 |  |
|  |  |  |  |  |  |  |  |  | Hydrates | 2 | 5 | 1 |  |
|  |  |  |  |  |  | Fruit (milk, pulp) | Mix coconut milk and coconut pulp with "ka" powder | Exfoliation | Cleanser | 5 | 4 | 1 |  |
|  |  |  |  |  |  |  |  |  | Hydrates | 2 | 4 | 1 |  |
|  |  |  |  |  |  |  |  |  | Softens | 2 | 4 | 1 |  |
|  |  |  |  |  |  |  | Mix coconut milk, pulp from the fruit of *Adansonia digitata*, and also incorporate honey. Add more coconut milk to ensure the mixture is smooth. | Massage | Well-being | 5 | 7,7,7 | 3 |  |
|  |  |  |  |  |  | Fruit (oil, milk, grated) | Crush the rhizome of *Curcuma longa*. Add "zoukouba", milk, oil, and grated fruit of *Cocos nucifera*. Mix everything together | Exfoliation | Cleanser | 5 | 6 | 1 |  |
|  |  |  |  |  |  |  |  |  | Hydrates | 2 | 6 | 1 |  |
|  |  |  |  |  |  |  |  |  | Nourishes | 2 | 6 | 1 |  |
|  |  |  |  |  |  |  |  |  | Softens | 2 | 6 | 1 |  |
|  |  |  |  |  |  |  |  |  | Add shine | 3 | 6 | 1 |  |
|  |  |  |  |  |  |  | Crush the rhizome of *Curcuma longa* and mix it with the pulp of the fruit of *Adansonia digitata*, coconut milk, and grated fruit of *Cocos nucifera* until obtaining a fairly smooth paste | Massage and let it rest for a few minutes. Rinse | Beautifies | 5 | 27,27 | 2 |  |
|  |  |  |  |  |  |  |  |  | Well-being | 5 | 27,27 | 2 |  |
|  |  |  |  |  |  |  |  |  | Tanned complexion | 3 | 27,27 | 2 |  |
|  |  |  |  |  |  |  |  |  | Brightens | 3 | 27,27 | 2 |  |
|  |  |  |  |  |  |  |  |  | Hydrates | 2 | 27,27 | 2 |  |
|  |  |  |  |  |  |  |  |  | Smoothens | 2 | 27,27 | 2 |  |
|  |  |  |  |  |  |  |  |  | Beautifies | 5 | 27,27 | 2 |  |
|  |  |  |  |  |  |  | Crush the leaves of *Lawsonia inermis* and the rhizome of *Curcuma longa*, then mix them with oil, milk, and the fruit of *Cocos nucifera* | Circular massage | Cleanser | 5 | 8 | 1 |  |
|  |  |  |  |  |  |  |  |  | Smoothens | 2 | 8 | 1 |  |
|  |  |  |  |  |  |  |  |  | Hydrates | 2 | 8 | 1 |  |
|  |  |  |  |  |  |  |  |  | Nourishes | 2 | 8 | 1 |  |
|  |  |  |  |  |  |  |  |  | Beautifies | 5 | 8 | 1 |  |
|  |  |  |  |  |  |  | Crush the leaves of *Lawsonia inermis*, the roots of *Chrysopogon zizanoides*, and the rhizome of *Curcuma longa*. Mix everything with the oil and milk of *Cocos nucifera* | Exfoliation | Cleanser | 5 | 23 | 1 |  |
|  |  |  |  |  |  |  |  |  | Beautifies | 5 | 23 | 1 |  |
|  |  |  |  |  |  |  |  |  | Hydrates | 2 | 23 | 1 |  |
|  |  |  |  |  |  |  |  |  | Softens | 2 | 23 | 1 |  |
|  |  |  |  |  |  | Fruit (water) | Not preparation recquired | Hair | Cleanser | 5 | 15 | 1 |  |
|  |  |  |  |  |  |  |  |  | Detangles | 4 | 15 | 1 |  |
|  |  |  |  |  |  |  |  |  | Shampoo | 4 | 19 | 1 |  |
| *Asteraceae* | *Ayapana triplinervis* (Vahl) R.M.King & H.Rob. | Ayapana, yapana | Ayapana | Mlaliyapana | Mlaliyapana |  |  |  |  |  |  |  | ODAM009 |
|  |  |  |  |  |  | Leaf | ND | ND | ND | ND | 4,23 | 2 |  |
|  |  |  |  |  |  |  | Mix all the following plants: roots of *Chrysopogon zizanoides,* leaves and inflorescences of *Ocimum* spp., leaves of *Ayapana triplinervis*, flowers of J*asminum nummulariaefolium, Pandanus maximus, Cananga odorata*; extracted from safety pins. Infuse them in *Cocos nucifera* oil and let it macerate in the oil | Body / skin | Beautifies | 5 | 5 | 1 |  |
|  |  |  |  |  |  |  |  |  | Fragrance | 5 | 5 | 1 |  |
|  |  |  |  |  |  |  |  |  | Softens | 2 | 5 | 1 |  |
|  |  |  |  |  |  |  |  |  | Depilatory care | 4 | 5 | 1 |  |
|  |  |  |  |  |  |  | Take the mixture of plants from weddings or create your blend based on the availability of plants (*Chrysopogon zizanoides* root, *Ayapana triplinervis* leaves, leaves and inflorescences of *Ocimum* spp., flowers of J*asminum nummulariaefolium, Vachellia farnesiana, Rosa alba,* "Koukoumba", *Plumeria alba*, *Cananga odorata, Pandanus maximus*). Dry them, crush, and sort by size. Prepare coconut oil. Grate the coconuts ==> extract the milk ==> let it rest overnight ==> remove the cream on top ==> cook in a large pot over a wood fire ==> Once the oil is ready, add the flower mixture "zoukouba" when the temperature has cooled a bit, and add the pompeia when the oil is no longer very hot. Let it all macerate. The longer the maceration, the more intense the fragrance. | Body / skin | Nourishes | 2 | 6 | 1 |  |
|  |  |  |  |  |  |  |  |  | Add shine | 3 | 6 | 1 |  |
|  |  |  |  |  |  |  |  |  | Softens | 2 | 6 | 1 |  |
|  |  |  |  |  |  |  |  |  | Fragrans | 5 | 6 | 1 |  |
|  |  |  |  |  |  |  |  |  | Lightens | 3 | 6 | 1 |  |
|  |  |  |  |  |  |  |  |  | Hydrates | 2 | 6 | 1 |  |
|  |  |  |  |  |  |  | Infuse the dried plants, including the flowers of *Jasminum nummulariaefolium, Pandanus maximus, Plumeria alba, Cananga odorata, Rosa alba,* the roots of *Chrysopogon zizanoides*, the leaves of *Pogostemon cablin, Ayapana triplinervis*, and the leaves and inflorescence of *Ocimum* spp., in *Cocos nucifera* oil | Body / skin | Fragrance | 5 | 10 | 1 |  |
|  |  |  |  |  |  |  | Mix the dried flowers of Pandanus maximus, Jasminum nummulariaefolium, Cananga odorata, the dried leaves of Pogostemon cablin, Ayapana triplinervis, the dried leaves and inflorescences of Ocimum spp., and the root of Chrysopogon zizanoides | Body / skin | Fragrance | 5 | 11 | 1 |  |
| *Asteraceae* | *Sigesbeckia orientalis* L. | Colle colle | Sigesbeckia herba |  | Tei lamba |  |  |  |  |  |  |  | ODAM035 |
|  |  |  |  |  |  | Whole plant | Crush the entire plant until sweating occurs | Face | Anti pimples | 1 | 33 | 1 |  |
| *Asteraceae* | *Youngia japonica* (L.) DC. | Lastron bâtard | ND | ND | Sari féliki guissi |  |  |  |  |  |  |  | ODAM036 |
|  |  |  |  |  |  | Leaf | Crush the leaves in water | Body / skin | Spots with lesions. Toxique plant | 1 | 35 | 1 |  |
| *Bixaceae* | *Bixa orellana* L. | Roucouyer | Annatto | ND | M'jenguéfuré |  |  |  |  |  |  |  | NC |
|  |  |  |  |  |  | Seed | Apply the seeds directly on the lips | Face | Color lips | 5 | 6,12,16,18 | 4 |  |
| *Bombacaceae* | *Adansonia digitata* L. | Baobab | Baobab tree | Buyu | Buyu |  |  |  |  |  |  |  | ODAM016 |
|  |  |  |  |  |  | Fruit (pulp) | Mix the coconut milk, the pulp of *Adansonia digitata* fruit, and also incorporate honey. Add more coconut milk to make the mixture smooth | Massage | Well-being | 5 | 7,7,7 | 3 |  |
|  |  |  |  |  |  |  | ND | Body / skin | Cleanser | 5 | 10 | 1 |  |
|  |  |  |  |  |  |  |  |  | Softens | 2 | 10 | 1 |  |
|  |  |  |  |  |  |  | Split the fruit in two, extract the pulp, and mix it with water until you obtain a smooth paste | Apply on face during 3 hours | Cleanser | 5 | 14 | 1 |  |
|  |  |  |  |  |  |  |  |  | Beautifies | 5 | 14 | 1 |  |
|  |  |  |  |  |  |  | Crush the rhizome of *Curcuma longa* and mix it with the pulp of *Adansonia digitata* fruit, as well as the milk and grated fruit of *Cocos nucifera*. Combine all the ingredients until you obtain a fairly smooth paste | Body / skin | Beautifies |  | 27,27,27 | 3 |  |
|  |  |  |  |  |  |  |  |  | Well-being | 5 | 27 | 1 |  |
|  |  |  |  |  |  |  |  |  | Tanned complexion | 3 | 27 | 1 |  |
|  |  |  |  |  |  |  |  |  | Brightens | 3 | 27 | 1 |  |
|  |  |  |  |  |  |  |  |  | Hydrates | 2 | 27 | 1 |  |
|  |  |  |  |  |  |  |  |  | Softens | 2 | 27 | 1 |  |
|  |  |  |  |  |  |  |  |  | Anti-aging | 2 | 27 | 1 |  |
|  |  |  |  |  |  | Piece of wood | Scrape the piece of wood on the dead coral stone with a little water until you obtain a smooth paste | Body / skin | Anti pimples | 1 | 21 | 1 |  |
|  |  |  |  |  |  |  |  |  | Scabies | 1 | 21,28 | 2 |  |
| *Burseraceae* | *Commiphora arafy* H.Perrier | ND | ND | M'ri ombany | Matyambelo |  |  |  |  |  |  |  | ODAM037 |
|  |  |  |  |  |  | Seed | Crush the seeds of *Commiphora arafy* and the leaves of *Gambeya boiviniana*, then put them in water | Mouthwash | Toothaches | 5 | 35 | 1 |  |
| *Calophyllaceae* | *Calophyllum inophyllum* L. | Takamaka | Alexandrian laurel | M'tondro | M'tondro |  |  |  |  |  |  |  | ODAM022 |
|  |  |  |  |  |  | Sap | Make an incision in the tree 24 to 48 hours prior and collect the sap in a container | Apply on the hair and gradually pull it off | Depilatory |  | 5,6,8,13,30 |  |  |
|  |  |  |  |  |  |  | Make an incision in the tree 24 to 48 hours prior and collect the sap in a container | Apply on the hair and gradually pull it off. Then aplly Cocos nusifera oil | Depilatory |  | 7 |  |  |
|  |  |  |  |  |  |  | Extract the sap from the tree | Depilatory wax | Depilatory |  | 24,24,32 |  |  |
|  |  |  |  |  |  |  | ND | ND | Depilatory |  | 1,2 |  |  |
| *Convolvulaceae* | *Decalobanthus peltatus* (L.) A.R.Simões & Staples | Décalobanthe pelté | Merremia | Vahi bé | Vahi bé |  |  |  |  |  |  |  | ODAM038 |
|  |  |  |  |  |  | Leaf | Decoction | Scalp and style | Stimulates hair growth | 4 | 10 | 1 |  |
|  |  |  |  |  |  |  |  |  | Add shine | 4 | 10 | 1 |  |
|  |  |  |  |  |  |  |  |  | Strengthens | 4 | 10 | 1 |  |
|  |  |  |  |  |  |  |  |  | Smoothens | 4 | 10 | 1 |  |
|  |  |  |  |  |  |  | Decoction | To wash the vagina with a warm decoction | Eliminate vaginal odor | 5 | 15 | 1 |  |
|  |  |  |  |  |  |  | Crush the leaves and add them to the water | Apply the aqueous paste of the leaves to the scalp, massage gently, and rinse | Add shine | 4 | 30 | 1 |  |
|  |  |  |  |  |  |  |  |  | Anti-dandruff | 4 | 30 | 1 |  |
| *Convolvulaceae* | *Ipomoea batatas* (L.) Lam. | Patate douce | Sweet potato | Batata | Batata |  |  |  |  |  |  |  | ODAM039 |
|  |  |  |  |  |  | Leaf | Crush the leaves of *Ipomoea batatas* in *Cocos nucifera* milk | Apply to the hair and leave it on for one hour, then rinse | Cleanser | 5 | 15 | 1 |  |
|  |  |  |  |  |  |  |  |  | Nourishes | 2 | 15 | 1 |  |
|  |  |  |  |  |  |  |  |  | Detangles | 4 | 15,22 | 2 |  |
|  |  |  |  |  |  |  |  |  | Strengthens | 4 | 15 | 1 |  |
|  |  |  |  |  |  |  |  |  | Softens | 4 | 15 | 1 |  |
|  |  |  |  |  |  |  |  |  | Shampoo | 4 | 22 | 1 |  |
| *Convolvulaceae* | *Ipomoea obscura* (L.) Ker Gawl. | Ipomée obscure | Obscure morning glory | Hovéani | Hovéani |  |  |  |  |  |  |  | *ODAM004* |
|  |  |  |  |  |  | Leaf | Crush the leaves in water until thickened, then filter the mixture | Apply to wet or dry hair, massage gently, and then rinse | Cleanser | 4 | 6 | 1 |  |
|  |  |  |  |  |  |  |  |  | Softens | 4 | 6 | 1 |  |
|  |  |  |  |  |  |  |  |  | Strengthens | 4 | 6 | 1 |  |
|  |  |  |  |  |  |  |  |  | Smoothens | 4 | 6 | 1 |  |
|  |  |  |  |  |  |  |  |  | Nourishes | 4 | 6 | 1 |  |
|  |  |  |  |  |  |  |  |  | Stimulates hair growth | 4 | 6 | 1 |  |
|  |  |  |  |  |  |  |  |  | Add shine | 4 | 6 | 1 |  |
|  |  |  |  |  |  |  |  |  | Anti-dandruff | 4 | 6 | 1 |  |
|  |  |  |  |  |  |  |  | Apply to the hair and gently massage the scalp. Remember to brush or comb the hair to detangle it well, then rinse | Smoothens | 4 | 9 | 1 |  |
|  |  |  |  |  |  |  |  |  | Detangles | 4 | 9 | 1 |  |
|  |  |  |  |  |  |  |  |  | Nourishes | 4 | 9 | 1 |  |
|  |  |  |  |  |  |  |  |  | Add shine | 4 | 9 | 1 |  |
|  |  |  |  |  |  |  |  |  | Beautifies | 4 | 9 | 1 |  |
|  |  |  |  |  |  |  |  |  | Strengthens | 4 | 9 | 1 |  |
|  |  |  |  |  |  |  |  |  | Stimulates hair growth | 4 | 9 | 1 |  |
|  |  |  |  |  |  |  |  |  | Refreshes the scalp | 4 | 9 | 1 |  |
|  |  |  |  |  |  |  |  | Shampoo | Smoothens | 4 | 11 | 1 |  |
|  |  |  |  |  |  |  |  |  | Detangles | 4 | 11 | 1 |  |
|  |  |  |  |  |  |  |  |  | Shampoo | 4 | 11 | 1 |  |
|  |  |  |  |  |  |  |  | Apply to the scalp and massage by rubbing in case of conditions with patches | scalp ringworm | 1 | 29 | 1 |  |
| *Convolvulaceae* | *Ipomoea pes-caprae* (L.) R.Br. | Patate à Durand | Beach morning glory | ND | Lalanda |  |  |  |  |  |  |  | ODAM040 |
|  |  |  |  |  |  | Leaf | Decoction | Scalp | Prevent hair loss | 4 | 32 | 1 |  |
| *Convolvulaceae* | *Ipomoea nil* (L.) Roth | Ipomée du nil | Blue morning-glory | ND | Antaka mawaridi |  |  |  |  |  |  |  | ODAM041 |
|  |  |  |  |  |  | Leaf | Crush the fresh leaves | Burns | Accelerates healing | 1 | 33 | 1 |  |
| *Crassulaceae* | *Kalanchoe pinnata* (Lam.) Pers. | Kalanchoé penné | ND | Miawani | Sudifafgna |  |  |  |  |  |  |  | ODAM042 |
|  |  |  |  |  |  | Leaf | Crush the leaves until sweating occurs | Apply to the patches (body) | Skin care | 1 | 1 | 1 |  |
|  |  |  |  |  |  |  |  | Small wound | Skin care | 1 | 1 | 1 |  |
|  |  |  |  |  |  |  | Burn the dried ravi makoutchi, place the leaves of Kalanchoe pinnata above the fire until they wilt, and let them rest for 2 minutes | Apply directly to the skin by gently patting | Firms | 2 | 17 | 1 |  |
|  |  |  |  |  |  |  |  |  | Beautifies | 5 | 17 | 1 |  |
| *Cucurbitaceae* | *Kedrostis elongata* Keraudren | ND | ND | Bahi bahi | Tumbou antani |  |  |  |  |  |  |  | NC |
|  |  |  |  |  |  | Leaf | Crush the leaves | Apply on pustules, such as scabies | Heals the pimples | 1 | 35 | 1 |  |
| *Euphorbiaceae* | *Acalypha indica* L. | Acalyphe d'Inde, herbe chat | Indian acalypha |  |  |  |  |  |  |  |  |  | NC |
|  |  |  |  |  |  | Leaf | Crush the leaves until sweating occurs | Scalp before cut hair | scalp ringworm | 4 | 29 | 1 |  |
|  |  |  |  |  |  | Steem | Crush the leaves until sweating occurs | Scalp before cut hair | scalp ringworm | 4 | 29 | 1 |  |
| *Euphorbiaceae* | *Croton mayottae* P. E. Berry & Kainul | Croton glanduleux de Mayotte | ND | Muhuve | Sari laza laza |  |  |  |  |  |  |  | ODAM043 |
|  |  |  |  |  |  | Root | Scrape the root on the dead coral stone with a little water until you obtain a smooth paste | Face | Anti pimples | 1 | 2 | 1 |  |
|  |  |  |  |  |  |  |  | Body / skin | Treat "mwili wa moro" | 1 | 2 | 1 |  |
| *Euphorbiaceae* | *Jatropha curcas* L. | Pignon d'inde | Purging nut | Muri maji | Valavelo |  |  |  |  |  |  |  | NC |
|  |  |  |  |  |  | Bark | Decoction until the water turns red | Mouthwash | Antimicrobial | 1 | 8 | 1 |  |
|  |  |  |  |  |  |  |  |  | Reduces bad breath | 5 | 14 | 1 |  |
|  |  |  |  |  |  | Latex | Collect the latex by cutting the bark | Apply to a mildly bleeding wound | Hemostatic | 1 | 1,2,14,30 | 4 |  |
|  |  |  |  |  |  |  |  |  | Healing | 1 | 1,2,30 | 2 |  |
|  |  |  |  |  |  | Seed | Take the seed and a piece of wood or a skewer. Pierce the seed with it and place it over the fire until it turns into charcoal. Remove the skewer and let it cool on a banana leaf softened over the fire. Crush it into powder and put it in a 30 ml perfume bottle, such as Pompeia. Add a little water and let it sit for a week. | Apply on eyes | Make up | 5 | 27 | 1 |  |
| *Fabaceae* | *Crotalaria laburnoides* Klotzsch | Mimosa pudique | ND | ND | Ampamono maso na koho bory |  |  |  |  |  |  |  | NC |
|  |  |  |  |  |  | Pod | Crush the pods and add water | Hair | Shampoo | 1 | 19 | 1 |  |
| *Fabaceae* | *Hymenaea verrucosa* Gaertn. | Copalier | gum copal tree | Mvoumba | Yembuki |  |  |  |  |  |  |  | NC |
|  |  |  |  |  |  | Bark | Scrape the bark on the dead coral stone with a little water until you obtain a smooth paste | Face | Anti pimples | 1 | 25 | 1 |  |
|  |  |  |  |  |  |  |  |  | Brightens (face) | 3 | 25 | 1 |  |
|  |  |  |  |  |  |  |  |  | Beautifies (Face) | 5 | 25 | 1 |  |
| *Fabaceae* | *Indigofera tinctoria* L. | Indigotier | Indigo | M'komba unyo | Heinguitshi |  |  |  |  |  |  |  | ODAM014 |
|  |  |  |  |  |  | Leaf | Crush the leaves in your hands until sweating occurs | Rub under the armpits | Antiperspirant | 1 | 12,18 | 2 |  |
|  |  |  |  |  |  |  |  |  | Deodorant | 1 | 12 | 1 |  |
|  |  |  |  |  |  |  | ND | Body / skin | Anti pimples | 1 | 3 | 1 |  |
| *Fabaceae* | *Mimosa pudica*  L. | Sensitive | Sensitive plant | ND | Fatsiki ambili, Shibalabala masu |  |  |  |  |  |  |  | ODAM045 |
|  |  |  |  |  |  | Leaf | Crush the leaves of "fatsiki ambili" with salt | Apply a poultice around the swollen boil (furuncle) and other fingers and hands using the leaves of fatsiki ambili crushed with salt. Leave it until the boil bursts | Boils. Attention, intense irritation and tightening of the affected area | 1 | 29 | 1 |  |
| *Fabaceae* | *Pterocarpus indicus* Willd. | Sang-dragon | Angsana tree |  | M'sandrago |  |  |  |  |  |  |  | NC |
|  |  |  |  |  |  | Leaf | Crush leaves on water | Hair | Shampoo | 4 | 6,9,11 | 3 |  |
|  |  |  |  |  |  |  | Place the leaves of *Pterocarpus indicus* in *Cocos nucifera* oil | Hair and style | ND | 4 | 9,11 | 2 |  |
| *Fabaceae* | *Senna singueana* (Delile) Lock |  | Winter cassia | M'ri m'buzi | Andra béyi, Sambaravatsi |  |  |  |  |  |  |  | ODAM044 |
|  |  |  |  |  |  | Leaf | Mix the leaves of "Sambaravatsi" with *Cocos nucifera* oil | Wash and apply to the affected area. | Allergies causing pinkish patches | 1 | 28 | 1 |  |
|  |  |  |  |  |  |  | Crush the leaves of *Senna singueana* and mix with *Cocos nucifera* oil | Apply to the affected area | Dermatoses | 1 | 35 | 1 |  |
| *Fabaceae* | *Vachellia farnesiana* (L.) Wight & Arn. | Acacia de farnèse | Sweet acacia | Mugu dzidzano | Fu mgu tamotamo |  |  |  |  |  |  |  | ODAM 001 |
|  |  |  |  |  |  | Flower | Not preparation recquired | Place a mixture of *Rosa alba* and *Jasminum nummulariaefolium* flowers, along with *Vachellia farnesiana*, on the bed | Fragrance | 5 | 11 | 1 |  |
|  |  |  |  |  |  |  |  | Arrange on the bed a mixture of J*asminum nummulariaefolium* and *Vachellia farnesiana* flowers | Fragrance | 5 | 11 | 1 |  |
|  |  |  |  |  |  |  |  | Arrange on the bed a mixture of *Rosa alba* and J*asminum nummulariaefolium* flowers, along with *Vachellia farnesiana* and leaves and inflorescences of *Ocimum* spp. | Fragrance | 5 | 11 | 1 |  |
|  |  |  |  |  |  |  | Crush the leaves and inflorescences of *Ocimum* spp., the rhizome of *Curcuma longa*, the roots of *Chrysopogon zizanoides*, the flowers of *Rosa alba*, *Pandanus maximus, Jasminum nummulariaefolium,* and *Vachellia farnesiana*. Mix everything with the milk and grated fruit of *Cocos nucifera*. Let it macerate for a few minutes or even hours | Exfoliation | Skin care | 2 | 5 | 1 |  |
|  |  |  |  |  |  |  |  |  | Cleanser | 5 | 5 | 1 |  |
|  |  |  |  |  |  |  |  |  | Softens | 2 | 5 | 1 |  |
|  |  |  |  |  |  |  |  |  | Nourishes | 2 | 5 | 1 |  |
|  |  |  |  |  |  |  |  |  | Beautifies | 5 | 5 | 1 |  |
|  |  |  |  |  |  |  |  |  | Hydrates | 2 | 5 | 1 |  |
|  |  |  |  |  |  |  | Create a mixture of *Jasminum nummulariaefolium*, *Rosa alba*, and *Vachellia farnesiana* flowers, and place them on safety pins. | Hair | Beautifies | 5 | 16 | 1 |  |
|  |  |  |  |  |  |  | Create a mixture of *Jasminum nummulariaefolium*, *Rosa alba*, and *Vachellia farnesiana* flowers, and place them on neckelace (toka). | Neck | Beautifies | 5 | 16 | 1 |  |
|  |  |  |  |  |  |  | The flowers of *Vachellia farnesiana* are placed on safety pins | Hair | Beautifies | 5 | 14 | 1 |  |
|  |  |  |  |  |  |  | The flowers of *Vachellia farnesiana* are placed on safety pins |  | Fragrance | 5 | 14 | 1 |  |
|  |  |  |  |  |  |  | The flowers of *Vachellia farnesiana* are placed on necklace | Neck | Beautifies | 5 | 14 | 1 |  |
|  |  |  |  |  |  |  | The flowers of *Vachellia farnesiana* are placed on necklace |  | Fragrance | 5 | 14 | 1 |  |
|  |  |  |  |  |  |  | Mix the flowers of *Vachellia farnesiana* and *Cananga odorata*, and place them on safety pins | Hair | Beautifies | 5 | 17 | 1 |  |
|  |  |  |  |  |  |  | Mix the flowers of *Vachellia farnesiana* and *Cananga odorata*, and place them on necklace | Neck | Beautifies | 5 | 17 | 1 |  |
|  |  |  |  |  |  |  | String a combination of *Jasminum nummulariaefolium* and *Vachellia farnesiana* flowers onto a necklace | Neck | Beautifies | 5 | 26 | 1 |  |
|  |  |  |  |  |  |  | String a combination of *Jasminum nummulariaefolium* and *Vachellia farnesiana* flowers, along with leaves and inflorescences of *Ocimum spp.*, onto necklaces | Neck | Beautifies | 5 | 27 | 1 |  |
|  |  |  |  |  |  |  | Pierce the flowers with the help of a safety pin and form a heap on it by making mixtures to taste with jasmine flowers, farnese acacia, grand vacoa, or leaves and inflorescences of basil | Hair | Beautifies | 5 | 4 | 1 |  |
|  |  |  |  |  |  |  |  |  | Fragrance | 5 | 4 | 1 |  |
|  |  |  |  |  |  |  | Pierce the flowers with a safety pin and form a heap on it | Hair | Beautifies | 5 | 4 | 1 |  |
|  |  |  |  |  |  |  |  |  | Fragrance | 5 | 4 | 1 |  |
|  |  |  |  |  |  |  | Place on the kilabou the leaves and inflorescences of *Ocimum* spp., mixing them with flowers of *Jasminum nummulariaefolium* and *Vachellia farnesiana* | Hair | Beautifies | 5 | 5,27,32 | 3 |  |
|  |  |  |  |  |  |  |  |  | Fragrance | 5 | 5,32 | 2 |  |
|  |  |  |  |  |  |  |  | Place on the chest | Beautifies | 5 | 5,27,32 | 3 |  |
|  |  |  |  |  |  |  |  |  | Fragrance | 5 | 5,32 | 2 |  |
|  |  |  |  |  |  |  | Pierce the flowers as per availability and preference of *Jasminum nummulariaefolium, Pandanus maximus, Vachellia farnesiana, Canaga odorata, Rosa alba*, "Koukoumba", *Cestrum nocturnum* using a safety pin and form a heap on it | Hair | Beautifies | 5 | 7 | 1 |  |
|  |  |  |  |  |  |  |  |  | Fragrance | 5 | 7 | 1 |  |
|  |  |  |  |  |  |  | Place on safety pins a mixture of *Jasminum nummulariaefolium* and *Vachellia farnesiana* flowers | Hair | Beautifies | 5 | 9,21,26,11 | 4 |  |
|  |  |  |  |  |  |  |  |  | Fragrance | 5 | 9,21,11 | 3 |  |
|  |  |  |  |  |  |  |  | Clothing | Beautifies | 5 | 26,11 | 2 |  |
|  |  |  |  |  |  |  |  |  | Fragrance | 5 | 11 | 1 |  |
|  |  |  |  |  |  |  | Place a mixture of *Jasminum nummulariaefolium, Vachellia farnesiana, Rosa alba* flowers, and leaves and inflorescences of *Ocimum* spp. on safety pins | Hair | Beautifies | 5 | 10,11 | 2 |  |
|  |  |  |  |  |  |  |  |  | Fragrance | 5 | 10,11 | 2 |  |
|  |  |  |  |  |  |  |  | Clothing | Beautifies | 5 | 11 | 1 |  |
|  |  |  |  |  |  |  |  |  | Fragrance | 5 | 11 | 1 |  |
|  |  |  |  |  |  |  | Place a mixture of *Rosa alba*, *Jasminum nummulariaefolium*, and *Vachellia farnesiana* flowers on safety pins | Hair | Beautifies | 5 | 11 | 1 |  |
|  |  |  |  |  |  |  |  |  | Fragrance | 5 | 11 | 1 |  |
|  |  |  |  |  |  |  |  | Clothing | Beautifies | 5 | 11 | 1 |  |
|  |  |  |  |  |  |  |  |  | Fragrance | 5 | 11 | 1 |  |
|  |  |  |  |  |  |  | Take the mixture of plants obtained from marriages or create your own blend based on plant availability (Roots of *Chrysopogon zizanoides,* Leaves of *Ayapana triplinervis,* Leaves and inflorescences of *Ocimum* spp., Flowers of *Jasminum nummulariaefolium, Vachellia farnesiana, Rosa alba*, "Koukoumba", *Plumeria alba, Cananga odorata, Pandanus maximus*). Once dried, crush and sort them by size. Prepare the coconut oil. To do this, grate the coconuts, extract the milk, let it sit overnight, remove the cream on top, and then cook it in a large pot over a wood fire. Once the oil is ready, add the flower mixture "zoukouba" when the temperature has cooled slightly, and add the pompeia when the oil is no longer very hot. Let it all macerate. The longer the maceration, the more intense the fragrance will be | Body / skin | Nourishes | 2 | 6 | 1 |  |
|  |  |  |  |  |  |  |  |  | Add shine | 3 | 6 | 1 |  |
|  |  |  |  |  |  |  |  |  | Softens | 2 | 6 | 1 |  |
|  |  |  |  |  |  |  |  |  | Fragrans | 5 | 6 | 1 |  |
|  |  |  |  |  |  |  |  |  | Lightens | 3 | 6 | 1 |  |
|  |  |  |  |  |  |  |  |  | Hydrates | 2 | 6 | 1 |  |
|  |  |  |  |  |  |  | Not preparation recquired | Place flower of *Vachellia farnesiana* on bed | Fragrance | 5 | 4,4,5,7,14,32 | 6 |  |
| *Lamiaceae* | *Ocimum americanum* L. | Basilic citron | American basil | ND | Karandzani mroni |  |  |  |  |  |  |  | ODAM046 |
|  |  |  |  |  |  | Leaf | Not preparation recquired | Rub the leaves under the armpits | Antiperspirant | 1 | 15 | 1 |  |
|  |  |  |  |  |  |  |  |  | Deodorant | 1 | 15 | 1 |  |
| *Lamiaceae* | *Ocimum gratissimum* L. | Basilic grande feuille | African basil | M'rule | Rulé |  |  |  |  |  |  |  | ODAM005 |
|  |  |  |  |  |  | Leaf | Decoction | Private parts | Cleanser | 5 | 3,8,13 | 3 |  |
|  |  |  |  |  |  |  |  |  | Reduces vaginal odors | 5 | 3,13,15 | 3 |  |
|  |  |  |  |  |  |  |  |  | Reduces white discharge | 5 | 3,13 | 2 |  |
|  |  |  |  |  |  |  |  |  | Tightens the vagina | 5 | 8,13 | 2 |  |
|  |  |  |  |  |  |  |  |  | Maintaining the vagina | 5 | 2,8 | 2 |  |
|  |  |  |  |  |  |  | Crush the leaves of *Ocimum gratissimum, Lantana trifolia*, and the rhizome of *Zingiber officinale*, and form a ball | Insert the ball into the vagina and sit over a heated stone | Tightens the vagina | 5 | 15 | 1 |  |
|  |  |  |  |  |  |  |  |  | Tones the vagina | 5 | 15 | 1 |  |
|  |  |  |  |  |  |  | Take the leaves, crush them, and put them in salted water | Private parts | Cleanser | 5 | 17 | 1 |  |
|  |  |  |  |  |  |  |  |  | Reduces vaginal odors | 5 | 17 | 1 |  |
|  |  |  |  |  |  |  |  |  | Reduces white discharge | 5 | 17 | 1 |  |
|  |  |  |  |  |  |  |  |  | Tightens the vagina | 5 | 17 | 1 |  |
| *Lamiaceae* | *Ocimum* spp. | ND | ND | Mkadi | Mkadi |  |  |  |  |  |  |  | *ODAM007* |
|  |  |  |  |  |  | Leaf and inflorescence | Not preparation recquired | Arrange on the bed a mixture of *Jasminum nummulariaefolium* flowers and leaves and inflorescences of *Ocimum* spp. | Fragrance | 5 | 11 | 1 |  |
|  |  |  |  |  |  |  |  | Arrange on the bed a mixture of *Rosa alba* and J*asminum nummulariaefolium* flowers, along with *Vachellia farnesiana* and leaves and inflorescences of *Ocimum* spp. | Fragrance | 5 | 11 | 1 |  |
|  |  |  |  |  |  |  |  | Arrange on the bed a mixture of J*asminum nummulariaefolium flowers, Pandanus maximus*, along with leaves and inflorescences of *Ocimum* spp. | Fragrance | 5 | 12 | 1 |  |
|  |  |  |  |  |  |  |  | Arrange on the bed a mixture of leaves and inflorescences of *Ocimum* spp., flowers of *Pandanus maximus, Jasminum nummulariaefolium*, and *Cananga odorata* | Beautifies | 5 | 23 | 1 |  |
|  |  |  |  |  |  |  |  |  | Fragrance | 5 | 23 | 1 |  |
|  |  |  |  |  |  |  | Crush the leaves and inflorescences of *Ocimum* spp., the rhizome of *Curcuma longa*, the roots of *Chrysopogon zizanoides*, the flowers of *Rosa alba, Pandanus maximus, Jasminum nummulariaefolium*, and *Vachellia farnesiana*. Mix everything with the milk and grated fruit of *Cocos nucifera*. Let it macerate for a few minutes or even hours | Exfoliation | Skin care | 2 | 5 | 1 |  |
|  |  |  |  |  |  |  |  |  | Cleanser | 5 | 5 | 1 |  |
|  |  |  |  |  |  |  |  |  | Softens | 2 | 5 | 1 |  |
|  |  |  |  |  |  |  |  |  | Nourishes | 2 | 5 | 1 |  |
|  |  |  |  |  |  |  |  |  | Beautifies | 5 | 5 | 1 |  |
|  |  |  |  |  |  |  |  |  | Hydrates | 2 | 5 | 1 |  |
|  |  |  |  |  |  |  | Create a necklace with a mixture of *Jasminum nummulariaefolium, Plumeria alba, Cananga odorata, Bougainvillea spectabilis* flowers, and leaves of *Pogostemon cablin*, as well as leaves and inflorescences of *Ocimum* spp. | Neck | Fragrance | 5 | 11 | 1 |  |
|  |  |  |  |  |  |  |  |  | Beautifies | 5 | 11 | 1 |  |
|  |  |  |  |  |  |  | Create a mixture of Jasminum nummulariaefolium flowers and leaves and inflorescences of Ocimum spp. and place them on safety pins. | Hair | Beautifies | 5 | 16 | 1 |  |
|  |  |  |  |  |  |  | Create a mixture of Jasminum nummulariaefolium flowers and leaves and inflorescences of Ocimum spp. and place them on necklace. | Neck | Beautifies | 5 | 16 | 1 |  |
|  |  |  |  |  |  |  | The leaves and inflorescences are placed on safety pins | Hair | Beautifies | 5 | 14 | 1 |  |
|  |  |  |  |  |  |  |  |  | Fragrance | 5 | 1,14 | 2 |  |
|  |  |  |  |  |  |  |  | Clothing | Beautifies | 5 | 14 | 1 |  |
|  |  |  |  |  |  |  |  |  | Fragrance | 5 | 14 | 1 |  |
|  |  |  |  |  |  |  | The leaves and inflorescences are placed on necklace | Neck | Beautifies | 5 | 14 | 1 |  |
|  |  |  |  |  |  |  |  |  | Fragrance | 5 | 14 | 1 |  |
|  |  |  |  |  |  |  | Mix the leaves and inflorescences of *Ocimum* spp. with the flowers of *Pandanus maximus, Rosa alba, Jasminum nummulariaefolium, Cananga odorata*, and the roots of *Chrysopogon zizanoïdes*. Incorporate everything into *Cocos nucifera* oil and let it macerate | Body / skin | Beautifies | 5 | 4 | 1 |  |
|  |  |  |  |  |  |  |  | Face | Beautifies | 5 | 4 | 1 |  |
|  |  |  |  |  |  |  |  | Body / skin | Fragrance | 5 | 4 | 1 |  |
|  |  |  |  |  |  |  |  | Face | Fragrance | 5 | 4 | 1 |  |
|  |  |  |  |  |  |  | Mix all the following plants: roots of *Chrysopogon zizanoides*, leaves and inflorescences of *Ocimum* spp., leaves of *Ayapana triplinervis*, flowers of *Jasminum nummulariaefolium, Pandanus maximus, Cananga odorata*; extracted from safety pins. Introduce them into *Cocos nucifera* oil and let them macerate in the oil | Body / skin | Beautifies | 5 | 5 | 1 |  |
|  |  |  |  |  |  |  |  |  | Fragrance | 5 | 5 | 1 |  |
|  |  |  |  |  |  |  |  |  | Softens | 2 | 5 | 1 |  |
|  |  |  |  |  |  |  |  |  | Post-depilatory care | 4 | 5 | 1 |  |
|  |  |  |  |  |  |  | Place the leaves and inflorescences of *Ocimum* spp. in *Cocos nucifera* oil and let it macerate for several weeks | Body / skin | Fragrance | 5 | 7 | 1 |  |
|  |  |  |  |  |  |  |  |  | Hydrates | 2 | 7 | 1 |  |
|  |  |  |  |  |  |  |  |  | Protect | 5 | 7 | 1 |  |
|  |  |  |  |  |  |  |  | Face | Fragrance | 5 | 7 | 1 |  |
|  |  |  |  |  |  |  |  |  | Hydrates | 2 | 7 | 1 |  |
|  |  |  |  |  |  |  |  |  | Protect | 5 | 7 | 1 |  |
|  |  |  |  |  |  |  | Infuse the dried plants, including flowers of *Jasminum nummulariaefolium, Pandanus maximus, Plumeria alba, Cananga odorata, Rosa alba,* roots of *Chrysopogon zizanoides*, leaves of *Pogostemon cablin, Ayapana triplinervis*, and leaves and inflorescence of *Ocimum* spp., in *Cocos nucifera* oil | Body / skin | Fragrance | 5 | 10 | 1 |  |
|  |  |  |  |  |  |  | Mix the dried flowers of *Pandanus maximus, Jasminum nummulariaefolium, Cananga odorata,* the dried leaves of *Pogostemon cablin, Ayapana triplinervis,* the dried leaves and inflorescences of *Ocimum* spp., and the root of *Chrysopogon zizanoides* | Body / skin | Fragrance | 5 | 11 | 1 |  |
|  |  |  |  |  |  |  | Mix the flowers of *Pandanus maximus* and the leaves and inflorescences of *Ocimum* spp., and place them on safety pins | Hair | Beautifies | 5 | 18 | 1 |  |
|  |  |  |  |  |  |  | Mix the flowers of *Pandanus maximus* and the leaves and inflorescences of *Ocimum* spp., and place them on necklace | Neck | Beautifies | 5 | 18 | 1 |  |
|  |  |  |  |  |  |  | Put the dried leaves and inflorescences of *Ocimum* spp., the dried roots of *Chrysopogon zizanoides*, the dried flowers of *Pandanus maximus*, and *Jasminum nummulariaefolium* in *Cocos nucifera* milk, and add the grated fruit | Massage | Skin care | 2 | 23 | 1 |  |
|  |  |  |  |  |  |  |  |  | Cleanser | 5 | 23 | 1 |  |
|  |  |  |  |  |  |  |  |  | Softens | 2 | 23 | 1 |  |
|  |  |  |  |  |  |  |  |  | Nourishes | 2 | 23 | 1 |  |
|  |  |  |  |  |  |  |  |  | Beautifies | 5 | 23 | 1 |  |
|  |  |  |  |  |  |  |  |  | Hydrates | 2 | 23 | 1 |  |
|  |  |  |  |  |  |  |  |  | Fragrance | 5 | 23 | 1 |  |
|  |  |  |  |  |  |  | Put on safety pins | Hair | Beautifies | 5 | 33 | 1 |  |
|  |  |  |  |  |  |  |  |  | Fragrance | 5 | 33 | 1 |  |
|  |  |  |  |  |  |  |  | Clothing | Beautifies | 5 | 33 | 1 |  |
|  |  |  |  |  |  |  |  |  | Fragrance | 5 | 33 | 1 |  |
|  |  |  |  |  |  |  | Place the leaves and inflorescences of *Ocimum* spp. on the kilabou, mixing them with flowers of *Jasminum nummulariaefolium* | Hair | Beautifies | 5 | 5 | 1 |  |
|  |  |  |  |  |  |  |  |  | Fragrance | 5 | 5 | 1 |  |
|  |  |  |  |  |  |  |  | Torso | Beautifies | 5 | 5 | 1 |  |
|  |  |  |  |  |  |  |  |  | Fragrance | 5 | 5 | 1 |  |
|  |  |  |  |  |  |  | Place the leaves and inflorescences of Ocimum spp. on the kilabou, mixing them with flowers of Jasminum nummulariaefolium and Rosa alba | Hair | Beautifies | 5 | 5 | 1 |  |
|  |  |  |  |  |  |  |  |  | Fragrance | 5 | 5 | 1 |  |
|  |  |  |  |  |  |  |  | Torso | Beautifies | 5 | 5 | 1 |  |
|  |  |  |  |  |  |  |  |  | Fragrance | 5 | 5 | 1 |  |
|  |  |  |  |  |  |  | Place the leaves and inflorescences of Ocimum spp. on the kilabou, mixing them with flowers of Jasminum nummulariaefolium and Vachellia farnesiana | Hair | Beautifies | 5 | 5 | 1 |  |
|  |  |  |  |  |  |  |  |  | Fragrance | 5 | 5 | 1 |  |
|  |  |  |  |  |  |  |  | Torso | Beautifies | 5 | 5 | 1 |  |
|  |  |  |  |  |  |  |  |  | Fragrance | 5 | 5 | 1 |  |
|  |  |  |  |  |  |  | Place the leaves and inflorescences of Ocimum spp. on the kilabou, mixing them with flowers of Pandanus maximus | Hair | Beautifies | 5 | 5 | 1 |  |
|  |  |  |  |  |  |  |  |  | Fragrance | 5 | 5 | 1 |  |
|  |  |  |  |  |  |  |  | Torso | Beautifies | 5 | 5 | 1 |  |
|  |  |  |  |  |  |  |  |  | Fragrance | 5 | 5 | 1 |  |
|  |  |  |  |  |  |  | Mix all the following plants: roots of Chrysopogon zizanoides, leaves and inflorescences of Ocimum spp., leaves of Ayapana triplinervis, flowers of Jasminum nummulariaefolium, Pandanus maximus, Cananga odorata; extracted from safety pins. Introduce them into Cocos nucifera oil and let them macerate in the oil | Body / skin | Beautifies | 5 | 5 | 1 |  |
|  |  |  |  |  |  |  |  |  | Fragrance | 5 | 5 | 1 |  |
|  |  |  |  |  |  |  |  |  | Softens | 2 | 5 | 1 |  |
|  |  |  |  |  |  |  |  |  | Post-depilatory care | 4 | 5 | 1 |  |
|  |  |  |  |  |  |  | Place the leaves and inflorescences of *Ocimum* spp. on safety pins, mixed with flowers of *Pandanus maximus* | Hair | Beautifies | 5 | 9,10, | 2 |  |
|  |  |  |  |  |  |  |  |  | Fragrance | 5 | 9 | 1 |  |
|  |  |  |  |  |  |  | Place on safety pins a mixture of flowers from *Jasminum nummulariaefolium, Vachellia farnesiana, Rosa alba,* and leaves and inflorescences of *Ocimum* spp. | Hair | Beautifies | 5 | 10 | 1 |  |
|  |  |  |  |  |  |  |  |  | Fragrance | 5 | 10 | 1 |  |
|  |  |  |  |  |  |  | Place on safety pins a mixture of flowers from J*asminum nummulariaefolium* and leaves and inflorescences of *Ocimum* spp. | Hair | Beautifies | 5 | 11 | 1 |  |
|  |  |  |  |  |  |  |  |  | Fragrance | 5 | 11 | 1 |  |
|  |  |  |  |  |  |  |  | Clothing | Beautifies | 5 | 11 | 1 |  |
|  |  |  |  |  |  |  |  |  | Fragrance | 5 | 11 | 1 |  |
|  |  |  |  |  |  |  | Place on safety pins a mixture of flowers from R*osa alba* and *Jasminum nummulariaefolium, Vachellia farnesiana*, with leaves and inflorescences of *Ocimum* spp. | Hair | Beautifies | 5 | 11 | 1 |  |
|  |  |  |  |  |  |  |  |  | Fragrance | 5 | 11 | 1 |  |
|  |  |  |  |  |  |  |  | Clothing | Beautifies | 5 | 11 | 1 |  |
|  |  |  |  |  |  |  |  |  | Fragrance | 5 | 11 | 1 |  |
|  |  |  |  |  |  |  | Place on safety pins a mixture of flowers from *Jasminum nummulariaefolium, Pandanus maximus,* with leaves and inflorescences of *Ocimum* spp. | Hair | Beautifies | 5 | 12 | 1 |  |
|  |  |  |  |  |  |  |  |  | Fragrance | 5 | 12 | 1 |  |
|  |  |  |  |  |  |  |  | Clothing | Beautifies | 5 | 12 | 1 |  |
|  |  |  |  |  |  |  |  |  | Fragrance | 5 | 12 | 1 |  |
|  |  |  |  |  |  |  | Place a mixture of leaves and inflorescences of *Ocimum* spp., the flowers of *Pandanus maximus, Jasminum nummulariaefolium,* and *Cananga odorata* on the kilabou. | Hair | Beautifies | 5 | 23 | 1 |  |
|  |  |  |  |  |  |  |  |  | Fragrance | 5 | 23 | 1 |  |
|  |  |  |  |  |  |  | Place on safety pins a mixture of flowers from *Jasminum nummulariaefolium, Vachellia farnesiana*, and leaves and inflorescences of *Ocimum* spp. | Hair | Beautifies | 5 | 27 | 1 |  |
|  |  |  |  |  |  |  |  | Clothing | Beautifies | 5 | 27 | 1 |  |
|  |  |  |  |  |  |  | Place on snecklace pins a mixture of flowers from *Jasminum nummulariaefolium, Vachellia farnesiana*, and leaves and inflorescences of *Ocimum* spp. | Neck | Beautifies | 5 | 27 | 1 |  |
|  |  |  |  |  |  |  | Place on safety pins a mixture of leaves and inflorescences of *Ocimum* spp. and *Jasminum nummulariaefolium* | Hair | Fragrance | 5 | 32 | 1 |  |
|  |  |  |  |  |  |  |  |  | Beautifies | 5 | 32 | 1 |  |
|  |  |  |  |  |  |  |  | Clothing | Fragrance | 5 | 32 | 1 |  |
|  |  |  |  |  |  |  |  |  | Beautifies | 5 | 32 | 1 |  |
|  |  |  |  |  |  |  | Place on necklace pins a mixture of leaves and inflorescences of Ocimum spp. and Jasminum nummulariaefolium | Neck | Fragrance | 5 | 32 | 1 |  |
|  |  |  |  |  |  |  |  |  | Beautifies | 5 | 32 | 1 |  |
|  |  |  |  |  |  |  | Put on necklace | Neck | Fragrance | 5 | 33 | 1 |  |
|  |  |  |  |  |  |  |  |  | Beautifies | 5 | 33 | 1 |  |
|  |  |  |  |  |  |  | Pierce the flowers of *Pandanus maximus* with a needle and fill a thread with rice bag. Tie it all together *Ocimum sp* and *Pandanus maximus*. Used for making a necklace. | Neck | Beautifies | 5 | 3 | 1 |  |
|  |  |  |  |  |  |  | Pierce the leaves and inflorescences with a safety pin and form a pile on it. | Hair | Beautifies | 5 | 3,4 | 2 |  |
|  |  |  |  |  |  |  |  |  | Fragrance | 5 | 4 | 1 |  |
|  |  |  |  |  |  |  |  | Clothing | Beautifies | 5 | 3 | 1 |  |
|  |  |  |  |  |  |  | Pierce the flowers with a safety pin and form a pile on it, mixing to taste with jasmine flowers, farnese acacia, grand vacoa, or leaves and inflorescences of basil | Hair | Beautifies | 5 | 4 | 1 |  |
|  |  |  |  |  |  |  |  |  | Fragrance | 5 | 4 | 1 |  |
|  |  |  |  |  |  |  | Pierce the leaves and inflorescences of *Ocimum* spp. with "koukoumba" flowers using a safety pin and form a pile on it | Hair | Beautifies | 5 | 4 | 1 |  |
|  |  |  |  |  |  |  |  |  | Fragrance | 5 | 4 | 1 |  |
|  |  |  |  |  |  |  | Take the mixture of plants obtained from marriages or create your own blend based on plant availability (Roots of *Chrysopogon zizanoides,* Leaves of *Ayapana triplinervis,* Leaves and inflorescences of *Ocimum* spp., Flowers of *Jasminum nummulariaefolium, Vachellia farnesiana, Rosa alba*, "Koukoumba", *Plumeria alba, Cananga odorata, Pandanus maximus*). Once dried, crush and sort them by size. Prepare the coconut oil. To do this, grate the coconuts, extract the milk, let it sit overnight, remove the cream on top, and then cook it in a large pot over a wood fire. Once the oil is ready, add the flower mixture "zoukouba" when the temperature has cooled slightly, and add the pompeia when the oil is no longer very hot. Let it all macerate. The longer the maceration, the more intense the fragrance will be | Body / skin | Nourishes | 2 | 6 | 1 |  |
|  |  |  |  |  |  |  |  |  | Add shine | 3 | 6 | 1 |  |
|  |  |  |  |  |  |  |  |  | Softens | 2 | 6 | 1 |  |
|  |  |  |  |  |  |  |  |  | Fragrans | 5 | 6 | 1 |  |
|  |  |  |  |  |  |  |  |  | Lightens | 3 | 6 | 1 |  |
|  |  |  |  |  |  |  |  |  | Hydrates | 2 | 6 | 1 |  |
|  |  |  |  |  |  |  | Take the leaves and inflorescences of Ocimum spp. and wrap them with Pandanus maximus flowers, securing everything with threads from a rice bag using a needle | Hair | Beautifies | 5 | 12 | 1 |  |
|  |  |  |  |  |  |  |  |  | Attract luck | 5 | 12 | 1 |  |
|  |  |  |  |  |  |  |  |  | Fragrance | 5 | 12 | 1 |  |
|  |  |  |  |  |  |  |  | Bed | Fragrance | 5 | 12 | 1 |  |
| *Lamiaceae* | *Plectranthus amboinicus* (Lour.) Spreng. | Gros thym | Cuban oregano | Porouvi | Parauvi |  |  |  |  |  |  |  | NC |
|  |  |  |  |  |  | Leaf | Crush the leaves and add sugar | Apply the crushed leaves and sugar mixture on small to medium open wounds as a poultice | Promotes healing | 1 | 12 | 1 |  |
|  |  |  |  |  |  |  |  |  | Wound healing | 1 | 12 | 1 |  |
|  |  |  |  |  |  |  | Crush the leaves and add salt | Apply to wounds caused by burns. | Wound healing | 1 | 30 | 1 |  |
|  |  |  |  |  |  |  |  |  |  |  |  |  |  |
|  |  |  |  |  |  |  |  |  |  |  |  |  |  |
|  |  |  |  |  |  |  |  |  |  |  |  |  |  |
| *Lamiaceae* | *Pogostemon cablin* Benth. | Patchoulie | Patchouli | Patchor | Patchor |  |  |  |  |  |  |  | *ODAM008* |
|  |  |  |  |  |  | Leaf | Put the dried plants, including flowers of *Jasminum nummulariaefolium, Pandanus maximus, Plumeria alba, Cananga odorata, Rosa alba*, the roots of *Chrysopogon zizanoides*, the leaves of *Pogostemon cablin, Ayapana triplinervis,* and the leaves and inflorescence of *Ocimum* spp., in a decoction with *Cocos nucifera* oil | Body / skin | Fragrance | 5 | 10 | 1 |  |
|  |  |  |  |  |  |  | Mix the dried flowers of *Pandanus maximus, Jasminum nummulariaefolium, Cananga odorata*, the dried leaves of *Pogostemon cablin, Ayapana triplinervis*, and the dried leaves and inflorescences of *Ocimum* spp., along with the root of *Chrysopogon zizanoides* | Body / skin | Fragrance | 5 | 11 | 1 |  |
|  |  |  |  |  |  |  | Create a necklace with a mixture of flowers from *Jasminum nummulariaefolium, Plumeria alba, Cananga odorata, Bougainvillea spectabilis,* and leaves of *Pogostemon cablin*, as well as leaves and inflorescences of *Ocimum* spp. | Neck | Beautifies | 5 | 11 | 1 |  |
|  |  |  |  |  |  |  |  |  | Fragrance | 5 | 11 | 1 |  |
|  |  |  |  |  |  |  | Put on safety pins a mixture of *Jasminum nummulariaefolium* flowers and *Pogostemon cablin* leaves | Hair | Beautifies | 5 | 11 | 1 |  |
|  |  |  |  |  |  |  |  |  | Fragrance | 5 | 11 | 1 |  |
|  |  |  |  |  |  |  |  | Clothing | Beautifies | 5 | 11 | 1 |  |
|  |  |  |  |  |  |  |  |  | Fragrance | 5 | 11 | 1 |  |
|  |  |  |  |  |  |  | Place on the bed a mixture of Jasminum nummulariaefolium flowers and Pogostemon cablin leaves | Bed | Fragrance | 5 | 11 | 1 |  |
| *Lauraceae* | *Cassytha filiformis* L. | Liane Foutafout | Bush-dodder | ND | Tsihitafotoutshou |  |  |  |  |  |  |  | NC |
|  |  |  |  |  |  | Whole plant | Grind the plant | Body / skin | Dermatoses | 1 | 31 | 1 |  |
| *Lauraceae* | *Listea glutinosa* (Lour.) C.B.Robins | Bois d'oiseaux à petites feuille | Bolly-beech | M'zavoca maro | Zavoca maro |  |  |  |  |  |  |  | ODAM002 |
|  |  |  |  |  |  | Sap | Scrape the stem and collect the sap | Apply on small wounds and cuts without excessive bleeding | Promotes healing | 1 | 2 | 1 |  |
|  |  |  |  |  |  |  |  | Apply on wounds, injuries, or burns | Promotes healing | 1 | 13 | 1 |  |
|  |  |  |  |  |  |  |  |  | Promotes burns healing | 1 | 13 | 1 |  |
| *Lauraceae* | *Persea americana* Mill. | Avocatier | Avocado | Avoka | Avoka |  |  |  |  |  |  |  | ODAM047 |
|  |  |  |  |  |  | Seed | Grind the seed on a dead coral stone with a little water until obtaining a smooth paste | Face | Beauty mask | 2 | 4 | 1 |  |
|  |  |  |  |  |  |  |  |  | Hydrates | 2 | 4 | 1 |  |
|  |  |  |  |  |  |  |  |  | Beautifies | 5 | 4,16,32 | 3 |  |
|  |  |  |  |  |  |  |  |  | Anti pimples | 1 | 4,16,32 | 3 |  |
|  |  |  |  |  |  |  |  |  | Anti-aging | 2 | 14,32 | 2 |  |
|  |  |  |  |  |  |  |  | Massage (Body / skin) | Cleanser | 5 | 14 | 1 |  |
|  |  |  |  |  |  |  |  |  | Beautifies | 5 | 14 | 1 |  |
| *Loganiaceae* | *Strychnos spinosa* Lam. | Oranger du Natal | Natal orange | ND | Mkutsha |  |  |  |  |  |  |  | ODAM048 |
|  |  |  |  |  |  | Root | Decoction | Body / skin (baby born) | Skin condition | 1 | 30 | 1 |  |
| *Lythraceae* | *Lawsonia inermis* L. | Hénné | Henna | Hina dzishe | Mwina vavy |  |  |  |  |  |  |  | ODAM013 |
|  |  |  |  |  |  | Leaf | Crush leaves of *Lawsonia inermis* and add lemon juice *Citrus* sp. | Apply on the soles of the feet and heels | Heal the cracks | 4 | 1,1,2,2,13,13,15,15,32,32 | 10 |  |
|  |  |  |  |  |  |  |  | Massage (face). Attention : not to go in the sun and be careful not to leave it on the face as it may cause discoloration. Avoid the eye area due to the presence of lemon | Cleanser | 5 | 9 | 1 |  |
|  |  |  |  |  |  |  |  |  | Tanned complexion | 3 | 9 | 1 |  |
|  |  |  |  |  |  |  |  | Apply on hands and feet. Leave for a few hours. Then rinse | Beautifies | 4 | 10 | 1 |  |
|  |  |  |  |  |  |  |  | Apply on the skin and leave it on for a few minutes to a few hours | Color the skin | 3 | 13 | 1 |  |
|  |  |  |  |  |  |  |  |  | Beautifies | 5 | 13,13 | 2 |  |
|  |  |  |  |  |  |  |  |  | Color nails | 3 | 13 | 1 |  |
|  |  |  |  |  |  |  |  | Create designs on the feet, leave on for a few minutes or hours, then rinse and apply body lotion or oil | Beautifies | 5 | 15 | 1 |  |
|  |  |  |  |  |  |  |  | Apply on hand | Beautifies | 5 | 13,33 | 2 |  |
|  |  |  |  |  |  |  |  | Apply on feet | Beautifies | 5 | 13,33 | 2 |  |
|  |  |  |  |  |  |  |  | Apply on the hands, creating patterns. Let it dry and set for a few minutes to hours, then rinse | Beautifies | 5 | 17,27 | 2 |  |
|  |  |  |  |  |  |  |  | Apply on the feet, creating patterns. Let it dry and set for a few minutes to hours, then rinse | Beautifies | 5 | 17,27 | 2 |  |
|  |  |  |  |  |  |  | Crush the *Lawsonia inermis* leaves and add the lemon juice from *Citrus* sp. Mix and let it sit for a few minutes. | Apply on hand | Beautifies | 5 | 5,26 | 2 |  |
|  |  |  |  |  |  |  |  | Apply on feet | Beautifies | 5 | 5,26 | 2 |  |
|  |  |  |  |  |  |  | Crush the *Lawsonia inermis* leaves, add the juice of *Citrus* sp. Possibility to add *Curcuma longa* according to taste | Apply using a matchstick on the skin of hands and feet to create designs. | Beautifies | 5 | 6 | 1 |  |
|  |  |  |  |  |  |  | Crush the *Lawsonia inermis* leaves and the rhizome of *Curcuma longa*, and mix it with oil, milk, and the fruit of *Cocos nucifera* | Apply on the body in circular massage motions | Cleanser | 5 | 8 | 1 |  |
|  |  |  |  |  |  |  |  |  | Softens | 2 | 8 | 1 |  |
|  |  |  |  |  |  |  |  |  | Hydrates | 2 | 8 | 1 |  |
|  |  |  |  |  |  |  |  |  | Nourishes | 2 | 8 | 1 |  |
|  |  |  |  |  |  |  |  |  | Beautifies | 5 | 8 | 1 |  |
|  |  |  |  |  |  |  | Crush the *Lawsonia inermis* leaves and add the lemon juice from *Citrus* sp. Then apply *Cocos nucifera* oil after rinsing | Apply on the soles of the feet and toenails. | Beautifies | 5 | 8 | 1 |  |
|  |  |  |  |  |  |  |  | Apply on the soles of the hand and toenails. | Beautifies | 5 | 8 | 1 |  |
|  |  |  |  |  |  |  | Crush the *Lawsonia inermis* leaves and the rhizome of *Curcuma longa*. Mix everything with petroleum | Body / skin | Cleanser | 5 | 9 | 1 |  |
|  |  |  |  |  |  |  |  |  | Beautifies | 5 | 9 | 1 |  |
|  |  |  |  |  |  |  | Crush the *Lawsonia inermis* leaves and the rhizome of *Curcuma longa*, and mix with coconut milk. | Body / skin | Cleanser | 5 | 16 | 1 |  |
|  |  |  |  |  |  |  |  |  | Brightens | 3 | 16 | 1 |  |
|  |  |  |  |  |  |  |  |  | Add shine | 3 | 16 | 1 |  |
|  |  |  |  |  |  |  |  | Face | Cleanser | 5 | 16 | 1 |  |
|  |  |  |  |  |  |  |  |  | Brightens | 3 | 16 | 1 |  |
|  |  |  |  |  |  |  |  |  | Add shine | 3 | 16 | 1 |  |
|  |  |  |  |  |  |  |  | Massage (face) | Add shine | 3 | 16 | 1 |  |
|  |  |  |  |  |  |  | Crush the *Lawsonia inermis* leaves and mix with coconut milk and *Sesamum indicum* oil | Massage (body) | Cleanser | 5 | 16 | 1 |  |
|  |  |  |  |  |  |  |  |  | Brightens | 3 | 16 | 1 |  |
|  |  |  |  |  |  |  |  |  | Add shine | 3 | 16 | 1 |  |
|  |  |  |  |  |  |  |  | Massage (face) | Cleanser | 5 | 16 | 1 |  |
|  |  |  |  |  |  |  |  |  | Brightens | 3 | 16 | 1 |  |
|  |  |  |  |  |  |  |  |  | Add shine | 3 | 16 | 1 |  |
|  |  |  |  |  |  |  | Crush the *Lawsonia inermis* leaves and the rhizome of *Curcuma longa*, mix with coconut milk, and *Sesamum indicum* oil | ND | Cleanser | 5 | 16 | 1 |  |
|  |  |  |  |  |  |  |  |  | Brightens | 3 | 16 | 1 |  |
|  |  |  |  |  |  |  |  |  | Add shine | 3 | 16 | 1 |  |
|  |  |  |  |  |  |  |  |  | Cleanser | 5 | 16 | 1 |  |
|  |  |  |  |  |  |  |  |  | Brightens | 3 | 16 | 1 |  |
|  |  |  |  |  |  |  |  |  | Add shine | 3 | 16 | 1 |  |
|  |  |  |  |  |  |  | Crush the *Lawsonia inermis* leaves and the rhizome of *Curcuma longa*, and mix everything with the juice of *Citrus* sp. | Apply on the hands, creating designs. Leave for a few hours. Then rinse | Beautifies | 5 | 16 | 1 |  |
|  |  |  |  |  |  |  |  | Apply on the feet, creating designs. Leave for a few hours. Then rinse | Beautifies | 5 | 16 | 1 |  |
|  |  |  |  |  |  |  | Crush the *Lawsonia inermis* leaves, extract the milk from *Cocos nucifera*, crush the *Curcuma longa*. Mix everything | Massage (body) | Beautifies | 5 | 17 | 1 |  |
|  |  |  |  |  |  |  |  |  | Tanned complexion | 3 | 17 | 1 |  |
|  |  |  |  |  |  |  |  |  | Brightens | 3 | 17 | 1 |  |
|  |  |  |  |  |  |  |  | Massage (face) | Beautifies | 5 | 17 | 1 |  |
|  |  |  |  |  |  |  |  |  | Tanned complexion | 3 | 17 | 1 |  |
|  |  |  |  |  |  |  |  |  | Brightens | 3 | 17 | 1 |  |
|  |  |  |  |  |  |  | Crush the *Lawsonia inermis* leaves and S*esamum indicum* seeds together | Face | Cleanser | 5 | 18 | 1 |  |
|  |  |  |  |  |  |  |  | Body / skin | Cleanser | 5 | 18 | 1 |  |
|  |  |  |  |  |  |  |  | Massage (body) | Cleanser | 5 | 20 | 1 |  |
|  |  |  |  |  |  |  |  |  | Lightens | 3 | 20 | 1 |  |
|  |  |  |  |  |  |  |  |  | Brightens | 3 | 20 | 1 |  |
|  |  |  |  |  |  |  |  | Massage (face) | Cleanser | 5 | 20 | 1 |  |
|  |  |  |  |  |  |  |  |  | Lightens | 3 | 20 | 1 |  |
|  |  |  |  |  |  |  |  |  | Brightens | 3 | 20 | 1 |  |
|  |  |  |  |  |  |  |  | Exfoliation | Cleanser | 5 | 34 | 1 |  |
|  |  |  |  |  |  |  |  |  | Lightens | 3 | 34 | 1 |  |
|  |  |  |  |  |  |  | Crush the *Lawsonia inermis* leaves, the roots of *Chrysopogon zizanoides*, and the rhizome of *Curcuma longa*. Mix everything with the oil and milk of *Cocos nucifera* | Exfoliation | Cleanser | 5 | 23 | 1 |  |
|  |  |  |  |  |  |  |  |  | Beautifies | 5 | 23 | 1 |  |
|  |  |  |  |  |  |  |  |  | Hydrates | 2 | 23 | 1 |  |
|  |  |  |  |  |  |  |  |  | Softens | 2 | 23 | 1 |  |
|  |  |  |  |  |  |  | Crush the *Lawsonia inermis* leaves and add petroleum | Apply on the feet and heels | Heal the cracks | 4 | 24,24 | 2 |  |
|  |  |  |  |  |  |  | Crush the *Lawsonia inermis* leaves, crush the rhizome, or add the powder of *Curcuma longa* and *Sesamum indicum* oil | Apply by massaging onto the body, performing gentle massages, and rinse with warm water | Cleanser | 5 | 26 | 1 |  |
|  |  |  |  |  |  |  |  |  | Beautifies | 5 | 26 | 1 |  |
|  |  |  |  |  |  |  |  |  | Nourishes | 2 | 26 | 1 |  |
|  |  |  |  |  |  |  |  |  | Brightens | 3 | 26 | 1 |  |
|  |  |  |  |  |  |  |  | Apply by massaging onto the face, performing gentle massages, and rinse with warm water | Cleanser | 5 | 26 | 1 |  |
|  |  |  |  |  |  |  |  |  | Beautifies | 5 | 26 | 1 |  |
|  |  |  |  |  |  |  |  |  | Nourishes | 2 | 26 | 1 |  |
|  |  |  |  |  |  |  |  |  | Brightens | 3 | 26 | 1 |  |
|  |  |  |  |  |  |  | Crush the *Lawsonia inermis* leaves and the rhizome of *Curcuma longa*, mix with *Sesamum indicum* oil | Exfoliation | Cleanser | 5 | 32 | 1 |  |
|  |  |  |  |  |  |  |  |  | Brightens | 3 | 32 | 1 |  |
|  |  |  |  |  |  |  |  |  | Anti-aging | 2 | 32 | 1 |  |
|  |  |  |  |  |  |  |  |  | Softens | 2 | 32 | 1 |  |
|  |  |  |  |  |  |  |  |  | Tightens | 2 | 32 | 1 |  |
|  |  |  |  |  |  |  |  |  | Light complexion | 3 | 32 | 1 |  |
|  |  |  |  |  |  |  | Crush the rhizome of *Curcuma longa*, the leaves of *Lawsonia inermis*, and the roots of *Chrysopogon zizanoides*. Mix everything with coconut milk and oil. Allow to marinate for a few minutes or hours | Exfoliation | Cleanser | 5 | 4 | 1 |  |
|  |  |  |  |  |  |  |  |  | Beautifies | 5 | 4 | 1 |  |
|  |  |  |  |  |  |  |  |  | Hydrates | 2 | 4 | 1 |  |
|  |  |  |  |  |  |  |  |  | Softens | 2 | 4 | 1 |  |
| *Lythraceae* | *Sonneratia alba* Sm | Manglier fleur | ND | M'honko ndziwi | Honko bé |  |  |  |  |  |  |  | ODAM049 |
|  |  |  |  |  |  | Leaf | ND | Apply the juice of the leaves on body / skin | Dermatoses | 1 | 3 | 1 |  |
| *Malvaceae* | *Ceiba pentandra* (L.) Gaertn. | Kapokier | Silk-cotton tree, Kapok tree | M'pembafouma | Pembafuma |  |  |  |  |  |  |  | *ODAM003* |
|  |  |  |  |  |  | Leaf | Crush the leaves in water | Hair | Cleanser | 4 | 4 | 1 |  |
|  |  |  |  |  |  |  |  |  | Nourishes | 4 | 4 | 1 |  |
|  |  |  |  |  |  |  |  | Apply on and in the hair. Comb and brush the hair, then rinse. | Softens | 4 | 9 | 1 |  |
|  |  |  |  |  |  |  |  |  | Elongates the curl | 4 | 9 | 1 |  |
|  |  |  |  |  |  |  |  |  | Minimizes frizz | 4 | 9 | 1 |  |
|  |  |  |  |  |  |  |  |  | Nourishes | 4 | 9 | 1 |  |
|  |  |  |  |  |  |  |  |  | Stimulates hair growth | 4 | 9 | 1 |  |
|  |  |  |  |  |  |  |  |  | Cleanser | 4 | 13,13 | 2 |  |
|  |  |  |  |  |  |  |  |  | Detangles | 4 | 13,13 | 2 |  |
|  |  |  |  |  |  |  |  | Apply on the hair and rub like a shampoo |  | 4 | 13,19 | 2 |  |
|  |  |  |  |  |  |  | Mix the leaves with coconut oil and let it soak | Hair and style | Stimulates hair growth | 4 | 4 | 1 |  |
|  |  |  |  |  |  |  |  |  | Nourishes | 4 | 4 | 1 |  |
|  |  |  |  |  |  |  |  |  | Beautifies | 5 | 4 | 1 |  |
|  |  |  |  |  |  |  | Crush the leaves of "shifundja koli" et *Ceiba pentrada* | Shampoo | Stimulates hair growth | 4 | 34 | 1 |  |
|  |  |  |  |  |  |  |  |  | Softens | 4 | 34 | 1 |  |
| *Malvaceae* | *Cola nitida* (Vent.) Schott & Endl. | Colatier | Kola | Kola | Kola |  |  |  |  |  |  |  | ODAM53 |
|  |  |  |  |  |  | Fruit | Not preparation recquired | The seeds are consumed | Stimulates | 5 | 30 | 1 |  |
|  |  |  |  |  |  |  |  |  | Tones | 5 | 30 | 1 |  |
|  |  |  |  |  |  |  |  |  | Energyzing | 5 | 30 | 1 |  |
| *Malvaceae* | *Hibiscus schizopetalus* (Dyer) Hook.f. | Lanterne japonaise | ND | ND | Sary kafe maféki |  |  |  |  |  |  |  | ODAM051 |
|  |  |  |  |  |  | Flower | Harvest the flower with the end containing the yellow powder | Apply the end with the yellow powder on the face, by tapping. Allows for a pattern to be created from the powder | Make up | 5 | 19 | 1 |  |
| *Malvaceae* | *Sida rhombifolia* L. | Faux thé | Queensland hemp | Shifunga koli | ND |  |  |  |  |  |  |  | ODAM050 |
|  |  |  |  |  |  | Leaf | Crush the leaves and put them in water, then mix | Face | Cleanser | 4 | 17 | 1 |  |
|  |  |  |  |  |  |  |  | Hair | Shampoo | 4 | 17 | 1 |  |
|  |  |  |  |  |  |  | Create a pulp from the entire plant | Body / skin | Antiseptic | 1 | 17 | 1 |  |
| *Moraceae* | *Ficus sycomorus* L. | Le figuier sycomore | ND | Muhu mambe | Adabu |  |  |  |  |  |  |  | ODAM052 |
|  |  |  |  |  |  | Latex | Extract latex from the tree | Body / skin | Anti pimples | 1 | 34 | 1 |  |
| *Musaceae* | *Musa* sp. | Bananier | Banana tree | Tchindri | Voudi ni outsi |  |  |  |  |  |  |  | ODAM055 |
|  |  |  |  |  |  | Leaf | Crush the leaves in water | Wash | Tightens (baby skin) | 5 | 1 | 1 |  |
| *Myristicaceae* | *Myristica fragrans* Houtt. | Muscadier | Nutmeg | Kougoumanga | Kougoumanga |  |  |  |  |  |  |  | NC |
|  |  |  |  |  |  | Seed | The seed on the coral stone with a little water until a paste is obtained | In case of swelling, apply and let it sit for a few minutes or even a few hours | Swelling | 1 | 29 | 1 |  |
| *Myrtaceae* | *Syzygium aromaticum* (L.) Merr. & L.M.Perry | Giroflier | Clove tree | Karafu | Karafo |  |  |  |  |  |  |  | NC |
|  |  |  |  |  |  | clove | Crush 1 or 2 cloves of *Syzygium aromaticum* with the msidzano, adding a bit of water on the coral stone until a smooth paste is obtained | Face | Cleanser | 5 | 9 | 1 |  |
|  |  |  |  |  |  |  |  |  | Beautifies | 5 | 9 | 1 |  |
|  |  |  |  |  |  |  |  |  | Fragrance | 5 | 9 | 1 |  |
|  |  |  |  |  |  |  | Decoction | Mouthwash | Dental care | 5 | 1,13,26 | 3 |  |
|  |  |  |  |  |  |  | Macerate the cloves of *Syzygium aromaticum* in coconut oil for a few days or weeks | Hair | Stimulates hair growth | 4 | 16 | 1 |  |
|  |  |  |  |  |  |  |  |  | Softens | 4 | 16 | 1 |  |
| *Nyctaginaceae* | *Bougainvillea spectabilis* Willd. | Bougainvillées | Great bougainvillea | ND | Telo myova |  |  |  |  |  |  |  | *ODAM020* |
|  |  |  |  |  |  | Flower | Stringing the flowers of *Jasminum nummulariaefolium, Plumeria alba,* and *Bougainvillea spectabilis* onto a necklace | Neck | Beautifies | 5 | 5 | 1 |  |
|  |  |  |  |  |  |  |  |  | Fragrance | 5 | 5 | 1 |  |
|  |  |  |  |  |  |  | Craft a necklace with a mixture of flowers from *Jasminum nummulariaefolium, Plumeria alba, Cananga odorata, Bougainvillea spectabilis*, and leaves of *Pogostemon cablin*, as well as leaves and inflorescences of *Ocimum* spp. | Neck | Beautifies | 5 | 11 | 1 |  |
|  |  |  |  |  |  |  |  |  | Fragrance | 5 | 11 | 1 |  |
|  |  |  |  |  |  |  | Pin a mixture of flowers from *Jasminum nummulariaefolium* and *Bougainvillea spectabilis* with safety pins | Hair | Beautifies | 5 | 11 | 1 |  |
|  |  |  |  |  |  |  |  |  | Fragrance | 5 | 11 | 1 |  |
|  |  |  |  |  |  |  |  | Clothing | Beautifies | 5 | 11 | 1 |  |
|  |  |  |  |  |  |  |  |  | Fragrance | 5 | 11 | 1 |  |
|  |  |  |  |  |  |  | Not preparation recquired | Arrange a mixture of flowers from Jasminum nummulariaefolium and Bougainvillea spectabilis on the bed | Fragrance | 5 | 11 | 1 |  |
| *Oleaceae* | *Jasminum nummulariifolium* Baker | Jasmin | ND | Anfu | Anfu |  |  |  |  |  |  |  | ODAM056 |
|  |  |  |  |  |  | Flower | Not preparation recquired | Place on the bed | Fragrance | 5 | 4,4,5,5,5,5,5,5,7,13,23,32,33 | 13 |  |
|  |  |  |  |  |  |  |  |  | Beautifies | 5 | 10,23 | 2 |  |
|  |  |  |  |  |  |  |  | The flowers are placed on hairpins in the hair | Fragrance | 5 | 13,13 | 2 |  |
|  |  |  |  |  |  |  |  |  | Beautifies | 5 | 13,13 | 2 |  |
|  |  |  |  |  |  |  |  | Arrange on the bed a mixture of leaves and inflorescences of *Ocimum* spp., the flowers of *Pandanus maximus*, *Jasminum nummulariaefolium*, and *Cananga odorata* | Fragrance | 5 | 23 | 1 |  |
|  |  |  |  |  |  |  |  |  | Beautifies | 5 | 23 | 1 |  |
|  |  |  |  |  |  |  |  | Arrange a mixture of flowers from *Cananga odorata* and *Jasminum nummulariaefolium* on the bed. | Fragrance | 5 | 11 | 1 |  |
|  |  |  |  |  |  |  |  | Arrange on the bed a mixture of flowers from *Jasminum nummulariaefolium*, *Pandanus maximus*, with leaves and inflorescences of *Ocimum* spp | Fragrance | 5 | 12 | 1 |  |
|  |  |  |  |  |  |  |  | Arrange on the bed a mixture of flowers from *Jasminum nummulariaefolium* and *Bougainvillea spectabilis* | Fragrance | 5 | 11 | 1 |  |
|  |  |  |  |  |  |  |  | Arrange on the bed a mixture of flowers from *Jasminum nummulariaefolium* and leaves and inflorescences of *Ocimum* spp. | Fragrance | 5 | 11 | 1 |  |
|  |  |  |  |  |  |  |  | Arrange on the bed a mixture of flowers from *Jasminum nummulariaefolium* and leaves of *Pogostemon cablin* | Fragrance | 5 | 11 | 1 |  |
|  |  |  |  |  |  |  |  | Arrange on the bed a mixture of flowers from *Jasminum nummulariaefolium* and *Vachellia farnesiana* | Fragrance | 5 | 11 | 1 |  |
|  |  |  |  |  |  |  |  | Arrange on the bed a mixture of flowers from *Rosa alba* and *Jasminum nummulariaefolium* | Fragrance | 5 | 11 | 1 |  |
|  |  |  |  |  |  |  |  | Arrange on the bed a mixture of flowers from *Rosa alba* and *Jasminum nummulariaefolium*, along with *Vachellia farnesiana*, and include leaves and inflorescences of *Ocimum* spp. | Fragrance | 5 | 11 | 1 |  |
|  |  |  |  |  |  |  |  | Arrange on the bed a mixture of flowers from *Rosa alba* and *Jasminum nummulariaefolium*, along with *Vachellia farnesiana* | Fragrance | 5 | 11 | 1 |  |
|  |  |  |  |  |  |  | Crush the rhizome of *Curcuma longa* and mix it with the fragrant oil composed of *Cocos nucifera* oil, *Vetiveria zizanioides* root, flowers of *Pandanus maximus, Jasminum nummulariaefolium*, and fragrant plants from "Salika" | Massage (Body) | Cleanser.Note: Turmeric may impart color | 5 | 9 | 1 |  |
|  |  |  |  |  |  |  |  |  | Beautifies. Note: Turmeric may impart color. | 5 | 9 | 1 |  |
|  |  |  |  |  |  |  | Crush the leaves and inflorescences of *Ocimum* spp., the rhizome of *Curcuma longa*, the roots of *Chrysopogon zizanioides*, the flowers of *Rosa alba*, *Pandanus maximus, Jasminum nummulariaefolium*, and *Vachellia farnesiana*. Mix everything with the milk and grated fruit of *Cocos nucifera*. Let it soak for a few minutes or even hours. | Exfoliation | Skin care | 2 | 5 | 1 |  |
|  |  |  |  |  |  |  |  |  | Cleanser | 5 | 5 | 1 |  |
|  |  |  |  |  |  |  |  |  | Softens | 2 | 5 | 1 |  |
|  |  |  |  |  |  |  |  |  | Nourishes | 2 | 5 | 1 |  |
|  |  |  |  |  |  |  |  |  | Beautifies | 5 | 5 | 1 |  |
|  |  |  |  |  |  |  |  |  | Hydrates | 2 | 5 | 1 |  |
|  |  |  |  |  |  |  | Create a necklace with a mixture of flowers from *Jasminum nummulariaefolium, Plumeria alba, Cananga odorata, Bougainvillea spectabilis*, as well as leaves of *Pogostemon cablin* and leaves and inflorescences of *Ocimum* spp. | Neck | Fragrance | 5 | 11 | 1 |  |
|  |  |  |  |  |  |  |  |  | Beautifies | 5 | 11 | 1 |  |
|  |  |  |  |  |  |  | Create a mixture with the flowers of J*asminum nummulariaefolium* and *Cananga odorata*, and place them on hairpins | Hair | Beautifies | 5 | 16 | 1 |  |
|  |  |  |  |  |  |  | Create a mixture with the flowers of *Jasminum nummulariaefolium* and *Cananga odorata*, and put them on a necklace | Neck | Beautifies | 5 | 16 | 1 |  |
|  |  |  |  |  |  |  | Create a mixture with the flowers of *Jasminum nummulariaefolium, Rosa alba*, and *Vachellia farnesiana,* and put them on hairpins | Hair | Beautifies | 5 | 16 | 1 |  |
|  |  |  |  |  |  |  | Create a mixture with the flowers of *Jasminum nummulariaefolium, Rosa alba*, and *Vachellia farnesiana,* and put them on a necklace (toka) | Neck | Beautifies | 5 | 16 | 1 |  |
|  |  |  |  |  |  |  | Create a mixture with the flowers of *Jasminum nummulariaefolium* and the leaves and inflorescences of *Ocimum* spp., and put them on safety pins | Hair | Beautifies | 5 | 16 | 1 |  |
|  |  |  |  |  |  |  | Create a mixture with the flowers of *Jasminum nummulariaefolium* and the leaves and inflorescences of *Ocimum* spp., and put them on a necklace | Neck | Beautifies | 5 | 16 | 1 |  |
|  |  |  |  |  |  |  | The flowers of *Cananga odorata* and *Jasminum nummulariaefolium* are placed on safety pins | Hair | Beautifies | 5 | 14 | 1 |  |
|  |  |  |  |  |  |  |  |  | Fragrance | 5 | 14 | 1 |  |
|  |  |  |  |  |  |  | The flowers of *Cananga odorata* and *Jasminum nummulariaefolium* are used in the making of a necklace | Neck | Beautifies | 5 | 14 | 1 |  |
|  |  |  |  |  |  |  |  |  | Fragrance | 5 | 14 | 1 |  |
|  |  |  |  |  |  |  | Mix together the following plants: roots of *Chrysopogon zizanoides*, leaves and inflorescences of *Ocimum* spp., leaves of *Ayapana triplinervis*, flowers of *Jasminum nummulariaefolium*, *Pandanus maximus, Cananga odorata*; obtained from safety pins. Introduce them into coconut oil and let them soak in the oil. | Body / skin | Beautifies | 5 | 5 | 1 |  |
|  |  |  |  |  |  |  |  |  | Fragrance | 5 | 5 | 1 |  |
|  |  |  |  |  |  |  |  |  | Softens | 5 | 5 | 1 |  |
|  |  |  |  |  |  |  |  |  | Post-depilatory care | 5 | 5 | 1 |  |
|  |  |  |  |  |  |  | Mix the leaves and inflorescences of *Ocimum* sp. with the flowers of *Pandanus maximus, Rosa alba, Jasminum nummulariaefolium, Cananga odorata*, and roots of *Chrysopogon zizanoïdes*. Incorporate everything into coconut oil and let it marinate | Body / skin | Beautifies skin | 5 | 4,4 | 2 |  |
|  |  |  |  |  |  |  |  |  | Fragrance | 5 | 4,4 | 2 |  |
|  |  |  |  |  |  |  | Mix the dried flowers of *Pandanus maximus, Jasminum nummulariaefolium, Cananga odorata*, the dried leaves of *Pogostemon cablin, Ayapana triplinervis*, the dried leaves and inflorescences of *Ocimum* spp., and the root of *Chrysopogon zizanoides* "zoukouba" | ND | Fragrance | 5 | 11 | 1 |  |
|  |  |  |  |  |  |  | Put the flowers on safety pins "kilabou" | Hair | Beautifies | 5 | 17,33,21,5,9,10 | 6 |  |
|  |  |  |  |  |  |  |  |  | Fragrance | 5 | 5,33,9,10,10 | 5 |  |
|  |  |  |  |  |  |  |  | Clothing | Beautifies | 5 | 33 | 1 |  |
|  |  |  |  |  |  |  |  |  | Fragrance | 5 | 33 | 1 |  |
|  |  |  |  |  |  |  | Dry and sift the leaves of *Phymatosorus scolopendria* and the flowers of *Jasminum nummulariaefolium*. Put them in coconut oil and let them soak | Massage (Body) | Hydrates | 2 | 19 | 1 |  |
|  |  |  |  |  |  |  |  |  | Cleanser | 5 | 19 | 1 |  |
|  |  |  |  |  |  |  |  |  | Beautifies | 5 | 19 | 1 |  |
|  |  |  |  |  |  |  | Place the dried leaves and inflorescences of *Ocimum* spp., the dried roots of *Chrysopogon zizanoides*, the dried flowers of *Pandanus maximus*, and *Jasminum nummulariaefolium* in coconut milk and add grated fruit. | Massage (Body) | Skin care | 2 | 23 | 1 |  |
|  |  |  |  |  |  |  |  |  | Cleanser | 5 | 23 | 1 |  |
|  |  |  |  |  |  |  |  |  | Softens | 2 | 23 | 1 |  |
|  |  |  |  |  |  |  |  |  | Nourishes | 2 | 23 | 1 |  |
|  |  |  |  |  |  |  |  |  | Beautifies | 5 | 23 | 1 |  |
|  |  |  |  |  |  |  |  |  | Hydrates | 2 | 23 | 1 |  |
|  |  |  |  |  |  |  |  |  | Fragrance | 5 | 23 | 1 |  |
|  |  |  |  |  |  |  | Puts flower on necklace | Neck | Beautifies | 5 | 33,17 | 2 |  |
|  |  |  |  |  |  |  |  |  | Fragrance | 5 | 33,10, | 2 |  |
|  |  |  |  |  |  |  | String the flowers of *Jasminum nummulariaefolium* and *Plumeria alba* onto a necklace | Neck | Fragrance | 5 | 5 | 1 |  |
|  |  |  |  |  |  |  |  | Neck | Beautifies | 5 | 5 | 1 |  |
|  |  |  |  |  |  |  |  | Neck | Fragrance | 5 | 5 | 1 |  |
|  |  |  |  |  |  |  |  | Neck | Beautifies | 5 | 5 | 1 |  |
|  |  |  |  |  |  |  | Infuse the dried plants, including the flowers of *Jasminum nummulariaefolium, Pandanus maximus, Plumeria alba, Cananga odorata, Rosa alba,* the roots of *Chrysopogon zizanoides*, the leaves of *Pogostemon cablin, Ayapana triplinervis*, and the leaves and inflorescence of *Ocimum* spp., in coconut oil. | Body / skin | Fragrance | 5 | 10 | 1 |  |
|  |  |  |  |  |  |  | String the flowers of *Jasminum nummulariaefolium*, *Plumeria alba*, and *Bougainvillea spectabilis* onto a necklace | Neck | Beautifies | 5 | 5 | 1 |  |
|  |  |  |  |  |  |  |  |  | Fragrance | 5 | 5 | 1 |  |
|  |  |  |  |  |  |  | String a mixture of flowers from *Cananga odorata* and *Jasminum nummulariaefolium* onto a necklace. | Neck | Beautifies | 5 | 26 | 1 |  |
|  |  |  |  |  |  |  |  | Hair | Beautifies | 5 | 26 | 1 |  |
|  |  |  |  |  |  |  |  | Clothing | Beautifies | 5 | 26 | 1 |  |
|  |  |  |  |  |  |  | String a mixture of leaves and inflorescences from *Ocimum* spp. and *Jasminum nummulariaefolium* onto a necklace | Neck | Beautifies | 5 | 32 | 1 |  |
|  |  |  |  |  |  |  |  |  | Fragrance | 5 | 32 | 1 |  |
|  |  |  |  |  |  |  | String a mixture of flowers from *Jasminum nummulariaefolium* and *Vachellia farnesiana* onto a necklace | Neck | Beautifies | 5 | 26 | 1 |  |
|  |  |  |  |  |  |  | Place on safety pins a mixture of flowers from *Jasminum nummulariaefolium, Vachellia farnesiana*, and leaves and inflorescences of *Ocimum* spp. | Hair | Beautifies | 5 | 11,27,5 | 3 |  |
|  |  |  |  |  |  |  |  |  | Fragrance | 5 | 11,5 | 2 |  |
|  |  |  |  |  |  |  |  | Clothing | Beautifies | 5 | 11,27,5 | 3 |  |
|  |  |  |  |  |  |  |  |  | Fragrance | 5 | 11,5 | 2 |  |
|  |  |  |  |  |  |  | Place on nackelace a mixture of flowers from *Jasminum nummulariaefolium, Vachellia farnesiana*, and leaves and inflorescences of *Ocimum* spp. | Neck | Beautifies | 5 | 27 | 1 |  |
|  |  |  |  |  |  |  | Place on safety pins a mixture of flowers from *Jasminum nummulariaefolium, Vachellia farnesiana* | Hair | Beautifies | 5 | 26 | 1 |  |
|  |  |  |  |  |  |  |  | Clothing | Beautifies | 5 | 26 | 1 |  |
|  |  |  |  |  |  |  | Put a mixture of flowers from *Cananga odorata* and *Jasminum nummulariaefolium* on safety pins | Hair | Beautifies | 5 | 9,11 | 2 |  |
|  |  |  |  |  |  |  |  |  | Fragrance | 5 | 9,11 | 2 |  |
|  |  |  |  |  |  |  |  | Clothing | Beautifies | 5 | 11 | 1 |  |
|  |  |  |  |  |  |  |  |  | Fragrance | 5 | 11 | 1 |  |
|  |  |  |  |  |  |  | Put a mixture of flowers from *Jasminum nummulariaefolium*, *Pandanus maximus*, along with leaves and inflorescences of *Ocimum* spp. on safety pins | Hair | Beautifies | 5 | 12 | 1 |  |
|  |  |  |  |  |  |  |  |  | Fragrance | 5 | 12 | 1 |  |
|  |  |  |  |  |  |  |  | Clothing | Beautifies | 5 | 12 | 1 |  |
|  |  |  |  |  |  |  |  |  | Fragrance | 5 | 12 | 1 |  |
|  |  |  |  |  |  |  | Put a mixture of flowers from *Jasminum nummulariaefolium* and *Vachellia farnesiana* on safety pins | Hair | Beautifies | 5 | 11,21,9 | 3 |  |
|  |  |  |  |  |  |  |  |  | Fragrance | 5 | 11,9 | 2 |  |
|  |  |  |  |  |  |  |  | Clothing | Beautifies | 5 | 11 | 1 |  |
|  |  |  |  |  |  |  |  |  | Fragrance | 5 | 11 | 1 |  |
|  |  |  |  |  |  |  | Put a mixture of flowers from *Jasminum nummulariaefolium* and *Vachellia farnesiana* on nackelace | Clothing | Fragrance | 5 | 21 | 1 |  |
|  |  |  |  |  |  |  | Put a mixture of leaves and inflorescences from *Ocimum* spp. and *Jasminum nummulariaefolium* on safety pins | Hair | Beautifies | 5 | 32,11,5 | 3 |  |
|  |  |  |  |  |  |  |  |  | Fragrance | 5 | 32,11,5 | 3 |  |
|  |  |  |  |  |  |  |  | Clothing | Beautifies | 5 | 32,11,5 | 3 |  |
|  |  |  |  |  |  |  |  |  | Fragrance | 5 | 32,11,5 | 3 |  |
|  |  |  |  |  |  |  | Put a mixture of flowers from *Rosa alba* and *Jasminum nummulariaefolium* on safety pins | Hair | Beautifies | 5 | 11 | 1 |  |
|  |  |  |  |  |  |  |  |  | Fragrance | 5 | 11 | 1 |  |
|  |  |  |  |  |  |  |  | Clothing | Beautifies | 5 | 11 | 1 |  |
|  |  |  |  |  |  |  |  |  | Fragrance | 5 | 11 | 1 |  |
|  |  |  |  |  |  |  | Put a mixture of flowers from *Rosa alba* , *Jasminum nummulariaefolium* on and *Vachellia farnesiana* safety pins | Hair | Beautifies | 5 | 11 | 1 |  |
|  |  |  |  |  |  |  |  |  | Fragrance | 5 | 11 | 1 |  |
|  |  |  |  |  |  |  |  | Clothing | Beautifies | 5 | 11 | 1 |  |
|  |  |  |  |  |  |  |  |  | Fragrance | 5 | 11 | 1 |  |
|  |  |  |  |  |  |  | Put a mixture of flowers from *Jasminum nummulariaefolium* and leaves of *Pogostemon cablin* on safety pins | Hair | Beautifies | 5 | 11 | 1 |  |
|  |  |  |  |  |  |  |  |  | Fragrance | 5 | 11 | 1 |  |
|  |  |  |  |  |  |  |  | Clothing | Beautifies | 5 | 11 | 1 |  |
|  |  |  |  |  |  |  |  |  | Fragrance | 5 | 11 | 1 |  |
|  |  |  |  |  |  |  | Put a mixture of flowers from *Jasminum nummulariaefolium* and *Bougainvillea spectabilis* on safety pins | Hair | Beautifies | 5 | 11 | 1 |  |
|  |  |  |  |  |  |  |  |  | Fragrance | 5 | 11 | 1 |  |
|  |  |  |  |  |  |  |  | Clothing | Beautifies | 5 | 11 | 1 |  |
|  |  |  |  |  |  |  |  |  | Fragrance | 5 | 11 | 1 |  |
|  |  |  |  |  |  |  | Place the leaves and inflorescences of *Ocimum* spp. on the "kilabou", mixing them with the flowers of *Jasminum nummulariaefolium* and *Rosa alba* | Hair | Beautifies | 5 | 5 | 1 |  |
|  |  |  |  |  |  |  |  |  | Fragrance | 5 | 5 | 1 |  |
|  |  |  |  |  |  |  |  | Clothing | Beautifies | 5 | 5 | 1 |  |
|  |  |  |  |  |  |  |  |  | Fragrance | 5 | 5 | 1 |  |
|  |  |  |  |  |  |  | Put the flowers of Jasminum nummulariaefolium and Plumeria alba on a toka | Neck | Beautifies | 5 | 5,5 | 2 |  |
|  |  |  |  |  |  |  |  |  | Fragrance | 5 | 5,5 | 2 |  |
|  |  |  |  |  |  |  |  | Hair | Beautifies | 5 | 5,5 | 2 |  |
|  |  |  |  |  |  |  |  |  | Fragrance | 5 | 5,5 | 2 |  |
|  |  |  |  |  |  |  | Put a mixture of flowers from *Jasminum nummulariaefolium, Vachellia farnesiana, Rosa alba*, and leaves and inflorescences of *Ocimum* spp. on safety pins | Hair | Beautifies | 5 | 10 | 1 |  |
|  |  |  |  |  |  |  |  |  | Fragrance | 5 | 10 | 1 |  |
|  |  |  |  |  |  |  | Place a mixture of leaves and inflorescences of *Ocimum* spp., flowers of *Pandanus maximus, Jasminum nummulariaefolium*, and *Cananga odorata* on a kilabou | Hair | Beautifies | 5 | 23 | 1 |  |
|  |  |  |  |  |  |  |  |  | Fragrance | 5 | 23 | 1 |  |
|  |  |  |  |  |  |  | Pin the flowers, based on availability and preference, of *Jasminum nummulariaefolium, Pandanus maximus, Vachellia farnesiana, Cananga odorata, Rosa alba*, "Koukoumba", *Cestrum nocturnum* using a safety pin and stack them on it. | Hair | Beautifies | 5 | 7 | 1 |  |
|  |  |  |  |  |  |  |  |  | Fragrance | 5 | 7 | 1 |  |
|  |  |  |  |  |  |  | Take the mixture of plants obtained from marriages or create your own blend based on plant availability (Roots of *Chrysopogon zizanoides*, Leaves of *Ayapana triplinervis*, Leaves and inflorescences of *Ocimum* spp., Flowers of *Jasminum nummulariaefolium, Vachellia farnesiana, Rosa alba*, "Koukoumba", P*lumeria alba, Cananga odorata, Pandanus maximus*). Once dried, crush and sort them by size. Prepare the coconut oil. To do this, grate the coconuts, extract the milk, let it sit overnight, remove the cream on top, and then cook it in a large pot over a wood fire. Once the oil is ready, add the flower mixture "zoukouba" when the temperature has cooled slightly, and add the pompeia when the oil is no longer very hot. Let it all macerate. The longer the maceration, the more intense the fragrance will be | Body / skin | Nourishes | 2 | 6 | 1 |  |
|  |  |  |  |  |  |  |  |  | Add shine | 3 | 6 | 1 |  |
|  |  |  |  |  |  |  |  |  | Softens | 2 | 6 | 1 |  |
|  |  |  |  |  |  |  |  |  | Fragrans | 5 | 6 | 1 |  |
|  |  |  |  |  |  |  |  |  | Lightens | 3 | 6 | 1 |  |
|  |  |  |  |  |  |  |  |  | Hydrates | 2 | 6 | 1 |  |
|  |  |  |  |  |  |  | Pierce the flowers using a needle and fill a thread with rice grains. Tie it all together | Neck | Beautifies | 5 | 2,3 | 2 |  |
|  |  |  |  |  |  |  | Pin the flowers using a safety pin and form a pile on it | Hair | Beautifies | 5 | 2,3,4 | 3 |  |
|  |  |  |  |  |  |  |  |  | Fragrance | 5 | 4 | 1 |  |
|  |  |  |  |  |  |  |  | Clothing | Beautifies | 5 | 2 | 1 |  |
|  |  |  |  |  |  |  | Pin the flowers using a safety pin and form a pile on it, creating mixtures to taste with jasmine flowers, Farnese steel, grand vacoa, or leaves and inflorescences of basil | Hair | Fragrance | 5 | 4 | 1 |  |
|  |  |  |  |  |  |  |  |  | Beautifies | 5 | 4 | 1 |  |
| *Orchidaceae* | *Vanilla* sp. | Vanille | Vanilla | Lavany | Lavany |  |  |  |  |  |  |  |  |
|  |  |  |  |  |  | Pod | Put the *Vanilla* sp pods in coconut oil and let them macerate for several weeks | Body / skin | Fragrance | 5 | 7 | 1 |  |
|  |  |  |  |  |  |  |  |  | Hydrates | 2 | 7 | 1 |  |
|  |  |  |  |  |  |  |  |  | Protect | 2 | 7 | 1 |  |
|  |  |  |  |  |  |  |  | Face | Fragrance | 5 | 7 | 1 |  |
|  |  |  |  |  |  |  |  |  | Hydrates | 2 | 7 | 1 |  |
|  |  |  |  |  |  |  |  |  | Protect | 2 | 7 | 1 |  |
|  |  |  |  |  |  |  | Place the vanilla pod in coconut oil for 6 months | Body / skin | Fragrance | 5 | 18 | 1 |  |
|  |  |  |  |  |  |  |  |  | Well-being | 5 | 18 | 1 |  |
|  |  |  |  |  |  |  |  | Face | Fragrance | 5 | 18 | 1 |  |
|  |  |  |  |  |  |  |  |  | Well-being | 5 | 18 | 1 |  |
| *Oxalidaceae* | *Averrhoa bilimbi* L. | Bilimbi | Bilimbi | Uhaju | Madiro antanana |  |  |  |  |  |  |  | ODAM057 |
|  |  |  |  |  |  | Fruit | Not preparation recquired | Crush the fruit on fingers stained by the fruits of *Musa sp*. or *Curcuma longa* or others | Anti-pimples | 1 | 28 | 1 |  |
| *Pandanaceae* | *Pandanus maximus* Martelli | Grand vacoa | ND | M'lua n'dzishe | Droa |  |  |  |  |  |  |  | ODAM058 |
|  |  |  |  |  |  | Flower | ND | ND | Fragrance | 5 | 1 | 1 |  |
|  |  |  |  |  |  |  |  | Hair | Beautifies | 5 | 3 | 1 |  |
|  |  |  |  |  |  |  |  |  | Fragrance | 5 | 3 | 1 |  |
|  |  |  |  |  |  |  |  | Place in the house | Fragrance | 5 | 3 | 1 |  |
|  |  |  |  |  |  |  | Not preparation recquired | Placed on the bed | Fragrance | 5 | 4 | 1 |  |
|  |  |  |  |  |  |  |  |  | Fragrance | 5 | 5 | 1 |  |
|  |  |  |  |  |  |  |  |  | Fragrance | 5 | 7 | 1 |  |
|  |  |  |  |  |  |  | Boil the coconut milk until it turns into oil, mix the roots of *Chrysopogon zizanoides* and the flowers of *Pandanus maximus*, let it macerate, and also add the fragrance | Body / skin | Fragrance | 5 | 14 | 1 |  |
|  |  |  |  |  |  |  | Not preparation recquired | Arrange on the bed the flowers of *Pandanus maximus* combined with flowers and inflorescences of *Ocimum* spp. | Fragrance | 5 | 10,12 | 2 |  |
|  |  |  |  |  |  |  |  | Arrange on the bed a mixture of leaves and inflorescences of *Ocimum* spp., flowers of *Pandanus maximus, Jasminum nummulariaefolium*, and *Cananga odorata* | Beautifies | 5 | 23 | 1 |  |
|  |  |  |  |  |  |  |  |  | Fragrance | 5 | 23 | 1 |  |
|  |  |  |  |  |  |  | Crush the leaves and inflorescences of *Ocimum* spp., the rhizome of *Curcuma longa*, the roots of *Chrysopogon zizanoides*, the flowers of *Rosa alba*, *Pandanus maximus, Jasminum nummulariaefolium, Vachellia farnesiana*. Mix everything with the milk and grated fruit of *Cocos nucifera*. Let it marinate for a few minutes or even hours | Exfoliation | Skin care | 2 | 5 | 1 |  |
|  |  |  |  |  |  |  |  |  | Cleanser | 5 | 5 | 1 |  |
|  |  |  |  |  |  |  |  |  | Softens | 2 | 5 | 1 |  |
|  |  |  |  |  |  |  |  |  | Nourishes | 2 | 5 | 1 |  |
|  |  |  |  |  |  |  |  |  | Beautifies | 5 | 5 | 1 |  |
|  |  |  |  |  |  |  |  |  | Hydrates | 2 | 5 | 1 |  |
|  |  |  |  |  |  |  | Crush the rhizome of Curcuma longa and mix it with the scented oil composed of coconut oil, roots of Chrysopogon zizanoides, flowers of Pandanus maximus, Jasminum nummulariaefolium, and fragrant plants from "salika" | Massage (Body) | Cleanser. Caution, turmeric stains | 5 | 5 | 1 |  |
|  |  |  |  |  |  |  |  |  | Beautifies. Caution, turmeric stains | 5 | 5 | 1 |  |
|  |  |  |  |  |  |  | Mix the leaves and inflorescences of *Ocimum* sp. with the flowers of *Pandanus maximus, Rosa alba, Jasminum nummulariaefolium, Cananga odorata*, and roots of *Chrysopogon zizanoides*. Incorporate everything into coconut oil and let it macerate | Body / skin | Beautifies | 5 | 4 | 1 |  |
|  |  |  |  |  |  |  |  |  | Fragrance | 5 | 4 | 1 |  |
|  |  |  |  |  |  |  |  | Face | Beautifies | 5 | 4 | 1 |  |
|  |  |  |  |  |  |  |  |  | Fragrance | 5 | 4 | 1 |  |
|  |  |  |  |  |  |  | Mix all the following plants: roots of *Chrysopogon zizanoides*, leaves and inflorescences of *Ocimum* spp., leaves of *Ayapana triplinervis*, flowers of *Jasminum nummulariaefolium, Pandanus maximus, Cananga odorata*; obtained from safety pins. Introduce them into coconut oil and let them macerate in the oil | Body / skin | Beautifies | 5 | 5 | 1 |  |
|  |  |  |  |  |  |  |  |  | Fragrance | 5 | 5 | 1 |  |
|  |  |  |  |  |  |  |  |  | Softens | 2 | 5 | 1 |  |
|  |  |  |  |  |  |  |  |  | Post-depilatory care | 4 | 5 | 1 |  |
|  |  |  |  |  |  |  | Mix the dried flowers of *Pandanus maximus, Jasminum nummulariaefolium, Cananga odorata,* the dried leaves of *Pogostemon cablin, Ayapana triplinervis*, the dried leaves and inflorescences of *Ocimum* spp., and the dried roots of *Chrysopogon zizanoides* | ND | Fragrance | 5 | 11 | 1 |  |
|  |  |  |  |  |  |  | Mix the flowers of Pandanus maximus with leaves and inflorescences of Ocimum spp. and put them on safety pins | Hair | Beautifies | 5 | 18 | 1 |  |
|  |  |  |  |  |  |  | Mix the flowers of Pandanus maximus with leaves and inflorescences of Ocimum spp. and put them on nackeplace | Neck | Beautifies | 5 | 18 | 1 |  |
|  |  |  |  |  |  |  | Place on the kilabou the leaves and inflorescences of *Ocimum* spp., mixing them with flowers of *Pandanus maximus*. | Hair | Beautifies | 5 | 5,9 | 2 |  |
|  |  |  |  |  |  |  |  |  | Fragrance | 5 | 5,9 | 2 |  |
|  |  |  |  |  |  |  |  | Torso | Beautifies | 5 | 5 | 1 |  |
|  |  |  |  |  |  |  |  |  | Fragrance | 5 | 5 | 1 |  |
|  |  |  |  |  |  |  | Infuse the dried plants, namely: Flowers of *Jasminum nummulariaefolium, Pandanus maximus, Plumeria alba, Cananga odorata, Rosa alba*, the roots of *Chrysopogon zizanoides*, the leaves of *Pogostemon cablin, Ayapana triplinervis*, and the leaves and inflorescence of *Ocimum* spp., in coconut oil | Body / skin | Fragrance | 5 | 10 | 1 |  |
|  |  |  |  |  |  |  | Put the flowers of *Pandanus maximus* on safety pins, combined with flowers and inflorescences of *Ocimum* spp. | Hair | Beautifies | 5 | 10 | 1 |  |
|  |  |  |  |  |  |  |  |  | Fragrance | 5 | 10 | 1 |  |
|  |  |  |  |  |  |  | Put on safety pins a mixture of flowers from *Jasminum nummulariaefolium, Pandanus maximus*, along with leaves and inflorescences of *Ocimum* spp. | Hair | Beautifies | 5 | 12 | 1 |  |
|  |  |  |  |  |  |  |  |  | Fragrance | 5 | 12 | 1 |  |
|  |  |  |  |  |  |  |  | Clothing | Beautifies | 5 | 12 | 1 |  |
|  |  |  |  |  |  |  |  |  | Fragrance | 5 | 12 | 1 |  |
|  |  |  |  |  |  |  | Put on safety pins | Hair | Beautifies | 5 | 21 | 1 |  |
|  |  |  |  |  |  |  | Put on neckelace | Neck | Fragrance | 5 | 21 | 1 |  |
|  |  |  |  |  |  |  | Place on the "kilabou" a mixture of leaves and inflorescences of *Ocimum* spp., the flowers of *Pandanus maximus, Jasminum nummulariaefolium*, and *Cananga odorata* | Hair | Beautifies | 5 | 23 | 1 |  |
|  |  |  |  |  |  |  |  |  | Fragrance | 5 | 23 | 1 |  |
|  |  |  |  |  |  |  | Put the dried leaves and inflorescences of *Ocimum* spp., the dried roots of *Chrysopogon zizanoides*, the dried flowers o*f Pandanus maximus*, and *Jasminum nummulariaefolium* in coconut milk and add the grated fruit | Massage (Body) | Skin care | 2 | 23 | 1 |  |
|  |  |  |  |  |  |  |  |  | Cleanser | 5 | 23 | 1 |  |
|  |  |  |  |  |  |  |  |  | Softens | 2 | 23 | 1 |  |
|  |  |  |  |  |  |  |  |  | Nourishes | 2 | 23 | 1 |  |
|  |  |  |  |  |  |  |  |  | Beautifies | 5 | 23 | 1 |  |
|  |  |  |  |  |  |  |  |  | Hydrates | 2 | 23 | 1 |  |
|  |  |  |  |  |  |  |  |  | Fragrance | 5 | 23 | 1 |  |
|  |  |  |  |  |  |  | Pin the flowers using a safety pin and form a pile on it, creating mixtures to taste with jasmine flowers, Farnese steel, grand vacoa, or leaves and inflorescences of basil | Hair | Beautifies | 5 | 4 | 1 |  |
|  |  |  |  |  |  |  |  |  | Fragrance | 5 | 4 | 1 |  |
|  |  |  |  |  |  |  | Take the mixture of plants obtained from marriages or create your own blend based on plant availability (Roots of *Chrysopogon zizanoides*, Leaves of *Ayapana triplinervis*, Leaves and inflorescences of *Ocimum* spp., Flowers of *Jasminum nummulariaefolium, Vachellia farnesiana, Rosa alba*, "Koukoumba", P*lumeria alba, Cananga odorata, Pandanus maximus*). Once dried, crush and sort them by size. Prepare the coconut oil. To do this, grate the coconuts, extract the milk, let it sit overnight, remove the cream on top, and then cook it in a large pot over a wood fire. Once the oil is ready, add the flower mixture "zoukouba" when the temperature has cooled slightly, and add the pompeia when the oil is no longer very hot. Let it all macerate. The longer the maceration, the more intense the fragrance will be | Body / skin | Nourishes | 2 | 6 | 1 |  |
|  |  |  |  |  |  |  |  |  | Add shine | 3 | 6 | 1 |  |
|  |  |  |  |  |  |  |  |  | Softens | 2 | 6 | 1 |  |
|  |  |  |  |  |  |  |  |  | Fragrans | 5 | 6 | 1 |  |
|  |  |  |  |  |  |  |  |  | Lightens | 3 | 6 | 1 |  |
|  |  |  |  |  |  |  |  |  | Hydrates | 2 | 6 | 1 |  |
|  |  |  |  |  |  |  | Pin the flowers according to the availability and desire of *Jasminum nummulariaefolium, Pandanus maximus, Vachellia farnesiana, Cananga odorata, Rosa alba*, "Koukoumba", *Cestrum nocturnum* using a safety pin and form a pile on it | Hair | Beautifies | 5 | 7 | 1 |  |
|  |  |  |  |  |  |  |  |  | Fragrance | 5 | 7 | 1 |  |
|  |  |  |  |  |  |  | Take the leaves and inflorescences of Ocimum spp., wrap the flowers of Pandanus maximus, and secure everything with threads from a rice sack using a needle | ND | Beautifies | 5 | 12 | 1 |  |
|  |  |  |  |  |  |  |  |  | Fragrance | 5 | 12 | 1 |  |
|  |  |  |  |  |  |  |  |  | Attract luck | 5 | 12 | 1 |  |
|  |  |  |  |  |  |  |  |  | Place on the bed | 5 | 12 | 1 |  |
| *Pedaliaceae* | *Sesamum indicum*  L. | Sésame | Sesame | Pwendzi | Antsiguini |  |  |  |  |  |  |  | NC |
|  |  |  |  |  |  | Seed | Scrape on the coral stone with a little water until obtaining a smooth paste | Face | For dermatoses and allergy pimples that appear on the skin | 1 | 3 | 1 |  |
|  |  |  |  |  |  |  |  | Body / skin |  | 1 | 3 | 1 |  |
|  |  |  |  |  |  |  | ND | Body / skin | Hydrates | 2 | 10 | 1 |  |
|  |  |  |  |  |  |  |  |  | Softens | 2 | 10 | 1 |  |
|  |  |  |  |  |  |  |  |  | Beautifies | 5 | 10 | 1 |  |
|  |  |  |  |  |  |  |  | Face | Hydrates | 2 | 10 | 1 |  |
|  |  |  |  |  |  |  |  |  | Softens | 2 | 10 | 1 |  |
|  |  |  |  |  |  |  |  |  | Beautifies | 5 | 10 | 1 |  |
|  |  |  |  |  |  |  | Crush leaves in water | Shampoo | Cleanser | 4 | 15 | 1 |  |
|  |  |  |  |  |  |  | Crush the leaves of *Lawsonia inermis* and mix with coconut milk and *Sesamum indicum* oil | Massage (Body) | Cleanser | 5 | 16 | 1 |  |
|  |  |  |  |  |  |  |  |  | Brightens | 3 | 16 | 1 |  |
|  |  |  |  |  |  |  |  |  | Add shine | 3 | 16 | 1 |  |
|  |  |  |  |  |  |  |  | Massage (Face) | Cleanser | 5 | 16 | 1 |  |
|  |  |  |  |  |  |  |  |  | Brightens | 3 | 16 | 1 |  |
|  |  |  |  |  |  |  |  |  | Add shine | 3 | 16 | 1 |  |
|  |  |  |  |  |  |  | Crush the leaves of *Lawsonia inermis* and the rhizome of *Curcuma longa*, mix with coconut milk and *Sesamum indicum* oil. | ND | Cleanser | 5 | 16 | 1 |  |
|  |  |  |  |  |  |  |  |  | Brightens | 3 | 16 | 1 |  |
|  |  |  |  |  |  |  |  |  | Add shine | 3 | 16 | 1 |  |
|  |  |  |  |  |  |  |  |  | Cleanser | 5 | 16 | 1 |  |
|  |  |  |  |  |  |  |  |  | Brightens | 3 | 16 | 1 |  |
|  |  |  |  |  |  |  |  |  | Add shine | 3 | 16 | 1 |  |
|  |  |  |  |  |  |  | Crush the leaves of *Lawsonia inermis* and seeds of *Sesamum indicum* together | Face | Cleanser | 5 | 18 | 1 |  |
|  |  |  |  |  |  |  |  | Body / skin | Cleanser | 5 | 18 | 1 |  |
|  |  |  |  |  |  |  | Crush the seeds of *Sesamum indicum* and the leaves of *Lawsonia inermis*, mix | Massage (Body) | Cleanser | 5 | 20 | 1 |  |
|  |  |  |  |  |  |  |  |  | Brightens | 3 | 20 | 1 |  |
|  |  |  |  |  |  |  |  |  | Lightens | 3 | 20 | 1 |  |
|  |  |  |  |  |  |  |  | Massage (Face) | Cleanser | 5 | 20 | 1 |  |
|  |  |  |  |  |  |  |  |  | Brightens | 3 | 20 | 1 |  |
|  |  |  |  |  |  |  |  |  | Lightens | 3 | 20 | 1 |  |
|  |  |  |  |  |  |  | Crush the leaves of *Lawsonia inermis,* crush the rhizome, or add the powder of *Curcuma longa* and *Sesamum indicum* oil | Apply by massaging onto the body, perform small massages, and rinse with lukewarm water | Cleanser | 5 | 26 | 1 |  |
|  |  |  |  |  |  |  |  |  | Beautifies | 5 | 26 | 1 |  |
|  |  |  |  |  |  |  |  |  | Nourishes | 2 | 26 | 1 |  |
|  |  |  |  |  |  |  |  |  | Brightens | 3 | 26 | 1 |  |
|  |  |  |  |  |  |  |  |  | Cleanser | 5 | 26 | 1 |  |
|  |  |  |  |  |  |  |  |  | Beautifies | 5 | 26 | 1 |  |
|  |  |  |  |  |  |  |  |  | Nourishes | 2 | 26 | 1 |  |
|  |  |  |  |  |  |  |  |  | Brightens | 3 | 26 | 1 |  |
|  |  |  |  |  |  |  | Crush the leaves of *Lawsonia inermis* and the rhizome of *Curcuma longa*, mix with S*esamum indicum* oil | Exfoliation | Cleanser | 5 | 32 | 1 |  |
|  |  |  |  |  |  |  |  |  | Brightens | 3 | 32 | 1 |  |
|  |  |  |  |  |  |  |  |  | Anti-aging | 2 | 32 | 1 |  |
|  |  |  |  |  |  |  |  |  | Softens | 2 | 32 | 1 |  |
|  |  |  |  |  |  |  |  |  | Firms | 5 | 32 | 1 |  |
|  |  |  |  |  |  |  |  |  | Lightens | 3 | 32 | 1 |  |
|  |  |  |  |  |  |  | Crush the seeds of *Sesamum indicum* and the leaves of *Lawsonia inermis*, and mix | Exfoliation (face) | Cleanser | 5 | 34 | 1 |  |
|  |  |  |  |  |  |  |  |  | Lightens | 3 | 34 | 1 |  |
| *Piperaceae* | *Piper nigrum* L. | Poivrier noir | Black pepper | Vilivili | Vilivili |  |  |  |  |  |  |  | ODAM059 |
|  |  |  |  |  |  | Seed | Crush the chives or green onion leaves (*Allium schoenoprasum* or garlic *Allium* spp.) and black pepper seeds until obtaining a puree | Vagina | Clean the vagina | 5 | 18 | 5 |  |
|  |  |  |  |  |  |  |  |  | Anti-itch (vagina) | 5 | 18 | 5 |  |
| *Poacea* | *Chrysopogon zizanioides* (L.) Roberty | Vétiver, chiendent odorant | vetiver | Manu kantru | Kotuvera |  |  |  |  |  |  |  | ODAM060 |
|  |  |  |  |  |  | Root | Mix the roots with coconut oil and put to macerate | Body / skin | Fragrance | 5 | 3 | 1 |  |
|  |  |  |  |  |  |  | Mix the leaves and inflorescences of *Ocimum* sp. with the flowers of *Pandanus maximus, Rosa alba, Jasminum nummulariaefolium, Cananga odorata*, and roots of *Chrysopogon zizanoides*. Incorporate everything into Coconut (Cocos nucifera) oil and let it macerate | Body / skin | Beautifies | 5 | 4 | 1 |  |
|  |  |  |  |  |  |  |  |  | Fragrance | 5 | 4 | 1 |  |
|  |  |  |  |  |  |  |  | Face | Beautifies | 5 | 4 | 1 |  |
|  |  |  |  |  |  |  |  |  | Fragrance | 5 | 4 | 1 |  |
|  |  |  |  |  |  |  | Crush the rhizome of *Curcuma longa*, the leaves of *Lawsonia inermis*, and the roots of *Chrysopogon zizanoides*. Mix everything with coconut (*Cocos nucifera*) milk and oil. Let it macerate for a few minutes or hours | Exfoliation | Cleanser | 5 | 4 | 1 |  |
|  |  |  |  |  |  |  |  |  | Beautifies | 5 | 4 | 1 |  |
|  |  |  |  |  |  |  |  |  | Hydrates | 2 | 4 | 1 |  |
|  |  |  |  |  |  |  |  |  | Softens | 2 | 4 | 1 |  |
|  |  |  |  |  |  |  | Crush the leaves and inflorescences of *Ocimum* spp., the rhizome of *Curcuma longa*, the roots of *Chrysopogon zizanoides,* the flowers of *Rosa alba, Pandanus maximus, Jasminum nummulariaefolium*, and Vachellia farnesiana. Mix everything with coconut (*Cocos nucifera*) milk and grated fruit. Let it macerate for a few minutes or hours | Exfoliation | Skin care | 2 | 5 | 1 |  |
|  |  |  |  |  |  |  |  |  | Cleanser | 5 | 5 | 1 |  |
|  |  |  |  |  |  |  |  |  | Softens | 2 | 5 | 1 |  |
|  |  |  |  |  |  |  |  |  | Nourishes | 2 | 5 | 1 |  |
|  |  |  |  |  |  |  |  |  | Beautifies | 5 | 5 | 1 |  |
|  |  |  |  |  |  |  |  |  | Hydrates | 2 | 5 | 1 |  |
|  |  |  |  |  |  |  | Mix all the following plants: the roots of *Chrysopogon zizanoides*, leaves and inflorescences of *Ocimum* spp., leaves of A*yapana triplinervis*, flowers of *Jasminum nummulariaefolium*, *Pandanus maximus, Cananga odorata*; obtained from safety pins. Introduce them into coconut (*Cocos nucifera*) oil and let it macerate in the oil | Body / skin | Beautifies | 5 | 5 | 1 |  |
|  |  |  |  |  |  |  |  |  | Fragrance | 5 | 5 | 1 |  |
|  |  |  |  |  |  |  |  |  | Softens | 2 | 5 | 1 |  |
|  |  |  |  |  |  |  |  |  | Post-depilatory care | 3 | 5 | 1 |  |
|  |  |  |  |  |  |  | Take the mixture of plants obtained from marriages or create your own blend based on plant availability (Roots of *Chrysopogon zizanoides*, Leaves of *Ayapana triplinervis*, Leaves and inflorescences of *Ocimum* spp., Flowers of *Jasminum nummulariaefolium, Vachellia farnesiana, Rosa alba*, "Koukoumba", P*lumeria alba, Cananga odorata, Pandanus maximus*). Once dried, crush and sort them by size. Prepare the coconut oil. To do this, grate the coconuts, extract the milk, let it sit overnight, remove the cream on top, and then cook it in a large pot over a wood fire. Once the oil is ready, add the flower mixture "zoukouba" when the temperature has cooled slightly, and add the pompeia when the oil is no longer very hot. Let it all macerate. The longer the maceration, the more intense the fragrance will be | Body / skin | Nourishes | 2 | 6 | 1 |  |
|  |  |  |  |  |  |  |  |  | Add shine | 3 | 6 | 1 |  |
|  |  |  |  |  |  |  |  |  | Softens | 2 | 6 | 1 |  |
|  |  |  |  |  |  |  |  |  | Fragrans | 5 | 6 | 1 |  |
|  |  |  |  |  |  |  |  |  | Lightens | 3 | 6 | 1 |  |
|  |  |  |  |  |  |  |  |  | Hydrates | 2 | 6 | 1 |  |
|  |  |  |  |  |  |  | Crush the rhizome of *Curcuma longa* and mix it with the scented oil composed of coconut *(Cocos nucifera*) oil, the roots of *Chrysopogon zizanoides*, the flowers of *Pandanus maximus, Jasminum nummulariaefolium,* and fragrant plants from "Salika" | Massage (body) then wash | Cleanser | 5 | 9 | 1 |  |
|  |  |  |  |  |  |  |  |  | Beautifies | 5 | 9 | 1 |  |
|  |  |  |  |  |  |  | Put the dried plants, namely: Flowers of J*asminum nummulariaefolium, Pandanus maximus, Plumeria alba, Cananga odorata, Rosa alba,* the roots of *Chrysopogon zizanoides*, the leaves of *Pogostemon cablin, Ayapana triplinervis,* and the leaves and inflorescence of *Ocimum* spp., in decoction in coconut (*Cocos nucifera*) oil | Body / skin | Fragrance | 5 | 10 | 1 |  |
|  |  |  |  |  |  |  | Mingle the dried flowers of *Pandanus maximus, Jasminum nummulariaefolium, Cananga odorata*, the dried leaves of *Pogostemon cablin, Ayapana triplinervis,* the dried leaves and inflorescences of *Ocimum* spp., and the root of *Chrysopogon zizanoides* | ND | Fragrance | 5 | 11 | 1 |  |
|  |  |  |  |  |  |  | When the oil of *Cocos nucifera* is cooked, take the dried roots of *Chrysopogon zizanoides*, crush them, and put them in the oil, mix, and let it macerate for about two weeks | Massage (body) | Fragrance | 5 | 12 | 1 |  |
|  |  |  |  |  |  |  |  |  | Softens | 2 | 12 | 1 |  |
|  |  |  |  |  |  |  |  |  | Soothes | 2 | 12 | 1 |  |
|  |  |  |  |  |  |  | Cook the coconut milk until it turns into oil and mix it with the roots of *Chrysopogon zizanoides* and the flowers of *Pandanus maximus*. Let it macerate and add fragrance as well | Body / skin | Fragrance | 5 | 14 | 1 |  |
|  |  |  |  |  |  |  | Crush the leaves of *Lawsonia inermis,* the roots of *Chrysopogon zizanoides,* and the rhizome of *Curcuma longa*. Mix everything with the oil and milk of *Cocos nucifera*. | Exfoliation | Cleanser | 5 | 23 | 1 |  |
|  |  |  |  |  |  |  |  |  | Beautifies | 5 | 23 | 1 |  |
|  |  |  |  |  |  |  |  |  | Hydrates | 2 | 23 | 1 |  |
|  |  |  |  |  |  |  |  |  | Softens | 2 | 23 | 1 |  |
|  |  |  |  |  |  |  | Put the dried leaves and inflorescences of *Ocimum* spp., the dried roots of *Chrysopogon zizanoides*, the dried flowers of *Pandanus maximus*, and *Jasminum nummulariaefolium* in *Cocos nucifera* milk, and add the grated fruit | Massage (body) | Skin care | 2 | 23 | 1 |  |
|  |  |  |  |  |  |  |  |  | Cleanser | 5 | 23 | 1 |  |
|  |  |  |  |  |  |  |  |  | Softens | 2 | 23 | 1 |  |
|  |  |  |  |  |  |  |  |  | Nourishes | 2 | 23 | 1 |  |
|  |  |  |  |  |  |  |  |  | Beautifies | 5 | 23 | 1 |  |
|  |  |  |  |  |  |  |  |  | Hydrates | 2 | 23 | 1 |  |
|  |  |  |  |  |  |  |  |  | Fragrance | 5 | 23 | 1 |  |
| *Polypodiaceae* | *Phymatosorus scolopendria* (Burm.f.) Pic.Serm. | Scolopendre, Patte lézard | ND | M'hono béni | Kangadja sampa ravihi |  |  |  |  |  |  |  | NC |
|  |  |  |  |  |  | Leaf | Dry and sift the leaves of *Phymatosorus scolopendria* and the flowers of J*asminum nummulariaefolium*. Put them in coconut oil *(Cocos nucifera* oil) and let it macerate. | Exfoliation | Hydrates | 2 | 19 | 1 |  |
|  |  |  |  |  |  |  |  |  | Cleanser | 5 | 19 | 1 |  |
|  |  |  |  |  |  |  |  |  | Beautifies | 5 | 19 | 1 |  |
| *Pteridaceae* | *Pityrogramma calomelanos* (L.) Link | Fougère d'argent | ND | ND | Kangadja orimbo vihavy |  |  |  |  |  |  |  | NC |
|  |  |  |  |  |  | Leaf | Not preparation recquired | Apply the underside of the leaves on the face. This allows for patterns to be created due to the bloom present on the back of the leaves | Face makeup, during ceremonies and weddings | 5 | 7 | 1 |  |
|  |  |  |  |  |  |  |  | Apply the underside of the leaves with the bloom on the face. This allows for a pattern to be created from the powder | Make up | 5 | 18,19 | 2 |  |
| *Rosaceae* | *Rosa* sp. | Rosier | White rose | Maouwa | Mauwa |  |  |  |  |  |  |  | ODAM061 |
|  |  |  |  |  |  | Flower | Not preparation recquired | Place on the bed | Fragrance | 5 | 4,5,7,32 | 4 |  |
|  |  |  |  |  |  |  | Cook the petals of *Rosa alba* in *Cocos nucifera* oil | Body / skin | Beautifies | 5 | 18 | 1 |  |
|  |  |  |  |  |  |  | Not preparation recquired | Place a mixture of *Rosa alba* and *Jasminum nummulariaefolium* flowers on the bed | Fragrance | 5 | 11 | 1 |  |
|  |  |  |  |  |  |  | Not preparation recquired | Place a mixture of *Rosa alba, Jasminum nummulariaefolium,* and *Vachellia farnesiana* flowers on the bed | Fragrance | 5 | 11 | 1 |  |
|  |  |  |  |  |  |  | Not preparation recquired | Place on the bed a combination of *Rosa alba* and J*asminum nummulariaefolium* flowers, along with V*achellia farnesiana*, and add leaves and inflorescences of *Ocimum* spp. | Fragrance | 5 | 11 | 1 |  |
|  |  |  |  |  |  |  | Crush the leaves and inflorescences of *Ocimum* spp., the rhizome of *Curcuma longa*, the roots of *Chrysopogon zizanoides*, the flowers of *Rosa alba, Pandanus maximus, Jasminum nummulariaefolium*, and *Vachellia farnesiana*. Mix everything with the grated fruit and coconut milk (lait de *Cocos nucifera*). Let it macerate for a few minutes or hours. | Exfoliation | Skin care | 2 | 5 | 1 |  |
|  |  |  |  |  |  |  |  |  | Cleanser | 5 | 5 | 1 |  |
|  |  |  |  |  |  |  |  |  | Softens | 2 | 5 | 1 |  |
|  |  |  |  |  |  |  |  |  | Nourishes | 2 | 5 | 1 |  |
|  |  |  |  |  |  |  |  |  | Beautifies | 5 | 5 | 1 |  |
|  |  |  |  |  |  |  |  |  | Hydrates | 2 | 5 | 1 |  |
|  |  |  |  |  |  |  | Create a necklace with a mixture of *Jasminum nummulariaefolium flowers, Plumeria alba, Cananga odorata, Bougainvillea spectabilis,* and leaves of *Pogostemon cablin,* as well as leaves and inflorescences of *Ocimum* spp. | Neck | Beautifies | 5 | 11 | 1 |  |
|  |  |  |  |  |  |  |  |  | Fragrance | 5 | 11 | 1 |  |
|  |  |  |  |  |  |  | Make a mixture of *Jasminum nummulariaefolium* flowers, *Rosa alba,* and *Vachellia farnesiana*. Put them on hairpins | Hair | Beautifies | 5 | 16 | 1 |  |
|  |  |  |  |  |  |  | Create a mixture of *Jasminum nummulariaefolium* flowers, *Rosa alba*, and *Vachellia farnesiana*. Place them on a necklace (toka) | Neck | Beautifies | 5 | 16 | 1 |  |
|  |  |  |  |  |  |  | Mix the leaves and inflorescences of *Ocimum* sp. with the flowers of *Pandanus maximus, Rosa alba, Jasminum nummulariaefolium, Canaga odorata*, and the roots of *Chrysopogon zizanoides.* Incorporate everything into *Cocos nucifera* oil and let it macerate | Face | Fragrance | 5 | 4 | 1 |  |
|  |  |  |  |  |  |  |  |  | Beautifies | 5 | 4 | 1 |  |
|  |  |  |  |  |  |  |  | Body / skin | Fragrance | 5 | 4 | 1 |  |
|  |  |  |  |  |  |  |  |  | Beautifies | 5 | 4 | 1 |  |
|  |  |  |  |  |  |  | Put the flowers to macerate in water and store in a cool place | Face | Refreshes | 5 | 8 | 1 |  |
|  |  |  |  |  |  |  |  |  | Revives | 5 | 8 | 1 |  |
|  |  |  |  |  |  |  | Apply on the face, especially in the evening and upon waking up. | ND | ND | ND | 14 | ND |  |
|  |  |  |  |  |  |  | Boil the dried plants, including flowers of *Jasminum nummulariaefolium, Pandanus maximus, Plumeria alba, Cananga odorata, Rosa alba*, the roots of *Chrysopogon zizanoides*, leaves of *Pogostemon cablin, Ayapana triplinervis*, and leaves and inflorescence of *Ocimum* spp., in *Cocos nucifera* oil | Body / skin | Fragrance | 5 | 10 | 1 |  |
|  |  |  |  |  |  |  | Put the flowers on safety pins | Hair | Beautifies | 5 | 18 | 1 |  |
|  |  |  |  |  |  |  | Put the flowers on necklaces or make necklaces with them | Neck | Beautifies | 5 | 18 | 1 |  |
|  |  |  |  |  |  |  | Decoction | Face | Refreshes | 5 | 18 | 1 |  |
|  |  |  |  |  |  |  |  |  | Fragrance | 5 | 18 | 1 |  |
|  |  |  |  |  |  |  | Place the leaves and inflorescences of *Ocimum* spp. on the kilabou, mixing them with flowers of *Jasminum nummulariaefolium* and *Rosa alba* | Hair | Fragrance | 5 | 5 | 1 |  |
|  |  |  |  |  |  |  |  |  | Beautifies | 5 | 5 | 1 |  |
|  |  |  |  |  |  |  |  | Torso | Fragrance | 5 | 5 | 1 |  |
|  |  |  |  |  |  |  |  |  | Beautifies | 5 | 5 | 1 |  |
|  |  |  |  |  |  |  | Put on hairpins | Hair | Fragrance | 5 | 9 | 1 |  |
|  |  |  |  |  |  |  |  |  | Beautifies | 5 | 9 | 1 |  |
|  |  |  |  |  |  |  | Place a mixture of flowers from J*asminum nummulariaefolium, Vachellia farnesiana, Rosa alba,* and the leaves and inflorescences of *Ocimum* spp. on the safety pins. | Hair | Fragrance | 5 | 10 | 1 |  |
|  |  |  |  |  |  |  |  |  | Beautifies | 5 | 10 | 1 |  |
|  |  |  |  |  |  |  | Place a mixture of flowers from *Rosa alba* and *Jasminum nummulariaefolium* on safety pins | Hair | Fragrance | 5 | 11 | 1 |  |
|  |  |  |  |  |  |  |  |  | Beautifies | 5 | 11 | 1 |  |
|  |  |  |  |  |  |  |  | Clothing | Fragrance | 5 | 11 | 1 |  |
|  |  |  |  |  |  |  |  |  | Beautifies | 5 | 11 | 1 |  |
|  |  |  |  |  |  |  | Place a mixture of flowers from *Rosa alba, Jasminum nummulariaefolium*, and *Vachellia farnesiana* on safety pins | Hair | Fragrance | 5 | 11 | 1 |  |
|  |  |  |  |  |  |  |  |  | Beautifies | 5 | 11 | 1 |  |
|  |  |  |  |  |  |  |  | Clothing | Fragrance | 5 | 11 | 1 |  |
|  |  |  |  |  |  |  |  |  | Beautifies | 5 | 11 | 1 |  |
|  |  |  |  |  |  |  | Place a mixture of flowers from *Rosa alba* and *Jasminum nummulariaefolium*, along with *Vachellia farnesiana*, on safety pins, and incorporate leaves and inflorescences from *Ocimum* spp. | Hair | Fragrance | 5 | 11 | 1 |  |
|  |  |  |  |  |  |  |  |  | Beautifies | 5 | 11 | 1 |  |
|  |  |  |  |  |  |  |  | Clothing | Fragrance | 5 | 11 | 1 |  |
|  |  |  |  |  |  |  |  |  | Beautifies | 5 | 11 | 1 |  |
|  |  |  |  |  |  |  | Attach a mixture of leaves and inflorescences from *Ocimum* spp. and J*asminum nummulariaefolium* to safety pins | Hair | Fragrance | 5 | 32 | 1 |  |
|  |  |  |  |  |  |  |  |  | Beautifies | 5 | 32 | 1 |  |
|  |  |  |  |  |  |  |  | Clothing | Fragrance | 5 | 32 | 1 |  |
|  |  |  |  |  |  |  |  |  | Beautifies | 5 | 32 | 1 |  |
|  |  |  |  |  |  |  | String a mixture of leaves and inflorescences from *Ocimum* spp. and *Jasminum nummulariaefolium* on a necklace | Neck | Fragrance | 5 | 32 | 1 |  |
|  |  |  |  |  |  |  |  |  | Beautifies | 5 | 32 | 1 |  |
|  |  |  |  |  |  |  | Pierce the flowers using a needle and thread them onto a string made from a rice sack. Tie it all together. This is used in the making of a necklace | Neck | Beautifies | 5 | 2 | 1 |  |
|  |  |  |  |  |  |  | Pierce the flowers using a pin and form a pile with them | Clothing | Beautifies | 5 | 2 | 1 |  |
|  |  |  |  |  |  |  |  | Hair | Beautifies | 5 | 2 | 1 |  |
|  |  |  |  |  |  |  |  |  | Beautifies | 5 | 4 | 1 |  |
|  |  |  |  |  |  |  |  |  | Fragrance | 5 | 4 | 1 |  |
|  |  |  |  |  |  |  | Take the mixture of plants obtained from marriages or create your own blend based on plant availability (Roots of *Chrysopogon zizanoides*, Leaves of *Ayapana triplinervis*, Leaves and inflorescences of *Ocimum* spp., Flowers of *Jasminum nummulariaefolium, Vachellia farnesiana, Rosa alba*, "Koukoumba", P*lumeria alba, Cananga odorata, Pandanus maximus*). Once dried, crush and sort them by size. Prepare the coconut oil. To do this, grate the coconuts, extract the milk, let it sit overnight, remove the cream on top, and then cook it in a large pot over a wood fire. Once the oil is ready, add the flower mixture "zoukouba" when the temperature has cooled slightly, and add the pompeia when the oil is no longer very hot. Let it all macerate. The longer the maceration, the more intense the fragrance will be | Body / skin | Nourishes | 2 | 6 | 1 |  |
|  |  |  |  |  |  |  |  |  | Add shine | 3 | 6 | 1 |  |
|  |  |  |  |  |  |  |  |  | Softens | 2 | 6 | 1 |  |
|  |  |  |  |  |  |  |  |  | Fragrans | 5 | 6 | 1 |  |
|  |  |  |  |  |  |  |  |  | Lightens | 3 | 6 | 1 |  |
|  |  |  |  |  |  |  |  |  | Hydrates | 2 | 6 | 1 |  |
|  |  |  |  |  |  |  | Pierce the flowers of Jasminum nummulariaefolium, Pandanus maximus, Vachellia farnesiana, Canaga odorata, Rosa alba, Koukoumba, Cestrum nocturnum using a pin, and form a pile with them | Hair | Beautifies | 5 | 7 | 1 |  |
|  |  |  |  |  |  |  |  |  | Fragrance | 5 | 7 | 1 |  |
|  |  |  |  |  |  |  | ND | ND | Fragrance | 5 | 9 | 1 |  |
| *Rubiaceae* | *Guettarda speciosa* L. | Bois cassant bord-de-mer | ND | Fu m'tsanga | Fu m'tsanga |  |  |  |  |  |  |  | *ODAM011* |
|  |  |  |  |  |  | Flower | Not preparation recsuired | ND | Fragrance | 5 | 9 | 1 |  |
|  |  |  |  |  |  | Piece of wood | Scrape the piece of wood on the dead coral stone with a little water until obtaining a smooth paste | Apply to the pimples caused by food allergies or other reasons | Anti-pimples | 1 | 1,1,9,9,13,13,28 | 7 |  |
|  |  |  |  |  |  |  | Crush the young or yellowed leaves of *Guettarda speciosa* and put them in coconut oil *(Cocos nucifera* oil) | Body / skin | Anti-pimples | 1 | 18,26 | 2 |  |
| *Rubiaceae* | *Ixora cremixora* Drake |  |  | Mianga nianga | Sari mapwera marachi |  |  |  |  |  |  |  | *NC* |
|  |  |  |  |  |  | Flower | Put flower on hairpins | Hair | Beautifies | 5 | 18 | 1 |  |
|  |  |  |  |  |  |  | Putt flower on Necklace | Neck | Beautifies | 5 | 18 | 1 |  |
| *Rutaceae* | *Citrus aurantium* L. | Oranger | Sweet orange | Mavoubara | Tsoha mami |  |  |  |  |  |  |  | NC |
|  |  |  |  |  |  | Fruit | Sweat the skin of the fruit | Rub under the armpits | Deodorant | 1 | 9 | 1 |  |
|  |  |  |  |  |  |  | Remove the skin and crush in the hands until sweating. | Hair | Fragrance | 5 | 14 | 1 |  |
| *Rutaceae* | *Citrus medica* L. | Cédratier | Citron | Murundra kapu | Tsoha kapo |  |  |  |  |  |  |  | NC |
|  |  |  |  |  |  | Leaf | Decoction | Wash the vagina | Reduce bad odors | 5 | 15 | 1 |  |
| *Rutaceae* | *Citrus* sp. | Citronnier | ND | Djimogné | Tsoha foyi |  |  |  |  |  |  |  | ODAM062 |
|  |  |  |  |  |  | Fruit (juice) | Cut the fruit in half | Rub under the armpits, let it sit, and rinse with water | Deodorant | 1 | 1,2,3,24 | 4 |  |
|  |  |  |  |  |  |  |  | Rub under the armpits | Deodorant | 1 | 4,5,8,10,11, 23,33 | 7 |  |
|  |  |  |  |  |  |  |  |  | Antiperspirant | 1 | 33 | 1 |  |
|  |  |  |  |  |  |  |  | Apply the juice of the fruit under the armpits by rubbing | Deodorant | 1 | 13,15,16 | 3 |  |
|  |  |  |  |  |  |  |  |  | Antiperspirant | 1 | 13 | 1 |  |
|  |  |  |  |  |  |  | Crush leaves of *Lawsonia inermis* and add lemon juice from *Citrus* sp | Apply to the soles of the feet and the heels | Heal the cracks | 4 | 2,2,32,32 | 4 |  |
|  |  |  |  |  |  |  |  | Apply on the hands, creating patterns. Let it dry and leave it on for a few minutes or hours, then rinse | Beautifies | 5 | 17 | 1 |  |
|  |  |  |  |  |  |  |  | Apply on the feet, creating patterns. Let it dry and leave it on for a few minutes or hours, then rinse | Beautifies | 5 | 17 | 1 |  |
|  |  |  |  |  |  |  |  | Apply on the hands | Beautifies | 5 | 32,33 | 2 |  |
|  |  |  |  |  |  |  |  | Apply on the feet | Beautifies | 5 | 32,33 | 2 |  |
|  |  |  |  |  |  |  | Crush the rhizome of *Curcuma longa* and the leaves of *Lawsonia inermis*, add the juice of *Citrus* sp., and mix everything | Apply on the hands while making designs | Beautifies | 5 | 27 | 1 |  |
|  |  |  |  |  |  |  |  | Apply on the feet while making designs | Beautifies | 5 | 27 | 1 |  |
|  |  |  |  |  |  |  |  | Apply to hands while drawing designs. Leave for a few hours, then rinse | Beautifies | 5 | 16,16 | 2 |  |
|  |  |  |  |  |  |  | Crush the leaves of *Lawsonia inermis*, add the juice of *Citrus* sp, and mix. Leave for a few minutes | Apply on the skin and leave on for a few minutes to a few hours | Color skin | 3 | 13 | 1 |  |
|  |  |  |  |  |  |  |  |  | Beautifies | 5 | 13 | 1 |  |
|  |  |  |  |  |  |  |  | Apply on the nails and leave on for a few minutes to a few hours | Color nails | 3 | 13 | 1 |  |
|  |  |  |  |  |  |  |  |  | Beautifies | 5 | 13 | 1 |  |
|  |  |  |  |  |  |  |  | Hands | Beautifies | 5 | 5, 26,33 | 3 |  |
|  |  |  |  |  |  |  |  | Feet | Beautifies | 5 | 5, 26,33 | 3 |  |
|  |  |  |  |  |  |  |  | Apply on hands and feet. Leave for a few hours, then rinse. | Beautifies | 5 | 10,10, | 2 |  |
|  |  |  |  |  |  |  |  | Apply to the soles of the feet and the heels | Heal the cracks | 4 | 13,13,15,15 | 4 |  |
|  |  |  |  |  |  |  |  | Draw designs on the feet, leave for a few minutes or hours, then rinse and apply body lotion or oil | Beautifies | 5 | 15,15 | 2 |  |
|  |  |  |  |  |  |  | Crush the leaves of *Lawsonia inermis*, add the juice of *Citrus* sp. Possibility to add Turmeric (*Curcuma longa*) according to taste | Apply using a matchstick on the skin, hands, and feet to create designs. | Beautifies | 5 | 6 | 1 |  |
|  |  |  |  |  |  |  | Crush the leaves of *Lawsonia inermis* until they become soft. Add the juice of *Citrus* sp. | Massage (face) | Cleanser. Do not go in the sun and be careful not to leave it on the face as it may cause discoloration, and avoid the eye area due to the presence of lemon | 5 | 9 | 1 |  |
|  |  |  |  |  |  |  |  |  | Tanned complexion. Do not go in the sun and be careful not to leave it on the face as it may cause discoloration, and avoid the eye area due to the presence of lemon | 3 | 9 | 1 |  |
|  |  |  |  |  |  |  | Crush the leaves of *Lawsonia inermis* and add the juice of *Citrus* sp. Then apply coconut oil (*Cocos nucifera*) after rinsing | Apply on the soles of the feet and toenails | Beautifies | 5 | 8 | 1 |  |
|  |  |  |  |  |  |  |  | Apply on the fingertips, nails, and palms of the hands | Beautifies | 5 | 8 | 1 |  |
|  |  |  |  |  |  |  | Crush the leave of "romba" with *Citrus s*p | Massage (body) and wash | Stimulates | 5 | 20 | 1 |  |
|  |  |  |  |  |  |  |  |  | Tones | 5 | 20 | 1 |  |
|  |  |  |  |  |  |  |  |  | Energyzing | 5 | 20 | 1 |  |
|  |  |  |  |  |  |  | Crush the seeds of *Piper nigrum*, mix them with honey and the juice of *Citrus* sp. | Apply in the mouth, scraping until bleeding, then spit or swallow | Pimples on the tongue causing small sores | 1 | 28 | 1 |  |
|  |  |  |  |  |  |  | Tap the fruit to extract the juice | Rub under the armpits | Deodorant | 1 | 17 | 1 |  |
|  |  |  |  |  |  |  |  |  | Antiperspirant | 1 | 17 | 1 |  |
|  |  |  |  |  |  | Fruit (peel) | Sweat the skin of the fruit | Rub under the armpits | Deodorant | 1 | 9 | 1 |  |
| *Rutaceae* | *Murraya paniculata* (L.) Jack | Oranger jasmin | orange jessamine | Fulagin | Anfololo |  |  |  |  |  |  |  | *ODAM015* |
|  |  |  |  |  |  | Flower | Pierce the flowers using a safety pin and create a pile with it | Hair | Beautifies | 5 | 4 | 1 |  |
|  |  |  |  |  |  |  |  |  | Fragrance | 5 | 4 | 1 |  |
|  |  |  |  |  |  |  | Not preparation recsuired | Place on bed | Fragrance | 5 | 4,32 | 2 |  |
|  |  |  |  |  |  |  | Put flower on hairpins | Hair | Beautifies | 5 | 32 | 1 |  |
|  |  |  |  |  |  |  |  |  | Fragrance | 5 | 32 | 1 |  |
|  |  |  |  |  |  |  |  | Clothing | Beautifies | 5 | 32 | 1 |  |
|  |  |  |  |  |  |  |  |  | Fragrance | 5 | 32 | 1 |  |
|  |  |  |  |  |  |  | Put on necklace |  | Beautifies | 5 | 32 | 1 |  |
|  |  |  |  |  |  |  |  |  | Fragrance | 5 | 32 | 1 |  |
| *Sapindaceae* | *Litchi chinensis* Sonn. | Litchi de Chine | Lychee | Litshi | Litshi |  |  |  |  |  |  |  | ODAM064 |
|  |  |  |  |  |  | Piece of wood | Scrape the piece of wood on the dead coral stone with a little water until obtaining a smooth paste | Face | Sun protection | 3 | 10 | 1 |  |
| *Sapindaceae* | *Sapindus saponaria* L. | Arbre à savon | Soap tree | Arita | Sabon kakazo |  |  |  |  |  |  |  | ODAM063 |
|  |  |  |  |  |  | Leaf | Crush the leaves in cold water in a large bucket | Wash | Relieves heat rash on the body that itches | 1 | 14 | 1 |  |
|  |  |  |  |  |  | Seed | Crush the seeds | Rub on the body in case of irritation with bumps | Reduces irritation. Caution: Irritating if in contact with eyes. | 1 | 16 | 1 |  |
|  |  |  |  |  |  |  |  |  | Anti-pimples. Caution: Irritating if in contact with eyes. | 1 | 16 | 1 |  |
|  |  |  |  |  |  |  |  |  | Treats irritations and body rashes, such as chickenpox, etc; Caution: Irritating if in contact with eyes. | 1 | 35 | 1 |  |
|  |  |  |  |  |  |  | Crush the seeds of *Sapindus saponaria* and add the latex of "Adabu" | Rub on the body in case of irritation with bumps | Anti-pimples. Caution: Irritating if in contact with eyes. | 1 | 18, 20 | 2 |  |
| *Sapotaceae* | *Gambeya boiviniana* Pierre |  |  |  | Famelho |  |  |  |  |  |  |  | NC |
|  |  |  |  |  |  | Leaf | Crush the seeds of *Commiphora arafy* and the leaves of *Gambeya boiviniana* and put them in water | Mouthwash | Toothaches | 5 | 35 | 1 |  |
| *Solanaceae* | *Cestrum nocturnum* L. | Jasmin de nuit | Lady of the Night | Anfu ya huku | Sirya huku |  |  |  |  |  |  |  | *ODAM011* |
|  |  |  |  |  |  | Flower | Pierce the flowers using a safety pin and create a pile with it | Hair | Beautifies | 5 | 4 | 1 |  |
|  |  |  |  |  |  |  |  |  | Fragrance | 5 | 4 | 1 |  |
|  |  |  |  |  |  |  | Not preparation recsuired | Place on bed | Fragrance | 5 | 4,7 | 2 |  |
|  |  |  |  |  |  |  | Pierce the flowers, according to the availability and desire, of J*asminum nummulariaefolium, Pandanus maximus, Vachellia farnesiana, Cananga odorata, Rosa alba,* "Koukoumba", *Cestrum nocturnum* using a safety pin and form a pile with them. | Hair | Beautifies | 5 | 7 | 1 |  |
|  |  |  |  |  |  |  |  |  | Fragrance | 5 | 7 | 1 |  |
|  |  |  |  |  |  |  | ND | ND | Fragrance | 5 | 9 | 1 |  |
| *Solanaceae* | *Solanum melongena* L. | Aubergine | Eggplant | Bégani | Bégani |  |  |  |  |  |  |  |  |
|  |  |  |  |  |  | Fruit | Remove the skin | Rub on the areas of the feet with calluses and cracks. Take the leaves, put them in a siniha, and walk on them for about 20 minutes, then remove the feet | Heal the cracks | 4 | 9 | 1 |  |
| *Solanaceae* | *Solanum richardii* Dunal | ND | Eggpplant | ND | Sari anguivi |  |  |  |  |  |  |  | ODAM021 |
|  |  |  |  |  |  | Leaf | Crush the leaves and put them in water | Mouthwash | Toothaches | 5 | 35 | 1 |  |
| *Verbenaceae* | *Lantana trifolia* L. |  |  | M'bwasera | Sari fatsiki madani |  |  |  |  |  |  |  |  |
|  |  |  |  |  |  | Leaf | Crush the leaves of *Ocimum gratissimum*, *Lantana trifolia,* and the rhizome of *Zingiber officinale*, and form a ball | Insert the ball into the vagina and position yourself above a heated stone | Tightens | 5 | 15 | 1 |  |
|  |  |  |  |  |  |  |  |  | Tones | 5 | 15 | 1 |  |
| *Verbenaceae* | *Stachytarpheta urticifolia* (Salibs.) Sims. | Verveine bleu | ND | M'ri wagwegwe | Mshari |  |  |  |  |  |  |  | ODAM023 |
|  |  |  |  |  |  | Leaf | Crush the leaves in water | Intimate hygiene | Intimate hygiene | 5 | 2 | 1 |  |
|  |  |  |  |  |  |  | Crush the leaves until sweating | Apply as a poultice on skin blemishes | Anti spot | 1 | 3 | 1 |  |
|  |  |  |  |  |  |  | Decoction | Cleanser vagina | Antifungal | 1 | 34 | 1 |  |
|  |  |  |  |  |  |  |  |  | Intimate hygiene | 5 | 34 | 1 |  |
| *Vitaceae* | *Leea guineensis* G.Don | ND | ND | ND | Sadrakidraki |  |  |  |  |  |  |  |  |
|  |  |  |  |  |  | Root | Scrape the roots of "Vahi tampa", "sadrakidraki", and "pouropetka" on dead coral stone with a little water until a paste is obtained | Apply on the pimples | Anti pimples | 1 | 20 | 1 |  |
|  |  |  |  |  |  |  | Crush the bark into powder with a little water | Apply on wound | Accelerates wound healing | 1 | 33 | 1 |  |
|  |  |  |  |  |  |  |  | Apply on fungal infection | Anti fungal | 1 | 33 | 1 |  |
|  |  |  |  |  |  |  |  | Apply it on scabies | Anti scabbies | 1 | 33 | 1 |  |
| *Zingiberaceae* | *Curcuma amada* Roxb | *ND* | *Mango ginger* | Siguizo manga | Sakéy manga |  |  |  |  |  |  |  | NC |
|  |  |  |  |  |  | Rhizome | Crush | Exfoliation | Skin care | 2 | 34 | 1 |  |
| *Zingiberaceae* | *Curcuma longa* L. | Curcuma | Turmeric | Dzidzano | Tamotamo |  |  |  |  |  |  |  |  |
|  |  |  |  |  |  | Rhizome | Grate the rhizome on a coral stone with a little water using "msindzano" until you obtain a smooth paste | Face | Skin face | 2 | 3 | 1 |  |
|  |  |  |  |  |  |  |  |  | Sun protection | 3 | 3 | 1 |  |
|  |  |  |  |  |  |  |  |  | Anti pimples | 1 | 3 | 1 |  |
|  |  |  |  |  |  |  | Crush the rhizome of *Curcuma longa*, leaves of *Lawsonia inermis*, and roots of *Chrysopogon zizanoïdes*. Mix everything with coconut milk and oil. Let it macerate for a few minutes or hours | Exfoliation | Cleanser | 5 | 4 | 1 |  |
|  |  |  |  |  |  |  |  |  | Beautifies | 5 | 4 | 1 |  |
|  |  |  |  |  |  |  |  |  | Hydrates | 2 | 4 | 1 |  |
|  |  |  |  |  |  |  |  |  | Softens | 2 | 4 | 1 |  |
|  |  |  |  |  |  |  | Crush the leaves and inflorescences of *Ocimum* spp., the rhizome of *Curcuma longa*, the roots of *Chrysopogon zizanoides*, the flowers of *Rosa alba, Pandanus maximus, Jasminum nummulariaefolium*, and *Vachellia farnesiana*. Mix everything with the grated fruit and coconut milk (lait de *Cocos nucifera*). Let it macerate for a few minutes or hours. | Exfoliation | Skin care | 2 | 5 | 1 |  |
|  |  |  |  |  |  |  |  |  | Cleanser | 5 | 5 | 1 |  |
|  |  |  |  |  |  |  |  |  | Softens | 2 | 5 | 1 |  |
|  |  |  |  |  |  |  |  |  | Nourishes | 2 | 5 | 1 |  |
|  |  |  |  |  |  |  |  |  | Beautifies | 5 | 5 | 1 |  |
|  |  |  |  |  |  |  |  |  | Hydrates | 2 | 5 | 1 |  |
|  |  |  |  |  |  |  | Crush the leaves of *Lawsonia inermis,* add the juice of *Citrus* sp. You can optionally add *Curcuma longa* according to taste | Apply using a matchstick on the skin, hands, and feet to create designs | Beautifies | 5 | 6 | 1 |  |
|  |  |  |  |  |  |  | Crush the rhizome of *Curcuma longa*. Add "zoukouba", milk, oil, and grated coconut fruit. Mix everything together | Apply to the skin by rubbing and gently massaging | Cleanser | 5 | 6 | 1 |  |
|  |  |  |  |  |  |  |  |  | Hydrates | 2 | 6 | 1 |  |
|  |  |  |  |  |  |  |  |  | Nourishes | 2 | 6 | 1 |  |
|  |  |  |  |  |  |  |  |  | Softens | 2 | 6 | 1 |  |
|  |  |  |  |  |  |  |  |  | Add shine | 3 | 6 | 1 |  |
|  |  |  |  |  |  |  | Crush the leaves of *Lawsonia inermis* and the rhizome of *Curcuma longa*, then mix them with coconut oil, milk, and grated coconut fruit from *Cocos nucifera* | Massage (body) | Cleanser | 5 | 8 | 1 |  |
|  |  |  |  |  |  |  |  |  | Hydrates | 2 | 8 | 1 |  |
|  |  |  |  |  |  |  |  |  | Nourishes | 2 | 8 | 1 |  |
|  |  |  |  |  |  |  |  |  | Softens | 2 | 8 | 1 |  |
|  |  |  |  |  |  |  |  |  | Beautifies | 5 | 8 | 1 |  |
|  |  |  |  |  |  |  | Crush the leaves of *Lawsonia inermis* and the rhizome of *Curcuma longa*. Mix it all with petroleum | Body / skin | Cleanser | 5 | 9 | 1 |  |
|  |  |  |  |  |  |  |  |  | Beautifies | 5 | 9 | 1 |  |
|  |  |  |  |  |  |  | Crush the rhizome of *Curcuma longa* and mix it with the scented oil composed of *Cocos nucifera* oil, roots of *Chrysopogon zizanoides*, flowers of *Pandanus maximus, Jasminum nummulariaefolium*, and fragrant plants from "salika" | Apply to the skin by rubbing and gently massaging | Cleanser | 5 | 9 | 1 |  |
|  |  |  |  |  |  |  |  |  | Beautifies | 5 | 9 | 1 |  |
|  |  |  |  |  |  |  | Scrape the rhizome of *Curcuma longa* with the piece of wood on the dead coral stone with a little water until a smooth paste is obtained | Face | Sun protection | 3 | 10 | 1 |  |
|  |  |  |  |  |  |  |  |  | Anti pimples | 1 | 10 | 1 |  |
|  |  |  |  |  |  |  |  |  | Beauty mask | 2 | 10 | 1 |  |
|  |  |  |  |  |  |  |  |  | Beautifies | 5 | 10 | 1 |  |
|  |  |  |  |  |  |  | Crush the rhizome of *Curcuma longa*, extract the gel from *Aloe vera*, and cook the milk of *Cocos nucifera* to retrieve the oil. Mix all the ingredients with "zoukouba" | Massage | Massage | 2 | 12 | 1 |  |
|  |  |  |  |  |  |  |  | Massage | Cleanser | 5 | 12 | 1 |  |
|  |  |  |  |  |  |  | Scrape the rhizome of *Curcuma longa* or apply *Curcuma longa* powder on the coral stone using the piece of msindzano wood with a little water until a smooth paste is obtained | Face | Beautifies | 5 | 13 | 1 |  |
|  |  |  |  |  |  |  |  |  | Sun protection | 3 | 13 | 1 |  |
|  |  |  |  |  |  |  | Mix the milk of *Cocos nucifera* and combine it with *Curcuma longa* | Apply on the body and leave it on overnight, then wash off with warm water in the morning (body) | Cleanser | 5 | 13 | 1 |  |
|  |  |  |  |  |  |  |  |  | Lightens | 3 | 13 | 1 |  |
|  |  |  |  |  |  |  |  |  | Add shine | 3 | 14 | 1 |  |
|  |  |  |  |  |  |  |  |  | Beautifies | 5 | 14 | 1 |  |
|  |  |  |  |  |  |  |  | Apply on the body and leave it on overnight, then wash off with warm water in the morning (face) | Add shine | 3 | 14 | 1 |  |
|  |  |  |  |  |  |  |  |  | Beautifies | 5 | 14 | 1 |  |
|  |  |  |  |  |  |  | Crush the rhizome of *Curcuma longa* or mix *Curcuma longa* powder with the milk of *Cocos nucifera* | Apply to the body, massaging gently, and let it sit for a few minutes. Rinse off | Cleanser | 5 | 16 | 1 |  |
|  |  |  |  |  |  |  |  |  | Lightens | 3 | 16 | 1 |  |
|  |  |  |  |  |  |  |  |  | Add shine | 3 | 16 | 1 |  |
|  |  |  |  |  |  |  |  | Apply to the face, massaging gently, and let it sit for a few minutes. Rinse off | Cleanser | 5 | 16 | 1 |  |
|  |  |  |  |  |  |  |  |  | Lightens | 3 | 16 | 1 |  |
|  |  |  |  |  |  |  |  |  | Add shine | 3 | 16 | 1 |  |
|  |  |  |  |  |  |  | Crush the leaves of *Lawsonia inermis* and the rhizome of *Curcuma longa*, and mix them with the milk of *Cocos nucifera* | Apply to the body, massaging gently. | Cleanser | 5 | 16 | 1 |  |
|  |  |  |  |  |  |  |  |  | Lightens | 3 | 16 | 1 |  |
|  |  |  |  |  |  |  |  |  | Add shine | 3 | 16 | 1 |  |
|  |  |  |  |  |  |  |  | Apply to the face, massaging gently. | Cleanser | 5 | 16 | 1 |  |
|  |  |  |  |  |  |  |  |  | Lightens | 3 | 16 | 1 |  |
|  |  |  |  |  |  |  |  |  | Add shine | 3 | 16 | 1 |  |
|  |  |  |  |  |  |  | Crush the leaves of *Lawsonia inermis* and the rhizome of *Curcuma longa*, and mix everything with the juice of *Citrus* sp. | Apply on the hands, creating designs if desired. Leave on for a few hours, then rinse | Beautifies | 5 | 16,27 | 2 |  |
|  |  |  |  |  |  |  |  | Apply on the feet, creating designs if desired. Leave on for a few hours, then rinse | Beautifies | 5 | 16,27 | 2 |  |
|  |  |  |  |  |  |  | Crush the leaves of *Lawsonia inermis*, extract the milk from *Cocos nucifera*, and crush the *Curcuma longa*. Mix everything together | Massage (body) | Beautifies | 5 | 17 | 1 |  |
|  |  |  |  |  |  |  |  |  | Tanned complexion | 3 | 17 | 1 |  |
|  |  |  |  |  |  |  |  |  | Lightens | 3 | 17 | 1 |  |
|  |  |  |  |  |  |  |  | Massage (face) | Beautifies | 5 | 17 | 1 |  |
|  |  |  |  |  |  |  |  |  | Tanned complexion | 3 | 17 | 1 |  |
|  |  |  |  |  |  |  |  |  | Lightens | 3 | 17 | 1 |  |
|  |  |  |  |  |  |  | Crush the rhizome of *Curcuma longa* and mix it with the oil and coconut milk of *Cocos nucifera* | Massage (body) | Cleanser | 5 | 20 | 1 |  |
|  |  |  |  |  |  |  |  |  | Beautifies | 5 | 20 | 1 |  |
|  |  |  |  |  |  |  |  |  | Nourishes | 2 | 20 | 1 |  |
|  |  |  |  |  |  |  |  |  | Lightens | 2 | 20 | 1 |  |
|  |  |  |  |  |  |  | Crush the rhizome of *Curcuma longa,* add the "zoukouba", and coconut milk. Mix everything together | Exfoliation | Cleanser | 5 | 21 | 1 |  |
|  |  |  |  |  |  |  |  |  | Nourishes | 2 | 21 | 1 |  |
|  |  |  |  |  |  |  | Crush the leaves of *Lawsonia inermis* and the roots of *Chrysopogon zizanoides*, and the rhizome of *Curcuma longa*. Mix everything with the oil and milk of *Cocos nucifera* | Exfoliation | Cleanser | 5 | 23 | 1 |  |
|  |  |  |  |  |  |  |  |  | Beautifies | 5 | 23 | 1 |  |
|  |  |  |  |  |  |  |  |  | Hydrates | 2 | 23 | 1 |  |
|  |  |  |  |  |  |  |  |  | Softens | 2 | 23 | 1 |  |
|  |  |  |  |  |  |  | Crush the leaves of *Lawsonia inermis*, crush the rhizome, or add the powder of *Curcuma longa* and the oil of *Sesamum indicum* | Apply by massaging onto the body, performing gentle massages, and rinse with warm water. | Cleanser | 5 | 26 | 1 |  |
|  |  |  |  |  |  |  |  |  | Beautifies | 5 | 26 | 1 |  |
|  |  |  |  |  |  |  |  |  | Nourishes | 2 | 26 | 1 |  |
|  |  |  |  |  |  |  |  |  | Lightens | 3 | 26 | 1 |  |
|  |  |  |  |  |  |  |  | Apply by massaging onto the face, performing gentle massages, and rinse with warm water. | Cleanser | 5 | 26 | 1 |  |
|  |  |  |  |  |  |  |  |  | Beautifies | 5 | 26 | 1 |  |
|  |  |  |  |  |  |  |  |  | Nourishes | 2 | 26 | 1 |  |
|  |  |  |  |  |  |  |  |  | Lightens | 3 | 26 | 1 |  |
|  |  |  |  |  |  |  | Crush the rhizome of *Curcuma longa* and mix it with the pulp of the fruit of *Adansonia digitata* and the milk and grated fruit of *Cocos nucifera* until obtaining a fairly smooth paste | Apply on the face, gently massaging, let it rest for a few minutes. Rinse | Beautifies | 5 | 27 | 1 |  |
|  |  |  |  |  |  |  |  |  | Well-being | 5 | 27 | 1 |  |
|  |  |  |  |  |  |  |  |  | Tanned complexion | 3 | 27 | 1 |  |
|  |  |  |  |  |  |  |  |  | Lightens | 3 | 27 | 1 |  |
|  |  |  |  |  |  |  |  |  | Hydrates | 2 | 27 | 1 |  |
|  |  |  |  |  |  |  |  |  | Softens | 2 | 27 | 1 |  |
|  |  |  |  |  |  |  |  |  | Anti-aging | 2 | 27 | 1 |  |
|  |  |  |  |  |  |  |  | Apply on the body , gently massaging, let it rest for a few minutes. Rinse | Beautifies | 5 | 27 | 1 |  |
|  |  |  |  |  |  |  |  |  | Well-being | 5 | 27 | 1 |  |
|  |  |  |  |  |  |  |  |  | Tanned complexion | 3 | 27 | 1 |  |
|  |  |  |  |  |  |  |  |  | Lightens | 3 | 27 | 1 |  |
|  |  |  |  |  |  |  |  |  | Hydrates | 2 | 27 | 1 |  |
|  |  |  |  |  |  |  |  |  | Softens | 2 | 27 | 1 |  |
|  |  |  |  |  |  |  |  |  | Anti-aging | 2 | 27 | 1 |  |
|  |  |  |  |  |  |  | Scrape the rhizome or place the powder on the coral stone with "msindzano" and a little water until obtaining a fairly smooth paste | Face | Anti pimples | 1 | 32 | 1 |  |
|  |  |  |  |  |  |  |  |  | Beautifies | 5 | 32 | 1 |  |
|  |  |  |  |  |  |  |  |  | Anti -aging | 2 | 32 | 1 |  |
|  |  |  |  |  |  |  | Crush the leaves of *Lawsonia inermis* and the rhizome of *Curcuma longa*, mix with *Sesamum indicum* oil | Exfoliation | Cleanser | 5 | 32 | 1 |  |
|  |  |  |  |  |  |  |  |  | Lightens | 3 | 32 | 1 |  |
|  |  |  |  |  |  |  |  |  | Anti -aging | 2 | 32 | 1 |  |
|  |  |  |  |  |  |  |  |  | Softens |  | 32 | 1 |  |
|  |  |  |  |  |  |  |  |  | Tightens | 3 | 32,32 | 2 |  |
| *Zingiberaceae* | *Zingiber officinale* Roscoe | Gingembre | Ginger | Siguizo | Sakéy |  |  |  |  |  |  |  | NC |
|  |  |  |  |  |  | Rhizome | Crush the leaves of Ocimum gratissimum, Lantana trifolia, and the rhizome of Zingiber officinale, and form a ball | Insert the ball into the vagina and position yourself above a heated stone | Tightens | 5 | 15 | 1 |  |
| *Zingiberaceae* | *Zingiber zerumbet* (L.) Sm. | Amome sauvage | Shampoo ginger | Singuizo masera | Sakéyi loulou |  |  |  |  |  |  |  | NC |
|  |  |  |  |  |  | Flower | Collect the water from the flowers | Apply to the hair, massage, and rinse | Shampoo | 4 | 17 | 1 |  |
|  |  |  |  |  |  |  |  | Face | Cleanser | 5 | 17 | 1 |  |
| NA | ND | ND | ND | Koukoumba | Koukoumba |  |  |  |  |  |  |  | NC |
|  |  |  |  |  |  | Flower | Prick the leaves and inflorescences of *Ocimum* spp. with "koukoumba" flowers using a pin and form a heap on it | Hair | Beautifies | 5 | 4 | 1 |  |
|  |  |  |  |  |  |  |  |  | Fragrance | 5 | 4 | 1 |  |
|  |  |  |  |  |  |  | Not preparation recquired | Place on the bed | Fragrance | 5 | 4,7,11,23,23,32 | 6 |  |
|  |  |  |  |  |  |  | Take the mixture of plants obtained from marriages or create your own blend based on plant availability (Roots of *Chrysopogon zizanoides*, Leaves of *Ayapana triplinervis*, Leaves and inflorescences of *Ocimum* spp., Flowers of *Jasminum nummulariaefolium, Vachellia farnesiana, Rosa alba*, "Koukoumba", P*lumeria alba, Cananga odorata, Pandanus maximus*). Once dried, crush and sort them by size. Prepare the coconut oil. To do this, grate the coconuts, extract the milk, let it sit overnight, remove the cream on top, and then cook it in a large pot over a wood fire. Once the oil is ready, add the flower mixture "zoukouba" when the temperature has cooled slightly, and add the pompeia when the oil is no longer very hot. Let it all macerate. The longer the maceration, the more intense the fragrance will be | Body / skin | Nourishes | 2 | 6 | 1 |  |
|  |  |  |  |  |  |  |  |  | Add shine | 3 | 6 | 1 |  |
|  |  |  |  |  |  |  |  |  | Softens | 2 | 6 | 1 |  |
|  |  |  |  |  |  |  |  |  | Fragrans | 5 | 6 | 1 |  |
|  |  |  |  |  |  |  |  |  | Lightens | 3 | 6 | 1 |  |
|  |  |  |  |  |  |  |  |  | Hydrates | 2 | 6 | 1 |  |
|  |  |  |  |  |  |  | Prick the flowers according to the availability and desire of J*asminum nummulariefolium, Pandanus maximus, Vachellia farnesiana, Cananga odorata, Rosa alba,"* Koukoumba", *Cestrum nocturnum* using a pin and form a heap on it | Hair | Beautifies | 5 | 7 | 1 |  |
|  |  |  |  |  |  |  |  | Hair | Fragrance | 5 | 7 | 1 |  |
|  |  |  |  |  |  |  | Put on "kilabou" | Hair | Beautifies | 5 | 23 | 1 |  |
|  |  |  |  |  |  |  | Put on "kilabou" | Hair | Fragrance | 5 | 23 | 1 |  |
|  |  |  |  |  |  |  | Place on safety pins a mixture of leaves and inflorescences of *Ocimum* spp. and J*asminum nummulariaefolium* | Hair | Beautifies | 5 | 32 | 1 |  |
|  |  |  |  |  |  |  |  |  | Fragrance | 5 | 32 | 1 |  |
|  |  |  |  |  |  |  |  | Clothing | Beautifies | 5 | 32 | 1 |  |
|  |  |  |  |  |  |  |  |  | Fragrance | 5 | 32 | 1 |  |
|  |  |  |  |  |  |  | Place on safety pins a mixture of leaves and inflorescences of *Ocimum* spp. and J*asminum nummulariaefolium* | Neck | Beautifies | 5 | 32 | 1 |  |
|  |  |  |  |  |  |  |  |  | Fragrance | 5 | 32 | 1 |  |
| NA | ND | ND | ND | ND | Lala viti, Fagnéva mzouzka |  |  |  |  |  |  |  | *ODAM024* |
|  |  |  |  |  |  | Leaf | Crush the leaves in water by hand and filter | Take the comb and put it in the mixture, comb your hair with the mixture. Then rinse your hair with warm water | Softens | 4 | 9 | 1 |  |
|  |  |  |  |  |  |  |  |  | Detangles | 4 | 9 | 1 |  |
|  |  |  |  |  |  |  |  |  | Stimulates hair growth | 4 | 9 | 1 |  |
| ND | ND | ND | ND | Muté | ND |  |  |  |  |  |  |  | NC |
|  |  |  |  |  |  | Seed | Scratch the piece of "Msindzano" wood and the seeds of "muté" on the dead coral stone with a little water until a smooth paste is obtained | Face | Anti-aging | 2 | 11 | 1 |  |
| ND | ND | ND | ND | Shifundja koli | ND |  |  |  |  |  |  |  | NC |
|  |  |  |  |  |  | Leaf | Crush the leaves until sweating | Body / skin | Dermatoses | 1 | 2 | 1 |  |
|  |  |  |  |  |  |  | Crush or pound the leaves and put them in water, let it sit or mix until thickening | Apply on the hair, clean and comb, put on a scarf, leave for 30 minutes, then rinse with water while combing | Cleanser | 4 | 27 | 1 |  |
|  |  |  |  |  |  |  |  |  | Detangles | 4 | 27 | 1 |  |
|  |  |  |  |  |  |  |  |  | Softens | 4 | 27 | 1 |  |
|  |  |  |  |  |  |  |  |  | Smoothens | 4 | 27 | 1 |  |
|  |  |  |  |  |  |  | Crush the leaves of "shifundja koli" et *Ceiba pentrada* | Shampoo | Stimulates hair growth | 4 | 34 | 1 |  |
|  |  |  |  |  |  |  |  |  | Smoothens | 4 | 34 | 1 |  |
| ND | ND | ND | ND | Muvuvu | Adabu |  |  |  |  |  |  |  | NC |
|  |  |  |  |  |  | Piece of wood | Scratch the piece of wood on the dead coral stone with a little water until a smooth paste is obtained | Body / skin | Anti pimple (Child) | 1 | 2 | 1 |  |
|  |  |  |  |  |  | Latex | Crush the seeds of *Sapindus saponaria* and add the latex of "Adabu" | Body / skin | Anti pimple (Child) | 1 | 18 | 1 |  |
|  |  |  |  |  |  |  | Extract the latex from the tree | Body / skin | Anti pimple (Child) | 1 | 34 | 1 |  |
| ND | ND | ND | ND | ND | Kandza mroni |  |  |  |  |  |  |  | NC |
|  |  |  |  |  |  | Leaf | Not preparation recquired | Rub the leaves under the armpits | Deodorant | 1 | 15 | 1 |  |
|  |  |  |  |  |  |  |  |  | Antiperspirant | 1 | 15 | 1 |  |
| ND | ND | ND | ND | ND | Tamotamo hazo |  |  |  |  |  |  |  | NC |
|  |  |  |  |  |  | Seed | Scratch the seeds of "tamotamo hazo" with the piece of "msindzano" wood on the dead coral stone with a little water until a smooth paste is obtained | Face | Tanned complexion | 3 | 4,6,27,32 | 4 |  |
|  |  |  |  |  |  |  |  |  | Beautifies | 5 | 4,13,14,27 | 4 |  |
|  |  |  |  |  |  |  |  |  | Anti-aging | 2 | 4,14,32 | 3 |  |
|  |  |  |  |  |  |  |  |  | Color the "msindzano" | 3 | 6 | 1 |  |
|  |  |  |  |  |  |  |  |  | Sun protection | 3 | 6,13,14,27 | 4 |  |
|  |  |  |  |  |  |  |  |  | Anti pimples | 1 | 6,27,32 | 3 |  |
|  |  |  |  |  |  |  |  |  | Add shine | 3 | 14 | 1 |  |
| ND | ND | ND | ND | Ka | Ka |  |  |  |  |  |  |  | NC |
|  |  |  |  |  |  | Powder | Scrape the "Ka" powder with the piece of msindzano wood on the dead coral stone with a little water until obtaining a smooth paste | Face | Color the "msindzano" | 3 | 4 | 1 |  |
|  |  |  |  |  |  |  |  |  | Sun protection | 3 | 4 | 1 |  |
|  |  |  |  |  |  |  |  |  | Fragrance | 5 | 10 | 1 |  |
|  |  |  |  |  |  |  | Mix coconut milk and coconut flesh with the "Ka" powder | Body / skin | Cleanser | 5 | 4 | 1 |  |
|  |  |  |  |  |  |  |  |  | Hydrates | 2 | 4 | 1 |  |
|  |  |  |  |  |  |  |  |  | Softens | 2 | 4 | 1 |  |
|  |  |  |  |  |  |  | Place it in the "masinguou" | Body / skin | Fragrance | 5 | 16 | 1 |  |
|  |  |  |  |  |  |  |  |  | Beautifies | 5 | 16 | 1 |  |
|  |  |  |  |  |  | Piece of wood | Scrape the piece of wood of "Ka" with the piece of msindzano wood on the dead coral stone with a little water until obtaining a smooth paste | Face | Beautifies | 5 | 27 | 1 |  |
|  |  |  |  |  |  |  |  |  | Fragrance | 5 | 27 | 1 |  |
|  |  |  |  |  |  |  |  |  | Sun protection | 3 | 27 | 1 |  |
|  |  |  |  |  |  |  |  |  |  |  |  |  |  |
|  |  |  |  |  |  |  |  |  |  |  |  |  |  |
| ND | ND | "Bois de santal" | Sandalwood | Msindzano | Msindzano |  |  |  |  |  |  |  | NC |
|  |  |  |  |  |  | Piece of wood | Crush 1 or 2 cloves of *Syzygium aromaticum* with "msindzano" using a little water on the coral stone until obtaining a smooth paste | Face | Cleanser | 5 | 9 | 1 |  |
|  |  |  |  |  |  |  |  |  | Beautifies | 5 | 9 | 1 |  |
|  |  |  |  |  |  |  |  |  | Fragrance | 5 | 9 | 1 |  |
|  |  |  |  |  |  |  | Crush the seeds of "Tamotamo hazo" on the coral stone with the piece of "msindzano" wood, using a little water until obtaining a fairly smooth paste | Face | Beautifies | 5 | 13 | 1 |  |
|  |  |  |  |  |  |  |  |  | Sun protection | 3 | 13 | 1 |  |
|  |  |  |  |  |  |  | Scrape the powder of "Ka" with the piece of "msindzano" wood on the dead coral stone, using a little water until obtaining a smooth paste | Face | Fragrance | 5 | 4 | 1 |  |
|  |  |  |  |  |  |  |  |  | Fragrance | 5 | 4 | 1 |  |
|  |  |  |  |  |  |  | Scrape the piece of "Msindzano" wood and the seeds of "Muté" on the dead coral stone, using a little water until obtaining a smooth paste. | Face | Anti -aging | 2 | 11 | 1 |  |
|  |  |  |  |  |  |  | Scrape the piece of wood and the "manu kantru" (flower mixture) on the dead coral stone until obtaining a smooth paste | Face | Sun protection | 3 | 19 | 1 |  |
|  |  |  |  |  |  |  |  |  | Beautifies | 5 | 19 | 1 |  |
|  |  |  |  |  |  |  |  |  | Fragrance | 5 | 19 | 1 |  |
|  |  |  |  |  |  |  |  | Body / skin | Beautifies | 5 | 19 | 1 |  |
|  |  |  |  |  |  |  |  |  | Fragrance | 5 | 19 | 1 |  |
|  |  |  |  |  |  |  | Scrape the piece of wood on the dead coral stone with a little water until obtaining a smooth paste | Face | Anti pimples | 1 | 1,4,6,7,10,12,23,33 | 8 |  |
|  |  |  |  |  |  |  |  |  | Anti -aging | 2 | 13,15,32,16 | 4 |  |
|  |  |  |  |  |  |  |  |  | Anti spot | 3 | 12,17 | 2 |  |
|  |  |  |  |  |  |  |  |  | Anti pimples | 1 | 30,32,35,27 | 4 |  |
|  |  |  |  |  |  |  |  |  | Reduces irritation | 1 | 30 | 1 |  |
|  |  |  |  |  |  |  |  |  | Refreshes | 5 | 30 | 1 |  |
|  |  |  |  |  |  |  |  |  | Lightens | 3 | 16 | 1 |  |
|  |  |  |  |  |  |  |  |  | Beautifies | 5 | 1,7,10,13,15,16,17,19,20,23,32,27 | 12 |  |
|  |  |  |  |  |  |  |  |  | Softens | 2 | 17 | 1 |  |
|  |  |  |  |  |  |  |  |  | Beauty mask | 2 | 10,12 | 2 |  |
|  |  |  |  |  |  |  |  |  | Sun protection | 3 | 1,3,4,7,10,12,16,17,23 | 9 |  |
|  |  |  |  |  |  |  |  | Body / skin | Redness in (baby) | 1 | 1,19,20,19,20 | 5 |  |
|  |  |  |  |  |  |  |  |  | Anti pimples | 1 | 30,3,4,6,19,20,19,20,23,23,26,26,15 | 13 |  |
|  |  |  |  |  |  |  |  |  | Reduces irritation | 1 | 30 | 1 |  |
|  |  |  |  |  |  |  |  |  | Refreshes | 5 | 30 | 1 |  |
|  |  |  |  |  |  |  |  |  | Sun protection | 3 | 19,20,23 | 3 |  |
|  |  |  |  |  |  |  |  |  | Beautifies | 5 | 19,20,23,26 | 4 |  |
|  |  |  |  |  |  |  |  |  | Anti -aging | 2 | 26 | 1 |  |
|  |  |  |  |  |  |  |  | Apply on the face as foundation ==> Put the msindzano when it starts to dry, gently rub to leave a fine powder | Foundation | 5 | 6 | 1 |  |
|  |  |  |  |  |  |  |  | Apply thickly on the face | Sun protection | 3 | 6 | 1 |  |
|  |  |  |  |  |  |  |  |  | Beautifies | 5 | 6 | 1 |  |
|  |  |  |  |  |  |  |  | Acne spots | Anti pimples | 1 | 15 | 1 |  |
|  |  |  |  |  |  |  | Scrape the rhizome of *Curcuma longa* with the piece of wood on the dead coral stone, using a little water until obtaining a smooth paste | Face | Sun protection | 3 | 10 | 1 |  |
|  |  |  |  |  |  |  |  |  | Anti pimples | 1 | 10 | 1 |  |
|  |  |  |  |  |  |  |  |  | Beauty mask | 2 | 10 | 1 |  |
|  |  |  |  |  |  |  |  |  | Beautifies | 5 | 10 | 1 |  |
|  |  |  |  |  |  |  | Scrape the rhizome of *Curcuma longa* or place the powder of *Curcuma longa* on the coral stone with the piece of "msindzano" wood, using a little water until obtaining a fairly smooth paste | Face | Beautifies | 5 | 13,32 | 2 |  |
|  |  |  |  |  |  |  |  |  | Sun protection | 3 | 13 | 1 |  |
|  |  |  |  |  |  |  |  |  | Anti pimples | 1 | 32 | 1 |  |
|  |  |  |  |  |  |  |  |  | Anti -aging | 2 | 32 | 1 |  |
|  |  |  |  |  |  |  | Scrape the seeds of "tamotamo hazo" with the piece of "msindzano" wood on the dead coral stone, using a little water until obtaining a smooth paste | Face | Tanned complexion | 3 | 4,14,27 | 3 |  |
|  |  |  |  |  |  |  |  |  | Beautifies |  | 4,27,32 | 3 |  |
|  |  |  |  |  |  |  |  |  | Anti -aging |  | 4,14,32 | 3 |  |
|  |  |  |  |  |  |  |  |  | Add shine |  | 14 | 1 |  |
|  |  |  |  |  |  |  |  |  | Sun protection | | 14,27 | 2 |  |
|  |  |  |  |  |  |  |  |  | Anti pimples |  | 32 | 1 |  |
|  |  |  |  |  |  |  | Scrape a few seeds of "tamotamo hazo" with the piece of wood and a little water until obtaining a paste of reddish-orange color according to preferences. | Face | Color "msindzano" | 3 | 6 | 1 |  |
|  |  |  |  |  |  |  |  |  | Tanned complexion | 3 | 6 | 1 |  |
|  |  |  |  |  |  |  |  |  | Sun protection | 3 | 6 | 1 |  |
|  |  |  |  |  |  |  |  |  | Anti pimples | 1 | 6 | 1 |  |
| ND | ND | ND | ND | ND | Sandraouri |  |  |  |  |  |  |  | NC |
|  |  |  |  |  |  | Leaf | Crush the leaves of "Sandraouri" and "Andra béyi layi". Mix with *Cocos nucifera* oil | Face | Anti pimples. Caution: Possibility of allergies. Test on the skin before application | 1 | 18 | 1 |  |
| ND | ND | ND | ND | Hasa mtroubaba | Andra béyi layi |  |  |  |  |  |  |  | NC |
|  |  |  |  |  |  | Leaf | Crush the leaves of "Sandraouri" and "Andra béyi layi". Mix with *Cocos nucifera* oil | Face | Anti pimples. Caution: Possibility of allergies. Test on the skin before application | 1 | 18 | 1 |  |
|  |  |  |  | Romba |  |  |  |  |  |  |  |  | NC |
|  |  |  |  |  |  | Leaf | Crush the leaves of "romba" with *Citrus* sp. | Massage (body) and wash | Stimulates | 5 | 20 | 1 |  |
|  |  |  |  |  |  |  |  |  | Tones | 5 | 20 | 1 |  |
|  |  |  |  |  |  |  |  |  | Energyzing | 5 | 20 | 1 |  |
|  |  |  |  |  | Vahi tampa |  |  |  |  |  |  |  | NC |
|  |  |  |  |  |  | Root | Scrape the root of *"*vahi tampa" on a piece of dead coral with a little water until obtaining a paste | Wound | Wound healing | 1 | 20 | 1 |  |
|  |  |  |  |  |  |  | Scrape the roots of "vahi tampa"," sadrakidraki", and pouropetka on a piece of dead coral with a little water until obtaining a paste | Pimples | Anti pimples | 1 | 20 | 1 |  |
|  |  |  |  |  | Pouroupetka |  |  |  |  |  |  |  | NC |
|  |  |  |  |  |  | Root | Scrape the roots of "vahi tampa"," sadrakidraki", and pouropetka on a piece of dead coral with a little water until obtaining a paste | Pimples | Anti pimples | 1 | 20 | 1 |  |
|  |  |  |  |  | Sari féliki guissi |  |  |  |  |  |  |  | NC |
|  |  |  |  |  |  | Leaf | Crush the leaves in water | Apply to the affected area with a clean cloth or cotton | Reddish pimples with patches. Toxic plants can cause dizziness and stomach ache. Do not apply the mixture in case of allergies. Test on a small area of the skin before applying | 1 | 26 |  |  |
|  |  |  |  |  |  |  |  | Apply to the affected area | Pimples with lesions. Toxic plants | 1 | 35 |  |  |
| ND | ND | ND | ND | Mwana mwéhou | ND |  |  |  |  |  |  |  | NC |
|  |  |  |  |  |  | Seed | Burst the seed and retrieve the interior, scrape it on the coral stone with a little water until obtaining a paste that will be white in color, somewhat like white clay | Face | Beautifies | 5 | 27 | 1 |  |
|  |  |  |  |  |  |  |  |  | Softens | 2 | 27 | 1 |  |
| ND | ND | ND | ND | Shimamaba | ND |  |  |  |  |  | 27 | 1 | NC |
|  |  |  |  |  |  | Piece of wood | Scrape the piece of wood on the coral stone with a little water until obtaining a paste | Body / skin at bedtime, let it dry | Beautifies | 5 | 27 | 1 |  |
|  |  |  |  |  |  |  |  |  | Fragrance | 5 | 27 | 1 |  |
|  |  |  |  |  |  |  |  |  | Tones | 5 | 27 | 1 |  |

Legend: ND for “not determined”, NC for “not collected”, plants not collected were identified based on their vernacular names by referring to “La flore illustrée de Mayotte”(Barthelat, 2019). Symbol “/” is used for “and”.
